# Functional Annotation of Small Noncoding RNAs Target Genes Provides Evidence for a Deregulated Ubiquitin-Proteasome Pathway in Spinocerebellar Ataxia Type 1

**DOI:** 10.1155/2012/672536

**Published:** 2012-10-03

**Authors:** Stephan Persengiev, Ivanela Kondova, Ronald E. Bontrop

**Affiliations:** ^1^Department of Medical Genetics, University Medical Center Utrecht, 3584 CG Utrecht, The Netherlands; ^2^Animal Science Department, Biomedical Primate Research Centre, Lange Kleiweg 139, 2288 GJ Rijswijk, The Netherlands; ^3^Department of Comparative Genetics and Refinement, Biomedical Primate Research Center, Lange Kleiweg 139, 2288 GJ Rijswijk, The Netherlands

## Abstract

Spinocerebellar ataxia type 1 (SCA1) is a neurodegenerative disorder caused by the expansion of CAG repeats in the ataxin 1 (ATXN1) gene. In affected cerebellar neurons of patients, mutant ATXN1 accumulates in ubiquitin-positive nuclear inclusions, indicating that protein misfolding is involved in SCA1 pathogenesis. In this study, we functionally annotated the target genes of the small noncoding RNAs (ncRNAs) that were selectively activated in the affected brain compartments. The primary targets of these RNAs, which exhibited a significant enrichment in the cerebellum and cortex of SCA1 patients, were members of the ubiquitin-proteasome system. Thus, we identified and functionally annotated a plausible regulatory pathway that may serve as a potential target to modulate the outcome of neurodegenerative diseases.

## 1. Introduction

Aging is accompanied by cognitive decline in a major part of the population and is the primary risk factor for a number of neurodegenerative disorders. Aging-related neurodegenerative disorders are the culmination of many different genetic and environmental influences ultimately leading to the degeneration of specific neurons. The regulatory mechanisms controlling the expression of a number of genes may be altered during the course of certain neurodegenerative disorders, and recent evidence has indicated that small non-coding RNAs (snRNAs) and miRNAs might be a significant risk factor in neurodegeneration, including Alzheimer's and Parkinson's disease, spinocerebellar ataxia type 1 (SCA1), and triplet repeat disorders [1, 2]. A critical process in the development SCA1 is the deregulation of genes that affect neuronal cell survival mechanisms. In the case of SCA1 the expansion of the glutamine-rich region of ataxin-1 results in the accumulation of insoluble protein aggregates that are the main cause for the disease symptoms [3]. This view has recently been challenged by findings that, for some of these diseases, neurological symptoms begin appearing before protein aggregates form, or even when aggregates do not form at all [4]. These findings have led to the conclusion that accumulation of mutant ataxin-1 may be facilitated by the activation or deactivation of selective cell survival mechanism in the vulnerable neurons. 

Identifying the regulatory circuitry processes that control cell differentiation and transmission of information between neurons is fundamental to understanding changes in the aging brain. miRNAs regulate expression of protein-coding genes [5, 6]. Several lines of evidence indicate that miRNAs contribute to the control of brain development and its functional and structural reorganization, as a result of age progression and deterioration of neuronal metabolism. A subset of miRNAs is selectively expressed in brain tissues [7] and targeted inactivation of Dicer miRNA processing endonuclease was found to lead to degeneration of Purkinje cells [6]. In addition, retinal cells deficient for Dicer undergo a progressive degeneration [5]. Specific miRNAs have been shown to be involved in Alzheimer's disease and other neurodegenerative pathologies [8–10]. However, how miRNA expression is regulated during brain aging and how miRNAs participate in the regulatory circuitries that are affected during the neurodegeneration are not understood.

In this paper, we describe the signature pattern of a defined set of small non-coding RNAs in the brain of SCA1 patients as compared to healthy aged individuals. The systematic functional annotation of the potential protein-coding targets enabled us to identify genes with altered expression patterns in the affected brain compartments of SCA1-affected patients. Our results reveal an undescribed essential proteasome regulatory pathway in SCA1 disease, which opens avenues for therapeutic intervention.

## 2. Materials and Methods

### 2.1. Tissue Collection Analysis of Tissue Morphology

Human brain-region tissue from eight individuals was obtained form the German and Dutch Brain Banks. In all cases, the individuals suffered sudden death for reasons not associated with either their participation in this study or with the tissues used. SCA1 brain samples were obtained from Dr. Arnulf Koeppen at VA Medical Center, Albany, NY. The ages and gender of all individuals are listed in Supplementary Table 5 in Supplementary Material available online at doi: 10.1155/2012/672536. 

Frozen brain samples from the human subjects were obtained and stored at −80°C for further analyses. For the purpose of our analysis sections of the frontal cortex and cerebellum were routinely examined by light microscopy.

### 2.2. miRNA Microarray

Total RNA, including miRNA, was purified from different brain regions by the miRNeasy isolation kit (Qiagen). RNA quality was evaluated by the Bio-Rad Experion Automated Electrophoresis System (Bio-Rad, USA). Microarray-based miRNA expression profiling was performed using miRCURY LNA human microRNA Array (Exiqon, USA). The microarrays contained approximately 1200 assay probes corresponding to all the annotated and nonannotated human miRNA sequences (miRBase, version 12, 2008; The Wellcome Trust Sanger Institute, Cambridgeshire, UK). Total RNA labeling and hybridization were performed under standard conditions according to the manufacturer's instructions. The raw data has been deposited in the Array Express database http://www.ebi.ac.uk/arrayexpress/ under accession number E-MTAB-852.

### 2.3. Cells Cultures

HeLa and HEK293T cells were obtained from American Type Culture Collection (ATCC, Manassas, VA, USA) and were propagated at 37°C in DMEM (Life Technologies) supplemented with 10% fetal bovine serum, 100 U/mL penicillin, and 100 U/mL streptomycin. Cells were passed regularly to maintain exponential growth. 

### 2.4. RT-PCRs

Total RNA from human brain regions and HEK393T cells was prepared using the miRNAEasy kit (Qiagen). cDNA synthesis was carried out with both Superscript II (Invitrogen) and oligo-dT primers according to the manufacturer's instructions. The primers used for the detection of human HECTD1, RNF8, PJA2, UBE2W, CD40, TCBA1, MLL, and GAPDH mRNA levels are shown in Supplementary Table 4.

### 2.5. HECTD1 3′UTR Cloning

The isolation of the entire human HECTD1 3′UTR was accomplished by PCR amplification of genomic DNA using specific primers (Supplementary Table 4). The amplified product was first cloned into pCR2 vector (Promega), and recloning into pGL3-control vector was carried out using the Xba I cloning site. The construct was sequenced to verify HECTD1 sequence identity. 

### 2.6. Cell Transfections and Dual Luciferase Assay

HeLa and HEK293T cells were plated the day before transfection at 2 × 10^4^ cells per well in 12-well plates. The following day, 50 pmoles of miRNA duplexes and 100 ng of HECTD1-3′UTR-hLuc were cotransfected into the cells using Lipofectamine 2000 (Invitrogen) according to the manufacturer's instructions. The sequences of miRNA duplexes are E1016-F; 5′-ccaaugauguaaugauucugcc-3′, E1016-R; 5′-ggcagaaucauuacaucauugg-3′, E1108-F; 5′-aauguuuagacgggcucac-3′, E1108-R; 5′-gugagcccgucuaaacauu-3′.

All of the miRNA duplexes were purchased from Dharmacon (Lafayette, CO, USA). Human HECTD1 siRNAs were purchased from Sigma (USA). Cotransfection experiments of HEK293T and HeLa cells with pGL3-HECTD1-3′UTR reporter and miRNA mimics were carried out using Lipofectamine 2000 (Invitrogen). Luciferase assays were performed after 72 h using the Dual Luciferase Reporter Assay System (Promega) according to the manufacturer's protocols. Transfections of HEK293T with GFP-ATXN1 and GFP-ATXN1-82Q reporters were carried out using Fugene (Roche). The experiments were performed in triplicates.

### 2.7. Cell Proliferation Assay

HEK293T cells were cotransfected with either GFP-ATXN1 and GFP-ATXN1-Q86 vectors or HECTD1 siRNA and miR-E1108 mimic. Transfections were carried out using Lipofectamine 2000. The rate of cell proliferation was monitored by cell counting in hemocytometer using the live/death exclusion assay protocol.

### 2.8. Bioinformatics Analysis

Bioinformatics analysis for gene enrichment was performed using the DAVID database (DAVID Functional Annotation Bioinformatics Microarray Analysis, http://david.abcc.ncifcrf.gov/) [11, 12]. Identification of miRNA target genes was performed using the miRNA target search database (http://rna.igmors.u-psud.fr/), the RNA regulatory networks database (http://www.mirz.unibas.ch/), and the TargetScan database (http://www.targetscan.org/) [13–15]. The miRNA precursor sequence and secondary structure were identified using the BLAST alignment search engine (http://blast.ncbi.nlm.nih.gov/Blast.cgi) and the Vienna RNA webserver (http://rna.tbi.univie.ac.at/).

## 3. Results and Discussion

**Figure 1:**
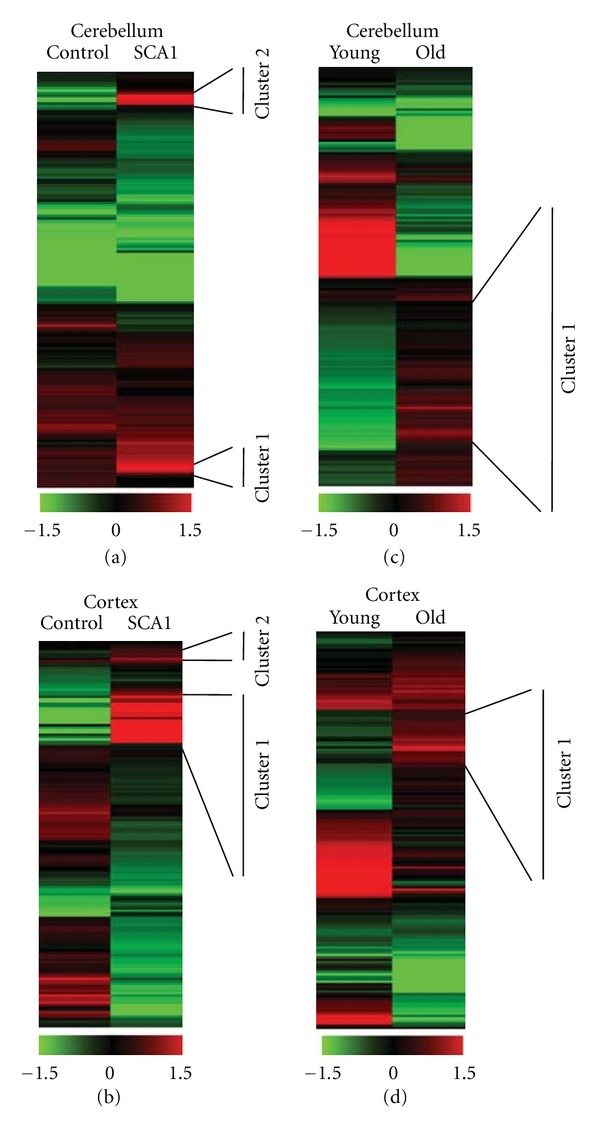
ncRNA expression profiles in the cerebellum and cortex of SCA1 patients and healthy individuals. (a) Heatmap of ncRNA expression profiling comparing the cerebellum of a 61-year-old healthy individual (control) to the cerebellum of an SCA1 patient. SCA1 ncRNAs with increased expression are indicated as cluster 1 and 2. (b) Heatmap of ncRNA expression profiling comparing the frontal cortex of an aging healthy individual (control) to the frontal cortex of an SCA1 patient. SCA1 ncRNAs with increased expression are indicated as cluster 1 and 2. (c) Heatmap of ncRNA expression profiling comparing the cerebellum of a healthy young individual (young) to the cerebellum of an aged healthy subject (old). ncRNAs with increased expression in the aged cerebellum are indicated as cluster 1. (d) Heatmap of ncRNA expression profiling comparing the frontal cortex of a healthy young individual (young) to the frontal cortex of an aged healthy subject (old). ncRNAs with increased expression in the aged cerebellum are indicated as cluster 1.

**Figure 2:**
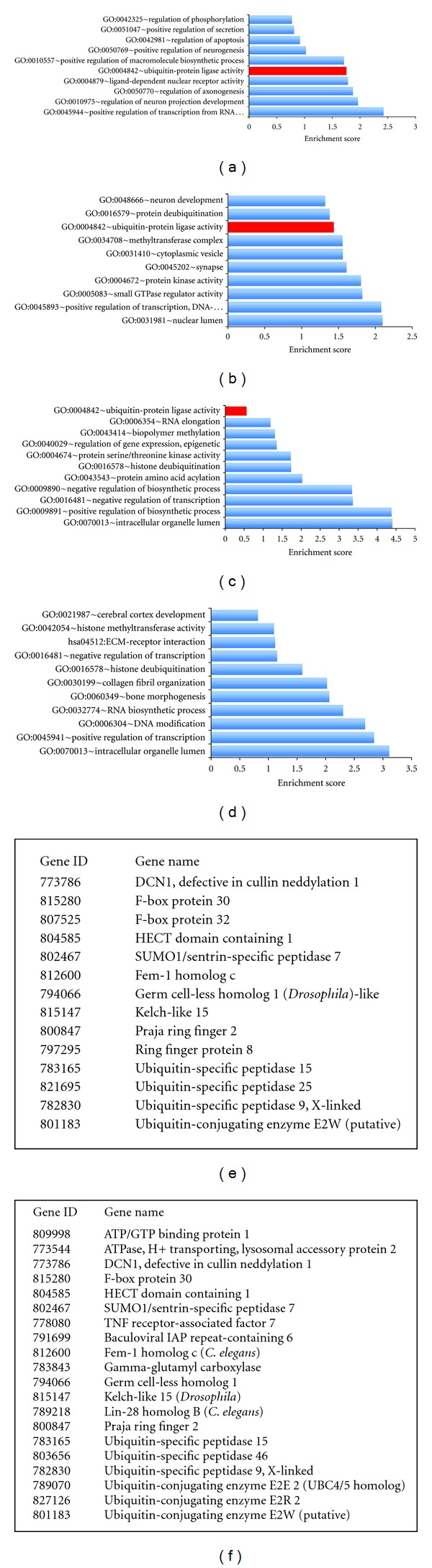
Bioinformatics analysis and annotation of predicted target genes recognized by the upregulated ncRNAs in the cortex and cerebellum of SCA1 subjects and old healthy individuals. (a) Gene set enrichment analysis and functional annotation of ncRNA target genes with significant enrichment score in the cerebellum of an SCA1 patient. (b) Gene set enrichment analysis and functional annotation of ncRNA target genes with a significant enrichment score in the frontal cortex of an SCA1 patient. (c) Gene set enrichment analysis and functional annotation of ncRNA target genes with significant enrichment score in the cerebellum of an aged healthy individual. (d) Gene set enrichment analysis and functional annotation of ncRNA target genes with significant enrichment score in the frontal cortex of an aged healthy individual. (e) Ubl ubiquitination pathway genes with significant enrichment for ncRNA response elements in the cerebellum of SCA1 patient. (f) Ubl ubiquitination pathway genes with significant enrichment for ncRNA response elements in the frontal cortex of SCA1 patient.

We previously mapped miRNA expression patterns in the aging cortex and cerebellum of several nonhuman primate species and in humans by employing the Exiqon microRNA arrays, covering all miRNAs annotated in *miRBase* 11.0 [16]. The data revealed clusters of coregulated miRNA genes with either reduced or increased expression in the frontal cortex and cerebellum of aged individuals and SCA1 patients. In addition to the annotated miRNAs, Exiqon arrays contain 438 novel small noncoding RNAs (ncRNAs). We analyzed the expression profiles of both the miRNAs and non-annotated ncRNAs and found that selected novel ncRNAs formed distinctive upregulated subsets in the cortex and cerebellum of aging human subjects and in SCA1 patients (Figures 1(a), 1(b), 1(c), and 1(d) and Supplementary Tables 1 and 2). Encouraged by this observation, we hypothesized that some of these unreported ncRNAs might be functionally important and play a key role in the regulation of gene networks that evolved relatively late during primate evolution, and, as such, they might be linked to the age-related phenotypes and SCA1 pathogenesis. To further validate and confirm this assumption, we identified all potential target genes of the upregulated ncRNAs and performed functional enrichment analyses of all target genes that were annotated in the DAVID database (Supplementary Tables 6, 7, 8, and 9) [12]. As can be seen, multiple biological pathways appear to be enriched in the cerebellum and cortex of SCA1 patients as compared to the healthy aged controls (Figures 2(a), 2(b), 2(c), and 2(d)). Most notably, the enrichment score for the Ubl conjugation pathway, that comprises of proteins involved in ubiquitin-like modifier protein processing, was significantly elevated in SCA1 tissue (1.75 and 1.43 in SCA1 cerebellum and cortex versus 0.56 and 0 in the controls, resp.). The enriched genes in the Ubl pathway included multiple members of the ubiquitin-proteasome system, including HECTD1 and RNF8 E3 ubiquitin-protein ligases that were shared between the cerebellum and cortex of SCA1 patients (Figures 2(e) and 2(f)).

**Figure 3:**
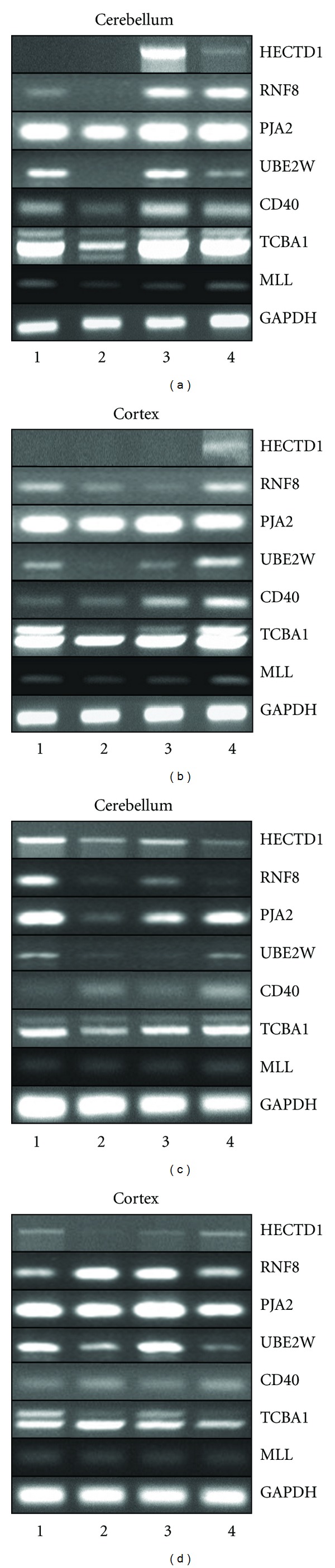
Expression of HECTD1 and RNF8 in the cerebellum and cortex of SCA1 and Alzheimer patients. (a, c) RT-PCR analysis of HECTD1, ring finger protein 8 (RNF8), praja ring finger 2 (PJA2), ubiquitin-conjugating enzyme E2W (UBE2W), CD40, TCBA1, MLL, and GAPDH expression in the cerebellum, involving two independent sets of samples derived from (1) young, (2) old healthy controls, (3) SCA1, and (4) Alzheimer patients. (b, d) RT-PCR analysis of HECTD1, RNF8, PJA2, UBE2W, CD40, TCBA1, MLL, and GAPDH expression in the cortex, involving two independent sets of samples derived from (1) young, (2) old healthy controls, (3) SCA1, and (4) Alzheimer individuals.

The differential regulation of ncRNA expression and target gene enrichment in the cortex and cerebellum of SCA1 subjects suggested that the expression levels of Ubl pathway genes might be affected as well, and, as such, they may play a role in the progressive degeneration of cerebellar neurons [17]. Ubiquitin-positive nuclear accumulations that contained ATXN1 (GC06M016299) mutant protein, the SCA1 disease-causing gene, have been reported [18]. Subsequently we scanned HECTD1 (C14M031569), RNF8 (GC06P037321), UBE2W (GC08M074702), and PJA2 (GC05M108698) expression by RT-PCR analysis and found that mRNA levels of HECTD1, RNF8, and UBE2W were markedly increased in SCA1 brain samples that appeared to be restricted to the cerebellum, as compared to the relatively protected cortical neurons (Figures 3(a), 3(b), 3(c), and 3(d)). Further, we found HECTD1 and RNF8 mRNA induction in the cortex of Alzheimer patients (Figures 3(b) and 3(d)). In contrast, expression levels of TCBA1, also known as NKIAN1 (GC06P124125), MLL (GC11P118341), and CD40 (GC20P044746) that were found to be overrepresented in the ncRNA target gene screen remained stable (Figures 3(a)–3(d)). However, it is worth noting the quantitative difference of the increased mRNA levels of HECTD1 and RNF8 between the two SCA1 subjects, which might reflect the individual predisposition to the cytotoxic effect of ATXN1.

**Figure 4:**
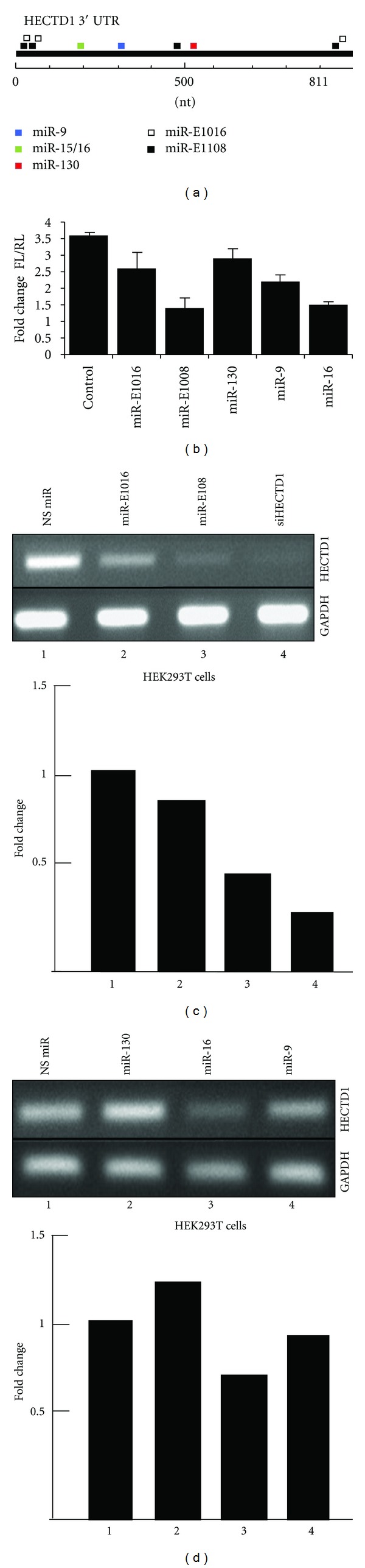
miRNAs and ncRNAs reduce the expression of HECTD1 in HEK29 3T cells. (a) Schematic diagram of HECTD1 3′UTR depicting the location of relevant miRNA response elements and miR-E1108 and miR-E1016 binding sites. (b) Luciferase assay using HECTD1 3′UTR reporter and miRNA mimics. (c) RT-PCR analysis of HECTD1 and GAPDH expression after transfection with miR-E1108, miR-E1016, and siHECTD1. Relative fold changes in HECTD1 protein levels after transfection with miRNA mimics are shown in lower panel. (d) RT-PCR analysis of HECTD1 and GAPDH after transfection with miR-16, miR-9, and miR-130. Relative fold changes in HECTD1 protein levels after transfection with miRNA mimics are shown in lower panel.

The induction of these particular ncRNAs to target genes involved in protein ubiquitination is probably intended to compensate for the loss of transcriptional control due to the accumulation of mutant ATXN1, but whether it serves to delay or accelerate the disease progression remains unclear at this point. Indeed, human HECTD1 has a long 3′UTR (811 bp) that contains a number of conserved miRNA response elements, including multiple binding sites in a favorable context for miR-E1108 and miR-1016 (Table 1). In line with our hypothesis, miR-E1108 and miR-E1016 were identified as the main HECTD1 targeting ncRNA, with increased expression in the cerebellum and cortex of SCA1 patients (Figure 4(a)). Moreover, the ncRNA suppressive pressure on HECTD1 appears to be reduced in the cerebellum of SCA1 patients, while the number of ncRNAs targeting HECTD1 is increased in the cortex (Supplementary Table 3). Of the predicted miRNA sites, we chose five potential candidates on the basis of the cognate element positions within HECTD1 3′UTR and their neuronal expression. To validate the role of the selected ncRNAs and miRNAs in modulating HECTD1 expression levels, we transfected HEK293T and HeLa cells with a vector containing the entire HECTD1 3′UTR region ligated downstream of the luciferase reporter gene and carried out dual luciferase assays in the presence of miR-130, miR-16, miR-9, and miR-E1108 and miR-E1016. In the context of the full-length 3′UTR (pGL3-hHECTD1 3′UTR 1–811), miR-E1108, miR-16, and miR-9 markedly reduced reporter gene expression, whereas the silencing effect of miR-E1016 was weaker and miR-130 was ineffective, as compared to the pGL3 control (Figure 4(b) and Supplementary Figure 1). Thus, miR-E1108 and miR-E1016 appeared to function as miRNAs based on the functional data and the RNA structure of the precursor genes, although more detailed analysis is required to classify them as genuine miRNAs (Supplementary Figure 2) [19]. As a second approach, we transfected HEK293T cells with synthetic miRNA mimics. A small but significant decrease in HECTD1 mRNA levels was consistently observed, providing additional evidence that miRNA binding sites act as repressor elements (Figures 4(c) and 4(d)). In conclusion, these data indicate that these newly identified ncRNAs act as miRNAs and have the ability to bind directly to the HECTD1 3′UTR and suppress its expression.

**Figure 5:**
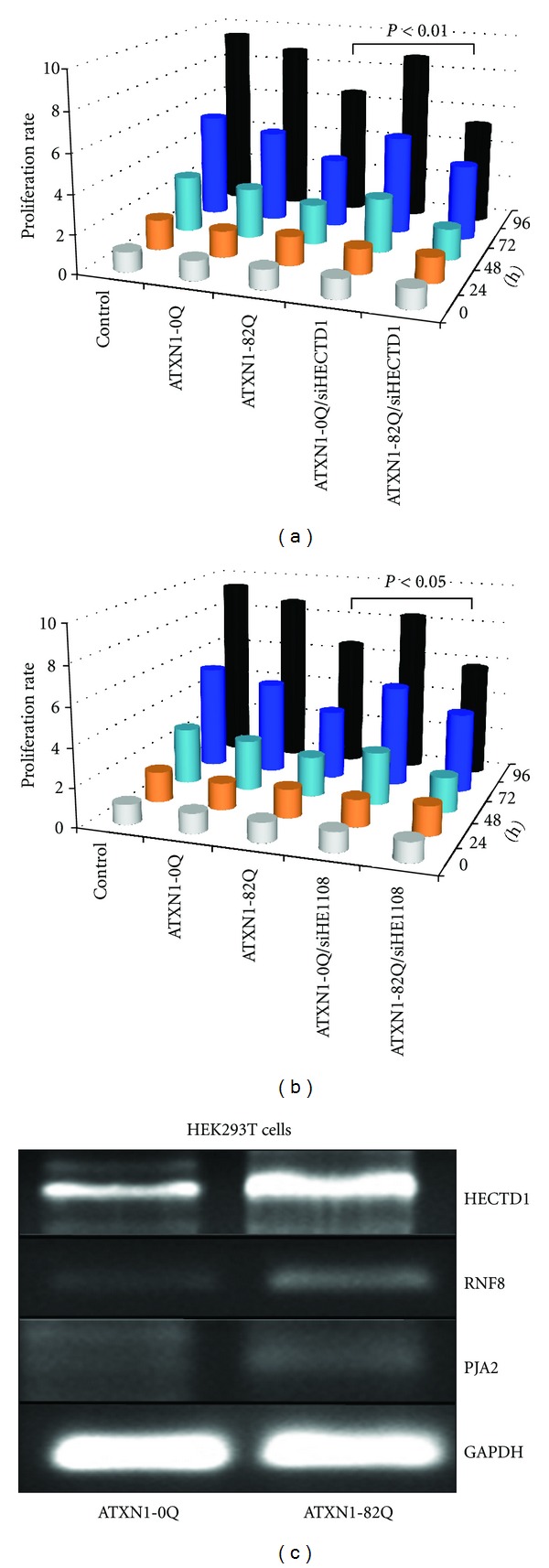
ncRNA-mediated inhibition of HECTD1 causes more severe cytotoxicity in HEK293T cells. (a) Inhibition of HECTD1 by specific siRNA mixture increased significantly the lethality of cells transfected with the polyQ-expanded ATXN1 (ATXN182Q) vector as compared to wild-type ATXN1. (b) Inhibition of HECTD1 by miR-E1108 increased significantly the lethality of cells transfected with the polyQ-expanded ATXN1. Data are shown as mean values for clarity. Statistical analysis was performed using ANOVA. (c) Exogenous overexpression of polyQ-expanded ATXN1 leads to increased mRNA levels of HECTD1, RNF8, and PJA2.

To investigate whether the inhibition of HECTD1 can modulate the cytotoxicity of the polyQ-expanded ATXN1, we transfected HEK293T cells with polyQ-expanded ATXN1 expression vector containing the wild-type and mutated human ATXN1 (GFP-hATXN10Q and GFP-hATXN182Q) and carried out a cell-based assay of mutant ATXN1 toxicity. We used HEK293T cells because the molecular mechanisms that contribute to SCA1 pathogenesis in the cerebellum have been reproduced in this cell line and exogenous expression of polyQ-expanded ATXN1 induces cell death [4, 20]. We tested whether the inhibition of endogenous HECTD1 by miR-E1108 and HECTD1-specific siRNAs affected cytotoxicity of the polyQ-expanded ATXN1 (hATXN182Q) (Figures 5(a) and 5(b)). The inhibition of HECTD1 by siRNAs and miR-E1108 reduced significantly the cell viability of ATXN182Q-transfected cells, 72 h and 96 h after transfection, as compared to cotransfection with hATXN10Q (Figures 5(a) and 5(b)). To ascertain that the effect of ATXN182Q on cell viability is augmented by HECTD1, we monitored the expression levels of HECTD1, RNF8, PJA2, and UBE2W expression in HEK293T cells. The results shown in Figure 5(c) revealed that the mRNA levels of HECTD1 and RNF8 were increased in the cells transfected with ATXN182Q expression vector. Collectively, these data suggest that inhibition of miRNA and ncRNA-mediated posttranscriptional regulation of HECTD1 enhances ATXN1 cytotoxicity in the HEK293T cell model.

Our results reveal the dysregulation of an essential protein processing network in brain compartments specifically affected by particular neurodegenerative process. It is conceivable that the increased ncRNA levels are the result of aberrant RNA processing. Thus, the inhibition of HECTD1 and potentially other components of Ubl proteasomal pathway by ncRNAs can exacerbate the neuron damage and account for the gradual progression of the neurodegenerative process. 

The accumulation of proteins is a recurring event in many neurodegenerative diseases, including SCA1 and Alzheimer's disease [21, 22]. It has been suggested that protein accumulation may result from a dysfunction in the ubiquitin proteasome system (UPS). Indeed, there is mounting genetic and biochemical evidence of an involvement of the ubiquitin proteasome system in SCA1 [3, 23]. HECTD1 and RNF8 are E3 ubiquitin-protein ligases, which accept ubiquitin from an E2 ubiquitin-conjugating enzyme in the form of a thioester and then directly transfer the ubiquitin to one or more lysine residues in the targeted substrates. The HECT family of protein ligases ubiquitinate proteins for degradation by the 26S proteasome protein complex and have nonredundant functions in regulating specific signaling cascades [24, 25]. As such, deregulation of HECT ligases and the proteins that regulate their function can lead to a number of human diseases. Neurons appeared to be especially sensitive to malfunctions of the protein ubiquitination pathway, and HECTD1 upregulation in postmitotic neurons appears to be characteristic for the degenerating neurons. 

The HECTD1 gene is located on chromosome 14q12 in a region that has recently been mapped to be associated with autosomal recessive spastic paraplegia (SPG32) [26], a condition characterized by cerebellar and cortical atrophy, among other clinical features linked to neurodegeneration. In addition, a search of the genomic variants database revealed that individuals with deletion of one copy of the HECTD1 gene are present in the population, which suggests that lower HECTD1 expression levels might be a risk factor for developing age-related neurodegenerative disorders.

## 4. Concluding Remarks

Dysregulation of the UPS (ubiquitin-proteasome system) has been implicated in a wide range of pathologies including cancer, neurodegeneration, and viral infection. Inhibiting the proteasome has been shown to be an effective therapeutic strategy in humans, although toxicity with regard to this target remains high. E3s (Ub-protein ligases) represent an alternative attractive therapeutic target in the UPS. In this study we used genomewide miRNA and ncRNA profiling as a screening strategy for identifying deregulated gene networks in SCA1 cerebellum. We detected E3 ligase genes that are regulated by the miRNA machinery and appear to play an essential protein-processing role in neuronal cells. Our finding underscores the importance of small ncRNAs for the gene regulation and protein processing in the aging brain and may provide opportunities for intervention in neurodegenerative diseases as an alternative to E3 ligase inhibitors.

## Supplementary Material

Supplementary Figure 1. Luciferase assay of HECTD13ÚTR/miR-E1016 and miR-E1108 cotransfections in HeLa cells.
Supplementary Figure 2. miRNA E1108 and miR-E1016 primary genes and processed sequences.
Supplementary Table 1. List of novel small non-coding RNAs (miRNAs) with elevated expression levels in the aging human cortex and cerebellum of healthy individuals and SCA1 patients.
Supplementary Table 2. List of novel small non-coding RNAs (miRNAs) with elevated expression levels in the aging human cortex and cerebellum of healthy individuals.
Supplementary Table 3. Quantitative comparison of the novel upregulated ncRNAs that target ubiquitin-protein processing genes in the cortex and cerebellum of healthy aged individuals and SCA1 patients.
Supplementary Table 4. List of primers used for RT-PCR analysis and HECTD 3'UTR cloning.
Supplementary Table 5. Summary of subject biological parameters and clinical history.
Supplementary Table 6. Gene ontology (GO) annotation of ncRNA target genes in SCA1 cerebellum.
Supplementary Table 7. GO annotation of ncRNA target genes in SCA 1 frontal cortex.
Supplementary Table 8. Gene ontology (GO) annotation of ncRNA target genes in the cerebellum of healthy aged individuals.
Supplementary Table 9. Gene ontology (GO) annotation of ncRNA target genes in the frontal cortex of healthy aged individuals.

## Figures and Tables

**Table 1:** Sequence and the matching sites of miR-E1016 and miR-E1108 within HECTD1 3′UTR.

miRNA	Target gene	Gene position	Start site	3′UTR target sequence	Match sequence	miRNA Sequence
E1016	HECTD1	(Ch14:30667550-30668553) (−)	765	aagagacaguuuucugcauugguu	∣∣∣ ∣∣ ∣ ∣∣∣∣∣∣∣∣	ccgucuuaguaauguaguaaccaa
	HECTD1	(Ch14:30670304-30670441) (−)	32	gacaaaguguuguugccauuggua	∣ ∣∣ ∣*․*∣ ∣ *․․* ∣∣∣∣∣∣∣	ccgucuuaguaauguaguaaccaa
	HECTD1	(Ch14:30673766-30674154) (−)	67	guaaguuauuuuagaacauugguu	∣ ∣∣ *․* ∣∣∣ ∣ ∣∣∣∣∣∣∣∣	ccgucuuaguaauguaguaaccaa

E1108	HECTD1	(Ch14:30639237-30640029) (−)	762	cauaguuagagucaacauuuu	∣∣*․․* ∣∣ ∣∣∣∣∣∣∣∣	cacucgggcagauuuguaaaa
	HECTD1	(Ch14:30645593-30646219) (−)	472	auaacaauauguuaacauuug	∣ ∣ *․* ∣ ∣ ∣∣∣∣∣∣∣	cacucgggcagauuuguaaaa
	HECTD1	(Ch14:30655178-30655341) (−)	64	aagaaaaaggauuaacauuuu	∣∣ ∣∣ ∣∣∣∣∣∣∣∣	cacucgggcagauuuguaaaa
	HECTD1	(Ch14:30672189-30672373) (−)	40	gguaaauuuauuuaacauuuu	∣∣*․․* *․*∣ ∣∣∣∣∣∣∣∣	cacucgggcagauuuguaaaa
	HECTD1	(Ch14:30687845-30687983) (−)	56	uuuguauauuauaaacauuua	∣ *․․*∣ ∣∣∣∣∣∣∣∣∣	cacucgggcagauuuguaaaa
