# Supplementary material for: Functional Annotation of Small Noncoding RNAs Target Genes Provides Evidence for a Deregulated Ubiquitin-Proteasome Pathway in Spinocerebellar Ataxia Type 1
Source: J Nucleic Acids. 2012 Oct 3;2012:672536. doi: 10.1155/2012/672536 (PMC3471453; doi:10.1155/2012/672536)
Supplement: Supplementary file 1 — Supplementary Figure 1. Luciferase assay of HECTD13ÚTR/miR-E1016 and miR-E1108 cotransfections in HeLa cells. Supplementary Figure 2. miRNA E1108 and miR-E1016 primary genes and processed sequences. Supplementary Table 1. List of novel small non-coding RNAs (miRNAs) with elevated expression levels in the aging human cortex and cerebellum of healthy individuals and SCA1 patients. Supplementary Table 2. List of novel small non-coding RNAs (miRNAs) with elevated expression levels in the aging human cortex and cerebellum of healthy individuals. Supplementary Table 3. Quantitative comparison of the novel upregulated ncRNAs that target ubiquitin-protein processing genes in the cortex and cerebellum of healthy aged individuals and SCA1 patients. Supplementary Table 4. List of primers used for RT-PCR analysis and HECTD 3'UTR cloning. Supplementary Table 5. Summary of subject biological parameters and clinical history. Supplementary Table 6. Gene ontology (GO) annotation of ncRNA target genes in SCA1 cerebellum. Supplementary Table 7. GO annotation of ncRNA target genes in SCA 1 frontal cortex. Supplementary Table 8. Gene ontology (GO) annotation of ncRNA target genes in the cerebellum of healthy aged individuals. Supplementary Table 9. Gene ontology (GO) annotation of ncRNA target genes in the frontal cortex of healthy aged individuals. [file 672536.f1.pdf]

Supplementary Figure 1. Persengiev et al.

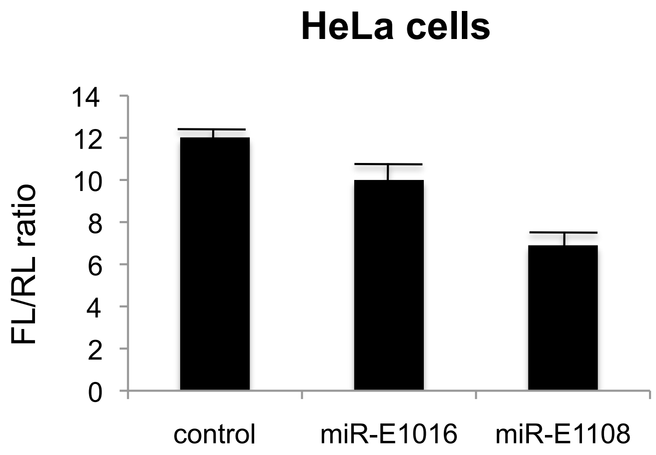

Supplementary Figure 2. Persengiev et al.

miR-E1016 mature sequence 5’-AACCAAUGAUGUAAUGAUUCUGCC-3’

miR-E1108 mature sequence 5’-AAAAUGUUUAGACGGGCUCAC-3

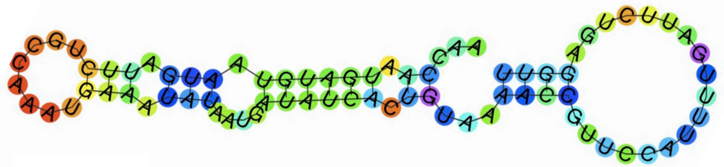

miR-E1016 SNORD47 precursor gene

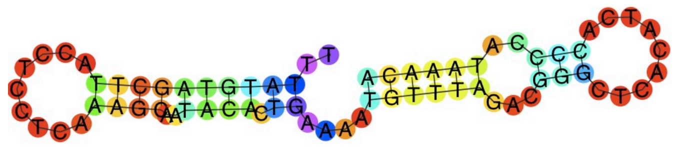

miR-E1108 MT-TF tRNA phenilalanine precursor gene

Supplementary Table 1. List of novel small non-coding RNAs (miRNAs) with elevated expression levels in the aging human cortex and cerebellum of SCA1 patients.

**Claster 1 and 2**	**SCA1 cerebellum**
**miRNA ID**	**miRNA sequence**
hsa-miRPlus-E1088	UGCAGAGUGGGGUUUUGCAGUCCUU
hsa-miRPlus-E1110	GGAAAUUUGAGACCAGCAAGU
hsa-miRPlus-E1141	GCGUAAAGAGUGUUUUAGAUCACCC
hsa-miRPlus-E1202	GAUUAGGGUGCUUAGCUGUUAACU
hsa-miRPlus-E1245	GAGGAAGGUGGGGAUGC
hsa-miRPlus-E1016	AACCAAUGAUGUAAUGAUUCUGCC
hsa-miRPlus-E1108	AAAAUGUUUAGACGGGCUCAC
hsa-miRPlus-E1170	GUUUAGACGGGCUCACAU
hsa-miRPlus-F1218	UGUGUUGAUGUGAAUGGA
hsa-miRPlus-F1232	GUGUGUGAGUGUGCAAGA
**Claster 1 and 2**	**SCA1 frontal cortex**
**miRNA ID**	**miRNA sequence**
hsa-miRPlus-E1153	AUGAGGUGGCAAGAAAUGGGCU
hsa-miRPlus-E1172	AAGAAUGACCGCUGAAGAACGU
hsa-miRPlus-E1030	AAUCUGAGUGAGAGGUUAGUUGCU
hsa-miRPlus-E1076	GCAGAUAAACUCAUGCCAGAGAACU
hsa-miRPlus-E1258	GGGGAUGUAGCUCAGUGGU
hsa-miRPlus-A1065	UCGACCGGACCUCGACCGGCU
hsa-miRPlus-E1031	AUUAAAAAUUUCGGUUGGG
hsa-miRPlus-E1033	GAGCCUGGUGAUAGCUGG
hsa-miRPlus-E1065	AUUGGUCGUGGUUGUAGU
hsa-miRPlus-E1038	GCAUGAGUGGUUCAGUGGU
hsa-miRPlus-E1060	UUGCUGUGAUGACUAUCUUAGGAC
hsa-miRPlus-E1252	GCAUUGGUGGUUCAGUGGU
hsa-miRPlus-E1103	UCCCUGGUGGUCUAGU
hsa-miRPlus-E1047	GCUGAGUGAAGCAUUGGACUGU
hsa-miRPlus-E1136	UUCUAAGCCAGUUUCUGUCUGAU
hsa-miRPlus-E1247	GAUGCCUGGGAGUUGCGAUCU
hsa-miRPlus-E1078	GACUCUUAGCGGUGGAUC
hsa-miRPlus-E1088	UGCAGAGUGGGGUUUUGCAGUCCUU
hsa-miRPlus-E1108	AAAAUGUUUAGACGGGCUCAC
hsa-miRPlus-E1016	AACCAAUGAUGUAAUGAUUCUGCC
hsa-miRPlus-E1100	AGUAAGGUCAGCUAAAUAAGCU
hsa-miRPlus-E1141	GCGUAAAGAGUGUUUUAGAUCACCC
hsa-miRPlus-E1170	GUUUAGACGGGCUCACAU
hsa-miRPlus-E1202	GAUUAGGGUGCUUAGCUGUUAACU
hsa-miRPlus-E1253	GCGUUGGUGGUAUAGUGGU
hsa-miRPlus-F1195	GAAGCCAUGAGGUGAGAA

Supplementary Table 2. List of novel small non-coding RNAs (miRNAs) with elevated expression levels in the aging human cortex and cerebellum of healthy individuals.

**Claster 1**	**Human cerebellum**
**miRNA ID**	**miRNA sequence**
hsa-miRPlus-E1170	GUUUAGACGGGCUCACAU
hsa-miRPlus-A1072	CAGAGAGGACCACUAUGGCGGG
hsa-miRPlus-F1231	UGAUGACACUGGCAAUUG
hsa-miRPlus-E1236	CGGGGAUCGCCGAGGGCCGGUCGGCCGCC
hsa-miRPlus-F1078	GAGAAGCUGGCGUUCGGAG
hsa-miRPlus-F1225	AGGGGGAAAGGAGGAGGA
hsa-miRPlus-E1258	GGGGAUGUAGCUCAGUGGU
hsa-miRPlus-E1209	UCUCUCCAGGUGACAGAAAGGGCU
hsa-miRPlus-E1053	GCCGCGAGUGGGAGCGGGAGCG
hsa-miRPlus-E1053	GCCGCGAGUGGGAGCGGGAGCG
hsa-miRPlus-E1180	GGGAUGCCAGGCAAGUGAGCAGGUC
hsa-miRPlus-E1072	AUUUGGGCAGGUUGAAAGAAUUU
hsa-miRPlus-F1080	GCGGAAGGCGGAGCGGCGGA
hsa-miRPlus-E1026	AAUUGUGAGGCUUGAGUGU
hsa-miRPlus-F1099	AUUGGAAUCCCCGCUAGAGCGUG
hsa-miRPlus-E1016	AACCAAUGAUGUAAUGAUUCUGCC
hsa-miRPlus-C1110	UUUGAUAAGCUGACAUGGGACA
hsa-miRPlus-E1076	GCAGAUAAACUCAUGCCAGAGAACU
hsa-miRPlus-F1166	AUGGUUGACCAGAGAGCA
hsa-miRPlus-E1200	AGUUUUGUGUGUUGGCU
hsa-miRPlus-F1086	GGGAAGAGAGAGAAUGGAAC
hsa-miRPlus-E1097	AUUUUGGGUGGAAGAGGCAU
hsa-miRPlus-D1058	AAAGCAUGCUCCAGUGGCGC
hsa-miRPlus-F1056	GAAAUCGCUUCCCUUUGGA
hsa-miRPlus-F1205	GUCGCUGGGAAGGAAGGA
hsa-miRPlus-F1066	UGGGGGAGGAGGAAGGAC
hsa-miRPlus-E1203	CCUUCGAGGCGGCUGAGACCC
hsa-miRPlus-E1151	CUGAUGGAGAGAAGAAGGCAU
hsa-miRPlus-E1161	AGGUGAGGGGCAGGACCUGAAGGU
hsa-miRPlus-E1075	AUGGCAGUUGGAGAGAAAGAAC
hsa-miRPlus-E1011	GAGGCUGAGGCGAGAGGU
hsa-miRPlus-E1110	GGAAAUUUGAGACCAGCAAGU
hsa-miRPlus-E1116	GGUUUAGUGAGCAGAGUU
hsa-miRPlus-E1175	GUCGAGGUCUUUGGUGGGUUG
hsa-miRPlus-F1021	GGGAGGACAAAGGACUGGC
hsa-miRPlus-E1216	GAGAGUCUCAGGAAAGAAAGGUC
hsa-miRPlus-F1050	GGGAAAAGGAAGGGGGAGGA
hsa-miRPlus-F1187	AGAGUCGAGAGUGGGAGAA
hsa-miRPlus-F1232	GUGUGUGAGUGUGCAAGA
**Claster 1**	**Human frontal cortex**
**miRNA ID**	**miRNA sequence**
hsa-miRPlus-F1136	AGUGGAGGAGGAGGCGCGG
hsa-miRPlus-F1086	GGGAAGAGAGAGAAUGGAAC
hsa-miRPlus-F1078	GAGAAGCUGGCGUUCGGAG
hsa-miRPlus-E1216	GAGAGUCUCAGGAAAGAAAGGUC
hsa-miRPlus-F1032	GUUCUUGGACCAGGACGC
hsa-miRPlus-C1110	UUUGAUAAGCUGACAUGGGACA
hsa-miRPlus-E1161	AGGUGAGGGGCAGGACCUGAAGGU
hsa-miRPlus-F1050	GGGAAAAGGAAGGGGGAGGA
hsa-miRPlus-E1053	GCCGCGAGUGGGAGCGGGAGCG
hsa-miRPlus-E1026	AAUUGUGAGGCUUGAGUGU
hsa-miRPlus-E1203	CCUUCGAGGCGGCUGAGACCC
hsa-miRPlus-E1129	AUGUGAGAGCAGCAGAGGCGGU
hsa-miRPlus-F1099	AUUGGAAUCCCCGCUAGAGCGUG
hsa-miRPlus-F1205	GUCGCUGGGAAGGAAGGA
hsa-miRPlus-E1075	AUGGCAGUUGGAGAGAAAGAAC
hsa-miRPlus-E1113	AGAUGAGCUGAAGGG
hsa-miRPlus-F1216	GGAGAGGGAAAAGAAAAG
hsa-miRPlus-F1225	AGGGGGAAAGGAGGAGGA
hsa-miRPlus-F1091	UGAGGCCGAGAAGGCAACCG
hsa-miRPlus-E1097	AUUUUGGGUGGAAGAGGCAU

Supplemetary Table 3. Quantitative comparison of the novel upregulated ncRNAs that target ubiquitin-protein processing genes in the cortex and cerebellum of healthy aged individuals and SCA1 patients.

Healthy controls

		Cortex		Cerebellum	
**Target genes**	**Function**	**# of miRNAs**	**# of sites**	**# of miRNAs**	**# of sites**
**HECTD1**	E3 ubiquitin-protein ligase HECTD1 (HECT domain-containing protein 1)(E3 ligase for inhibin receptor)	ND	ND	5	16
**RNF8**	ring finger protein 8	ND	ND	ND	ND
**UBE2W**	ubiquitin-conjugating enzyme E2W (putative)	5	5	5	5
**PJA2**	praja ring finger 2	1	1	5	5

SCA1 patients

		Cortex		Cerebellum	
**Target genes**	**Function**	**# of miRNAs**	**# of sites**	**# of miRNAs**	**# of sites**
**HECTD1**	E3 ubiquitin-protein ligase HECTD1 (HECT domain-containing protein 1)(E3 ligase for inhibin receptor)	4	14	2	8
**RNF8**	ring finger protein 8	ND	ND	1	2
**UBE2W**	ubiquitin-conjugating enzyme E2W (putative)	2	6	1	2
**PJA2**	praja ring finger 2	6	6	3	4

Supplementary Table 4. List of primers used for RT-PCR analysis and HECTD 3'UTR cloning

**RT-PCR primers**	
HECTD1-F	CTGGAGCAGGGAGCCGACCT
HECTD2-R	CTGGCAGAGCCCCACTTGCC
RNF8-F	GCCCCATTTGTCGGAAGGACAT
RNF8-R	AGCAGCGGAGTCACGGCTAGA
UBE2W-F	ATGGCGTCAATGCAGACCACAGG
UBE2W-R	GCGCCCAGAATGCACACGAG
PJA2-F	GTGGCGGGTGGAGGTTCGAG
PJA2-R	TGGTCCATTGCTGCTGGCTCC
CD40-F	CACACTGCCACCAGCACAA
CD40-R	GCCTTCTTCACAGGTGCAGAT
MLL-F	GCAGGGAAGAAAGGTGAAGACA
MLL-R	AATGATCCGCGAGGAGATAGC
TCBA1-F	GGGCCCCAGAAGACCATCGC
TCBA1-R	CCGGCAACCGTTGGGCATCA
**HECTD1 3'-UTR cloning primers**	
HECTD1–F	GCTTTGAAGTGCAATGGGAG
HECTD1-R	ACGCTGCAAACATTTTAATGATT

Supplementary Table 5.

Sample	Sex	Age	Cause of death
Human (*Homo sapiens*)	male	17 years	traffic accident
Human (*Homo sapiens*)	male	19 years	traffic accident
Human (*Homo sapiens*)	male	61 years	traffic accident
Human (*Homo sapiens*)	male	62 years	traffic accident
Human (*Homo sapiens*)	male	47 years	SCA1
Human (*Homo sapiens*)	male	65 years	SCA1
Human (*Homo sapiens*)	male	93 years	Alzheimer
Human (*Homo sapiens*)	male	76 years	Alzheimer

Supplementary Table 6. GO annotation of ncRNA target genes in SCA1 cerebellum.

Annotation Cluster 1 Enrichment Score: 2.43044418088753

Category	Term	Count	%	PValue	List Total	Pop Hits	Pop Total	Fold Enrichment	Bonferroni	Benjamini	FDR
GOTERM_BP_FAT	GO:0045944~positive regulation of transcription from RNA polymerase II promoter	13	0.67708333	9.27E-04	152	371	13528	3.118598383	0.72625617	0.47679466	1.51602084
GOTERM_BP_FAT	GO:0045893~positive regulation of transcription, DNA-dependent	13	0.67708333	0.00719483	152	477	13528	2.42557652	0.9999584	0.53973948	11.2142816
GOTERM_BP_FAT	GO:0051254~positive regulation of RNA metabolic process	13	0.67708333	0.00766631	152	481	13528	2.405405405	0.99997858	0.51165811	11.906268

Annotation Cluster 2 Enrichment Score: 2.2414481844226324

Category	Term	Count	%	PValue	List Total	Pop Hits	Pop Total	Fold Enrichment	Bonferroni	Benjamini	FDR
UP_SEQ_FEATURE	domain:Ig-like C2-type 3	7	0.36458333	0.00135203	190	122	19113	5.771829163	0.77936277	0.26084459	2.14441794
UP_SEQ_FEATURE	domain:Ig-like C2-type 1	7	0.36458333	0.01169318	190	190	19113	3.706121884	0.99999803	0.63601188	17.1764247
UP_SEQ_FEATURE	domain:Ig-like C2-type 2	7	0.36458333	0.01193267	190	191	19113	3.686718104	0.9999985	0.6162572	17.4974215

Annotation Cluster 3 Enrichment Score: 1.9692985529641533

Category	Term	Count	%	PValue	List Total	Pop Hits	Pop Total	Fold Enrichment	Bonferroni	Benjamini	FDR
GOTERM_BP_FAT	GO:0010975~regulation of neuron projection development	6	0.3125	0.00109511	152	70	13528	7.628571429	0.78361765	0.39964618	1.78870544
GOTERM_BP_FAT	GO:0031344~regulation of cell projection organization	6	0.3125	0.00317191	152	89	13528	6	0.98818279	0.46954935	5.0986162
GOTERM_BP_FAT	GO:0045664~regulation of neuron differentiation	6	0.3125	0.01666079	152	133	13528	4.015037594	1	0.49858897	24.1759463
GOTERM_BP_FAT	GO:0050767~regulation of neurogenesis	6	0.3125	0.03841873	152	166	13528	3.21686747	1	0.6439291	47.5507913
GOTERM_BP_FAT	GO:0051960~regulation of nervous system development	6	0.3125	0.0640457	152	192	13528	2.78125	1	0.74328453	66.3878905

Annotation Cluster 4 Enrichment Score: 1.8804120872449892

Category	Term	Count	%	PValue	List Total	Pop Hits	Pop Total	Fold Enrichment	Bonferroni	Benjamini	FDR
GOTERM_BP_FAT	GO:0007409~axonogenesis	8	0.41666667	0.00593204	152	193	13528	3.689119171	0.99975438	0.49974887	9.33563495
GOTERM_BP_FAT	GO:0048667~cell morphogenesis involved in neuron differentiation	8	0.41666667	0.00902444	152	209	13528	3.406698565	0.99999684	0.50518761	13.8714151
GOTERM_BP_FAT	GO:0048812~neuron projection morphogenesis	8	0.41666667	0.00995438	152	213	13528	3.342723005	0.99999915	0.48599439	15.1931453
GOTERM_BP_FAT	GO:0000904~cell morphogenesis involved in differentiation	8	0.41666667	0.01967629	152	244	13528	2.918032787	1	0.50926024	27.9165998
GOTERM_BP_FAT	GO:0048858~cell projection morphogenesis	8	0.41666667	0.02007176	152	245	13528	2.906122449	1	0.50743903	28.3941043
GOTERM_BP_FAT	GO:0032990~cell part morphogenesis	8	0.41666667	0.02479476	152	256	13528	2.78125	1	0.5258707	33.8719555

Annotation Cluster 5 Enrichment Score: 1.8754286740079529

Category	Term	Count	%	PValue	List Total	Pop Hits	Pop Total	Fold Enrichment	Bonferroni	Benjamini	FDR
GOTERM_BP_FAT	GO:0050770~regulation of axonogenesis	5	0.26041667	0.00372411	152	57	13528	7.807017544	0.99455081	0.47875547	5.96088568
GOTERM_BP_FAT	GO:0010769~regulation of cell morphogenesis involved in differentiation	5	0.26041667	0.01075247	152	77	13528	5.779220779	0.99999972	0.46702077	16.3122759
GOTERM_BP_FAT	GO:0022604~regulation of cell morphogenesis	5	0.26041667	0.05904505	152	131	13528	3.396946565	1	0.74064221	63.3042387

Annotation Cluster 6 Enrichment Score: 1.7880573881131052

Category	Term	Count	%	PValue	List Total	Pop Hits	Pop Total	Fold Enrichment	Bonferroni	Benjamini	FDR
UP_SEQ_FEATURE	zinc finger region:NR C4-type	4	0.20833333	0.00995752	190	45	19113	8.941754386	0.99998603	0.60604739	14.8148951
UP_SEQ_FEATURE	DNA-binding region:Nuclear receptor	4	0.20833333	0.00995752	190	45	19113	8.941754386	0.99998603	0.60604739	14.8148951
INTERPRO	IPR001628:Zinc finger, nuclear hormone receptor-type	4	0.20833333	0.01380119	183	46	16659	7.915894512	0.99640572	0.84681935	17.6499357
GOTERM_MF_FAT	GO:0003707~steroid hormone receptor activity	4	0.20833333	0.01429894	136	49	12983	7.792917167	0.99099076	0.40742529	17.7152048
INTERPRO	IPR001723:Steroid hormone receptor	4	0.20833333	0.014626	183	47	16659	7.747471224	0.99743879	0.77503691	18.6071202
INTERPRO	IPR008946:Nuclear hormone receptor, ligand-binding	4	0.20833333	0.01547818	183	48	16659	7.586065574	0.99819588	0.71734745	19.5852167
INTERPRO	IPR000536:Nuclear hormone receptor, ligand-binding, core	4	0.20833333	0.01547818	183	48	16659	7.586065574	0.99819588	0.71734745	19.5852167
INTERPRO	IPR013088:Zinc finger, NHR/GATA-type	4	0.20833333	0.01819985	183	51	16659	7.139826422	0.99941203	0.65447543	22.6364298
GOTERM_MF_FAT	GO:0004879~ligand-dependent nuclear receptor activity	4	0.20833333	0.02238972	136	58	12983	6.5836714	0.99939158	0.52310774	26.4032002
SMART	SM00399:ZnF_C4	4	0.20833333	0.02621757	128	46	9079	6.167798913	0.94619622	0.76804358	25.9303896
SMART	SM00430:HOLI	4	0.20833333	0.02928002	128	48	9079	5.910807292	0.9619516	0.55834402	28.5200189

Annotation Cluster 7 Enrichment Score: 1.7552264184713415

Category	Term	Count	%	PValue	List Total	Pop Hits	Pop Total	Fold Enrichment	Bonferroni	Benjamini	FDR
SP_PIR_KEYWORDS	ubl conjugation pathway	14	0.72916667	0.00161633	190	509	19235	2.784510392	0.3140219	0.05242029	2.05672421
GOTERM_BP_FAT	GO:0019941~modification-dependent protein catabolic process	14	0.72916667	0.01204264	152	574	13528	2.170731707	0.99999996	0.47846986	18.0921253
GOTERM_BP_FAT	GO:0043632~modification-dependent macromolecule catabolic process	14	0.72916667	0.01204264	152	574	13528	2.170731707	0.99999996	0.47846986	18.0921253
GOTERM_BP_FAT	GO:0051603~proteolysis involved in cellular protein catabolic process	14	0.72916667	0.01683821	152	600	13528	2.076666667	1	0.49227122	24.4009919
GOTERM_BP_FAT	GO:0044257~cellular protein catabolic process	14	0.72916667	0.01747377	152	603	13528	2.066334992	1	0.48602732	25.2019934
GOTERM_BP_FAT	GO:0030163~protein catabolic process	14	0.72916667	0.02193229	152	622	13528	2.003215434	1	0.52175332	30.6009912

GOTERM_BP_FAT	GO:0044265~cellular macromolecule catabolic process	14	0.72916667	0.06189608	152	725	13528	1.71862069	1	0.74139011	65.0934203
GOTERM_BP_FAT	GO:0009057~macromolecule catabolic process	14	0.72916667	0.09700301	152	781	13528	1.595390525	1	0.82046807	81.3770623
Annotation Cluster 8	Enrichment Score: 1.713745709225738										
Category	Term	Count	%	PValue	List Total	Pop Hits	Pop Total	Fold Enrichment	Bonferroni	Benjamini	FDR
GOTERM_BP_FAT	GO:0010557~positive regulation of macromolecule biosynthetic process	15	0.78125	0.01465974	152	654	13528	2.041284404	1	0.48599612	21.5938865
GOTERM_BP_FAT	GO:0031328~positive regulation of cellular biosynthetic process	15	0.78125	0.02101201	152	685	13528	1.948905109	1	0.51498462	29.5175035
GOTERM_BP_FAT	GO:0009891~positive regulation of biosynthetic process	15	0.78125	0.0234514	152	695	13528	1.920863309	1	0.52926114	32.3553569
Annotation Cluster 9	Enrichment Score: 1.5641019344690972										
Category	Term	Count	%	PValue	List Total	Pop Hits	Pop Total	Fold Enrichment	Bonferroni	Benjamini	FDR
UP_SEQ_FEATURE	domain:Fibronectin type-III 7	3	0.15625	0.02327465	190	24	19113	12.57434211	1	0.78719072	31.4307055
UP_SEQ_FEATURE	domain:Fibronectin type-III 8	3	0.15625	0.02327465	190	24	19113	12.57434211	1	0.78719072	31.4307055
UP_SEQ_FEATURE	domain:Fibronectin type-III 6	3	0.15625	0.03749109	190	31	19113	9.734974533	1	0.8816534	45.7870016
Annotation Cluster 10	Enrichment Score: 1.4905533697538385										
Category	Term	Count	%	PValue	List Total	Pop Hits	Pop Total	Fold Enrichment	Bonferroni	Benjamini	FDR
UP_SEQ_FEATURE	domain:Fibronectin type-III 2	6	0.3125	0.00949836	190	130	19113	4.642834008	0.99997654	0.65562974	14.1796896
UP_SEQ_FEATURE	domain:Fibronectin type-III 1	6	0.3125	0.00979816	190	131	19113	4.607392527	0.99998327	0.63207088	14.5949347
INTERPRO	IPR008957:Fibronectin, type III-like fold	6	0.3125	0.05156792	183	184	16659	2.968460442	1	0.83252099	52.2798449
INTERPRO	IPR003961:Fibronectin, type III	6	0.3125	0.05766686	183	190	16659	2.874719586	1	0.82062084	56.3934276
SMART	SM00060:FN3	6	0.3125	0.12738961	128	190	9079	2.239884868	0.99999969	0.8825009	78.5526376
Annotation Cluster 11	Enrichment Score: 1.2480417974809257										
Category	Term	Count	%	PValue	List Total	Pop Hits	Pop Total	Fold Enrichment	Bonferroni	Benjamini	FDR
INTERPRO	IPR003598:Immunoglobulin subtype 2	8	0.41666667	0.0073819	183	205	16659	3.552499	0.95025071	0.95025071	9.835275
SMART	SM00408:IGc2	8	0.41666667	0.02478424	128	205	9079	2.767987805	0.93674742	0.93674742	24.689225
SP_PIR_KEYWORDS	Immunoglobulin domain	8	0.41666667	0.18067733	190	470	19235	1.723180291	1	0.7872676	92.2703246
INTERPRO	IPR007110:Immunoglobulin-like	8	0.41666667	0.30802457	183	501	16659	1.453617355	1	0.98024381	99.4171679
Annotation Cluster 12	Enrichment Score: 1.2065135547754917										
Category	Term	Count	%	PValue	List Total	Pop Hits	Pop Total	Fold Enrichment	Bonferroni	Benjamini	FDR
INTERPRO	IPR000210:BTB/POZ-like	6	0.3125	0.04662034	183	179	16659	3.051378331	1	0.8833285	48.6811933
INTERPRO	IPR011333:BTB/POZ fold	6	0.3125	0.04863333	183	181	16659	3.017661444	1	0.86723264	50.1747162
SMART	SM00225:BTB	6	0.3125	0.10591317	128	179	9079	2.377531425	0.99999551	0.91481691	71.7722848
Annotation Cluster 13	Enrichment Score: 1.1504405518190628										
Category	Term	Count	%	PValue	List Total	Pop Hits	Pop Total	Fold Enrichment	Bonferroni	Benjamini	FDR
SP_PIR_KEYWORDS	bromodomain	3	0.15625	0.05613987	190	39	19235	7.787449393	0.99999858	0.48987801	52.3963775
INTERPRO	IPR018359:Bromodomain, conserved site	3	0.15625	0.05869602	183	36	16659	7.586065574	1	0.80469777	57.0542003
INTERPRO	IPR001487:Bromodomain	3	0.15625	0.07066421	183	40	16659	6.827459016	1	0.82551402	64.0856529
SMART	SM00297:BROMO	3	0.15625	0.1074381	128	40	9079	5.319726563	0.99999628	0.87553813	72.3114815
Annotation Cluster 14	Enrichment Score: 1.0671594050775701										
Category	Term	Count	%	PValue	List Total	Pop Hits	Pop Total	Fold Enrichment	Bonferroni	Benjamini	FDR
INTERPRO	IPR019787:Zinc finger, PHD-finger	4	0.20833333	0.06617723	183	85	16659	4.283895853	1	0.82326641	61.5853237
INTERPRO	IPR001965:Zinc finger, PHD-type	4	0.20833333	0.07573106	183	90	16659	4.045901639	1	0.82999446	66.727061
INTERPRO	IPR019786:Zinc finger, PHD-type, conserved site	4	0.20833333	0.08173947	183	93	16659	3.915388683	1	0.82214758	69.625254
SMART	SM00249:PHD	4	0.20833333	0.13150671	128	90	9079	3.152430556	0.99999982	0.85610724	79.6685532
Annotation Cluster 15	Enrichment Score: 1.0287501046435323										
Category	Term	Count	%	PValue	List Total	Pop Hits	Pop Total	Fold Enrichment	Bonferroni	Benjamini	FDR
GOTERM_BP_FAT	GO:0050772~positive regulation of axonogenesis	3	0.15625	0.03139374	152	25	13528	10.68	1	0.58260827	40.8693495
GOTERM_BP_FAT	GO:0031346~positive regulation of cell projection organization	3	0.15625	0.09665904	152	47	13528	5.680851064	1	0.82304461	81.2598614
GOTERM_BP_FAT	GO:0050769~positive regulation of neurogenesis	3	0.15625	0.14061109	152	59	13528	4.525423729	1	0.85915801	91.7595891
GOTERM_BP_FAT	GO:0010720~positive regulation of cell development	3	0.15625	0.17984337	152	69	13528	3.869565217	1	0.88317143	96.1834063
Annotation Cluster 16	Enrichment Score: 1.0259061032248193										

Category	Term	Count	%	PValue	List Total	Pop Hits	Pop Total	Fold Enrichment	Bonferroni	Benjamini	FDR
INTERPRO	IPR014778:Myb, DNA-binding	3	0.15625	0.05582555	183	35	16659	7.802810304	1	0.83297502	55.1875996
INTERPRO	IPR001005:SANT, DNA-binding	3	0.15625	0.10007235	183	49	16659	5.573435932	1	0.84380918	77.0844501
SMART	SM00717:SANT	3	0.15625	0.14966994	128	49	9079	4.342633929	0.99999998	0.86215114	83.9873807

Annotation Cluster 17 Enrichment Score: 0.9227144770695282

Category	Term	Count	%	PValue	List Total	Pop Hits	Pop Total	Fold Enrichment	Bonferroni	Benjamini	FDR
GOTERM_BP_FAT	GO:0042981~regulation of apoptosis	14	0.72916667	0.1144046	152	804	13528	1.549751244	1	0.85148412	86.4842233
GOTERM_BP_FAT	GO:0043067~regulation of programmed cell death	14	0.72916667	0.12086536	152	812	13528	1.534482759	1	0.84957507	88.0199251
GOTERM_BP_FAT	GO:0010941~regulation of cell death	14	0.72916667	0.12334207	152	815	13528	1.528834356	1	0.84687779	88.563921

Annotation Cluster 18 Enrichment Score: 0.8181598871914297

Category	Term	Count	%	PValue	List Total	Pop Hits	Pop Total	Fold Enrichment	Bonferroni	Benjamini	FDR
UP_SEQ_FEATURE	repeat:Kelch 5	3	0.15625	0.13871939	190	66	19113	4.572488038	1	0.99027962	90.8618534
UP_SEQ_FEATURE	repeat:Kelch 4	3	0.15625	0.15235952	190	70	19113	4.311203008	1	0.99224108	92.9242144
SP_PIR_KEYWORDS	kelch repeat	3	0.15625	0.15425777	190	71	19235	4.277613047	1	0.76444571	88.3793304
UP_SEQ_FEATURE	repeat:Kelch 3	3	0.15625	0.1558101	190	71	19113	4.250481838	1	0.99218065	93.371881
UP_SEQ_FEATURE	repeat:Kelch 2	3	0.15625	0.1558101	190	71	19113	4.250481838	1	0.99218065	93.371881
UP_SEQ_FEATURE	repeat:Kelch 1	3	0.15625	0.1558101	190	71	19113	4.250481838	1	0.99218065	93.371881

Annotation Cluster 19 Enrichment Score: 0.8178443266820353

Category	Term	Count	%	PValue	List Total	Pop Hits	Pop Total	Fold Enrichment	Bonferroni	Benjamini	FDR
GOTERM_BP_FAT	GO:0046887~positive regulation of hormone secretion	3	0.15625	0.06091595	152	36	13528	7.416666667	1	0.74637643	64.4877879
GOTERM_BP_FAT	GO:0046883~regulation of hormone secretion	3	0.15625	0.16788902	152	66	13528	4.045454545	1	0.88229992	95.1560932
GOTERM_BP_FAT	GO:0051047~positive regulation of secretion	3	0.15625	0.34412299	152	109	13528	2.449541284	1	0.95128116	99.9039206

Annotation Cluster 20 Enrichment Score: 0.812073369751256

Category	Term	Count	%	PValue	List Total	Pop Hits	Pop Total	Fold Enrichment	Bonferroni	Benjamini	FDR
GOTERM_BP_FAT	GO:0008406~gonad development	4	0.20833333	0.12990665	152	112	13528	3.178571429	1	0.85130747	89.8957025
GOTERM_BP_FAT	GO:0048608~reproductive structure development	4	0.20833333	0.1665513	152	126	13528	2.825396825	1	0.88431253	95.0262124
GOTERM_BP_FAT	GO:0045137~development of primary sexual characteristics	4	0.20833333	0.16927799	152	127	13528	2.803149606	1	0.88040805	95.2875742

Annotation Cluster 21 Enrichment Score: 0.7782202112464123

Category	Term	Count	%	PValue	List Total	Pop Hits	Pop Total	Fold Enrichment	Bonferroni	Benjamini	FDR
GOTERM_BP_FAT	GO:0042325~regulation of phosphorylation	9	0.46875	0.15050721	152	466	13528	1.71888412	1	0.86926444	93.1908625
GOTERM_BP_FAT	GO:0051174~regulation of phosphorus metabolic process	9	0.46875	0.17534405	152	485	13528	1.651546392	1	0.88205182	95.8234832
GOTERM_BP_FAT	GO:0019220~regulation of phosphate metabolic process	9	0.46875	0.17534405	152	485	13528	1.651546392	1	0.88205182	95.8234832

Annotation Cluster 22 Enrichment Score: 0.7620303697761263

Category	Term	Count	%	PValue	List Total	Pop Hits	Pop Total	Fold Enrichment	Bonferroni	Benjamini	FDR
GOTERM_BP_FAT	GO:0008585~female gonad development	3	0.15625	0.16000002	152	64	13528	4.171875	1	0.8775137	94.3415174
GOTERM_BP_FAT	GO:0046660~female sex differentiation	3	0.15625	0.17984337	152	69	13528	3.869565217	1	0.88317143	96.1834063
GOTERM_BP_FAT	GO:0046545~development of primary female sexual characteristics	3	0.15625	0.17984337	152	69	13528	3.869565217	1	0.88317143	96.1834063

Annotation Cluster 23 Enrichment Score: 0.7580736618881148

Category	Term	Count	%	PValue	List Total	Pop Hits	Pop Total	Fold Enrichment	Bonferroni	Benjamini	FDR
GOTERM_BP_FAT	GO:0010558~negative regulation of macromolecule biosynthetic process	10	0.52083333	0.15847676	152	547	13528	1.627056673	1	0.87705349	94.1701005
GOTERM_BP_FAT	GO:0031327~negative regulation of cellular biosynthetic process	10	0.52083333	0.17565985	152	561	13528	1.586452763	1	0.88055417	95.8497508
GOTERM_BP_FAT	GO:0009890~negative regulation of biosynthetic process	10	0.52083333	0.19104731	152	573	13528	1.553228621	1	0.88489752	96.9571869

Annotation Cluster 24 Enrichment Score: 0.7104846798558776

Category	Term	Count	%	PValue	List Total	Pop Hits	Pop Total	Fold Enrichment	Bonferroni	Benjamini	FDR
UP_SEQ_FEATURE	repeat:TPR 2	4	0.20833333	0.18827223	190	151	19113	2.664761241	1	0.99556619	96.4638403
UP_SEQ_FEATURE	repeat:TPR 1	4	0.20833333	0.18827223	190	151	19113	2.664761241	1	0.99556619	96.4638403
SP_PIR_KEYWORDS	tpr repeat	4	0.20833333	0.20843633	190	160	19235	2.530921053	1	0.79847473	95.0357477

Annotation Cluster 25 Enrichment Score: 0.6941934530339083

Category	Term	Count	%	PValue	List Total	Pop Hits	Pop Total	Fold Enrichment	Bonferroni	Benjamini	FDR

GOTERM_BP_FAT	GO:0050871~positive regulation of B cell activation	3	0.15625	0.05216177	152	33	13528	8.090909091	1	0.70679218	58.6231502
GOTERM_BP_FAT	GO:0002706~regulation of lymphocyte mediated immunity	3	0.15625	0.12179396	152	54	13528	4.944444444	1	0.84891882	88.2266753
GOTERM_BP_FAT	GO:0050671~positive regulation of lymphocyte proliferation	3	0.15625	0.12550562	152	55	13528	4.854545455	1	0.84641614	89.0200616
GOTERM_BP_FAT	GO:0070665~positive regulation of leukocyte proliferation	3	0.15625	0.12924421	152	56	13528	4.767857143	1	0.85254291	89.7682342
GOTERM_BP_FAT	GO:0032946~positive regulation of mononuclear cell proliferation	3	0.15625	0.12924421	152	56	13528	4.767857143	1	0.85254291	89.7682342
GOTERM_BP_FAT	GO:0002703~regulation of leukocyte mediated immunity	3	0.15625	0.14830488	152	61	13528	4.37704918	1	0.86738723	92.8941724
GOTERM_BP_FAT	GO:0050670~regulation of lymphocyte proliferation	3	0.15625	0.23695496	152	83	13528	3.21686747	1	0.91123909	98.8376883
GOTERM_BP_FAT	GO:0032944~regulation of mononuclear cell proliferation	3	0.15625	0.24108586	152	84	13528	3.178571429	1	0.91141647	98.9371096
GOTERM_BP_FAT	GO:0070663~regulation of leukocyte proliferation	3	0.15625	0.24108586	152	84	13528	3.178571429	1	0.91141647	98.9371096
GOTERM_BP_FAT	GO:0051251~positive regulation of lymphocyte activation	3	0.15625	0.29489866	152	97	13528	2.75257732	1	0.93557858	99.6835226
GOTERM_BP_FAT	GO:0002697~regulation of immune effector process	3	0.15625	0.31139441	152	101	13528	2.643564356	1	0.93932122	99.7857157
GOTERM_BP_FAT	GO:0002696~positive regulation of leukocyte activation	3	0.15625	0.33189949	152	106	13528	2.518867925	1	0.9468453	99.8697647
GOTERM_BP_FAT	GO:0050867~positive regulation of cell activation	3	0.15625	0.35223297	152	111	13528	2.405405405	1	0.95328513	99.9217254
GOTERM_BP_FAT	GO:0046649~lymphocyte activation	3	0.15625	0.65440002	152	199	13528	1.341708543	1	0.99606695	99.9999975

Annotation Cluster 26 Enrichment Score: 0.6765659901101612

Category	Term	Count	%	PValue	List Total	Pop Hits	Pop Total	Fold Enrichment	Bonferroni	Benjamini	FDR
GOTERM_BP_FAT	GO:0043066~negative regulation of apoptosis	7	0.36458333	0.20420502	152	354	13528	1.759887006	1	0.88926859	97.6774758
GOTERM_BP_FAT	GO:0043069~negative regulation of programmed cell death	7	0.36458333	0.21297146	152	359	13528	1.735376045	1	0.89571197	98.0648423
GOTERM_BP_FAT	GO:0060548~negative regulation of cell death	7	0.36458333	0.21474062	152	360	13528	1.730555556	1	0.89632835	98.1352652

Annotation Cluster 27 Enrichment Score: 0.6567467846979181

Category	Term	Count	%	PValue	List Total	Pop Hits	Pop Total	Fold Enrichment	Bonferroni	Benjamini	FDR
GOTERM_BP_FAT	GO:0050804~regulation of synaptic transmission	4	0.20833333	0.19437068	152	136	13528	2.617647059	1	0.88607327	97.1566848
GOTERM_BP_FAT	GO:0051969~regulation of transmission of nerve impulse	4	0.20833333	0.22625108	152	147	13528	2.421768707	1	0.90534434	98.5378951
GOTERM_BP_FAT	GO:0031644~regulation of neurological system process	4	0.20833333	0.24352202	152	153	13528	2.326797386	1	0.91255397	98.991938

Annotation Cluster 28 Enrichment Score: 0.6390361671892503

Category	Term	Count	%	PValue	List Total	Pop Hits	Pop Total	Fold Enrichment	Bonferroni	Benjamini	FDR
GOTERM_CC_FAT	GO:0031981~nuclear lumen	20	1.04166667	0.07786691	120	1450	12782	1.469195402	1	0.66116	65.4932397
GOTERM_CC_FAT	GO:0070013~intracellular organelle lumen	20	1.04166667	0.29515183	120	1779	12782	1.197489226	1	0.8860329	98.9856147
GOTERM_CC_FAT	GO:0043233~organelle lumen	20	1.04166667	0.33161191	120	1820	12782	1.170512821	1	0.89860556	99.4948243
GOTERM_CC_FAT	GO:0031974~membrane-enclosed lumen	20	1.04166667	0.36460852	120	1856	12782	1.147808908	1	0.90691842	99.740073

Annotation Cluster 29 Enrichment Score: 0.6222168616189436

Category	Term	Count	%	PValue	List Total	Pop Hits	Pop Total	Fold Enrichment	Bonferroni	Benjamini	FDR
INTERPRO	IPR018200:Peptidase C19, ubiquitin carboxyl-terminal hydrolase 2, conserved site	3	0.15625	0.13589836	183	59	16659	4.628785774	1	0.89722791	87.0101818
INTERPRO	IPR001394:Peptidase C19, ubiquitin carboxyl-terminal hydrolase 2	3	0.15625	0.14337129	183	61	16659	4.477022306	1	0.90185021	88.4948295
GOTERM_MF_FAT	GO:0004221~ubiquitin thiolesterase activity	3	0.15625	0.18333155	136	75	12983	3.818529412	1	0.93666792	93.5548923
GOTERM_MF_FAT	GO:0016790~thiolester hydrolase activity	3	0.15625	0.28671288	136	102	12983	2.807742215	1	0.9648453	98.9686236
GOTERM_BP_FAT	GO:0006511~ubiquitin-dependent protein catabolic process	3	0.15625	0.75605757	152	242	13528	1.103305785	1	0.99890805	100

Annotation Cluster 30 Enrichment Score: 0.6089332840474001

Category	Term	Count	%	PValue	List Total	Pop Hits	Pop Total	Fold Enrichment	Bonferroni	Benjamini	FDR
UP_SEQ_FEATURE	repeat:WD 6	5	0.26041667	0.12315954	190	192	19113	2.619654605	1	0.98667175	87.8256072
UP_SEQ_FEATURE	repeat:WD 5	5	0.26041667	0.20050325	190	233	19113	2.15868534	1	0.99613015	97.227417
UP_SEQ_FEATURE	repeat:WD 4	5	0.26041667	0.23750361	190	251	19113	2.00387922	1	0.99704623	98.7023434
UP_SEQ_FEATURE	repeat:WD 3	5	0.26041667	0.26967629	190	265	19113	1.898013903	1	0.99788466	99.3495992
UP_SEQ_FEATURE	repeat:WD 2	5	0.26041667	0.28700259	190	274	19113	1.83567038	1	0.99834564	99.5573019
UP_SEQ_FEATURE	repeat:WD 1	5	0.26041667	0.28700259	190	274	19113	1.83567038	1	0.99834564	99.5573019
SP_PIR_KEYWORDS	wd repeat	5	0.26041667	0.28772763	190	276	19235	1.834000763	1	0.85458857	98.7207563
INTERPRO	IPR019775:WD40 repeat, conserved site	5	0.26041667	0.35865596	183	277	16659	1.643191099	1	0.987571	99.7984277

Annotation Cluster 31 Enrichment Score: 0.5938113317343032

Category	Term	Count	%	PValue	List Total	Pop Hits	Pop Total	Fold Enrichment	Bonferroni	Benjamini	FDR
GOTERM_BP_FAT	GO:0010552~positive regulation of specific transcription from RNA polymerase II pro	3	0.15625	0.13300867	152	57	13528	4.684210526	1	0.8475662	90.4730055
GOTERM_BP_FAT	GO:0043193~positive regulation of gene-specific transcription	3	0.15625	0.25349684	152	87	13528	3.068965517	1	0.9145962	99.1899163
GOTERM_BP_FAT	GO:0010551~regulation of specific transcription from RNA polymerase II promoter	3	0.15625	0.28249101	152	94	13528	2.840425532	1	0.93042137	99.5781732

GOTERM_BP_FAT	GO:0032583~regulation of gene-specific transcription	3	0.15625	0.44248397	152	134	13528	1.992537313	1	0.9759355	99.9933892

Annotation Cluster 32 Enrichment Score: 0.5887066168740392

Category	Term	Count	%	PValue	List Total	Pop Hits	Pop Total	Fold Enrichment	Bonferroni	Benjamini	FDR
UP_SEQ_FEATURE	repeat:TPR 5	3	0.15625	0.17327446	190	76	19113	3.970844875	1	0.9936583	95.2583922
UP_SEQ_FEATURE	repeat:TPR 4	3	0.15625	0.24910586	190	97	19113	3.111177428	1	0.99702753	98.9850023
UP_SEQ_FEATURE	repeat:TPR 3	3	0.15625	0.39697362	190	138	19113	2.186842105	1	0.99985338	99.9697694

Annotation Cluster 33 Enrichment Score: 0.5743160778908978

Category	Term	Count	%	PValue	List Total	Pop Hits	Pop Total	Fold Enrichment	Bonferroni	Benjamini	FDR
GOTERM_BP_FAT	GO:0048514~blood vessel morphogenesis	5	0.26041667	0.21013516	152	211	13528	2.109004739	1	0.89373188	97.9467057
GOTERM_BP_FAT	GO:0001568~blood vessel development	5	0.26041667	0.29266792	152	245	13528	1.816326531	1	0.93496772	99.6666199
GOTERM_BP_FAT	GO:0001944~vasculature development	5	0.26041667	0.30773573	152	251	13528	1.772908367	1	0.93779331	99.7661705

Annotation Cluster 34 Enrichment Score: 0.5620197072086561

Category	Term	Count	%	PValue	List Total	Pop Hits	Pop Total	Fold Enrichment	Bonferroni	Benjamini	FDR
SP_PIR_KEYWORDS	Homeobox	5	0.26041667	0.21514031	190	242	19235	2.091670291	1	0.80065036	95.5495959
INTERPRO	IPR017970:Homeobox, conserved site	5	0.26041667	0.24567666	183	232	16659	1.96191351	1	0.9617029	98.0542894
INTERPRO	IPR001356:Homeobox	5	0.26041667	0.25560734	183	235	16659	1.936867806	1	0.96387786	98.3832026
SMART	SM00389:HOX	5	0.26041667	0.41808266	128	235	9079	1.509142287	1	0.99554741	99.7795326

Annotation Cluster 35 Enrichment Score: 0.561324926793967

Category	Term	Count	%	PValue	List Total	Pop Hits	Pop Total	Fold Enrichment	Bonferroni	Benjamini	FDR
UP_SEQ_FEATURE	domain:Cadherin 6	3	0.15625	0.1627554	190	73	19113	4.134030281	1	0.99299107	94.1936267
UP_SEQ_FEATURE	domain:Cadherin 5	3	0.15625	0.24178677	190	95	19113	3.1766759	1	0.99707217	98.8143352
UP_SEQ_FEATURE	domain:Cadherin 3	3	0.15625	0.26742765	190	102	19113	2.958668731	1	0.99798511	99.3167605
UP_SEQ_FEATURE	domain:Cadherin 4	3	0.15625	0.26742765	190	102	19113	2.958668731	1	0.99798511	99.3167605
UP_SEQ_FEATURE	domain:Cadherin 2	3	0.15625	0.27842227	190	105	19113	2.874135338	1	0.99813479	99.463775
UP_SEQ_FEATURE	domain:Cadherin 1	3	0.15625	0.27842227	190	105	19113	2.874135338	1	0.99813479	99.463775
INTERPRO	IPR002126:Cadherin	3	0.15625	0.33065292	183	108	16659	2.528688525	1	0.9845312	99.6337487
SMART	SM00112:CA	3	0.15625	0.44796049	128	108	9079	1.970269097	1	0.99568753	99.8784613

Annotation Cluster 36 Enrichment Score: 0.5207799034483174

Category	Term	Count	%	PValue	List Total	Pop Hits	Pop Total	Fold Enrichment	Bonferroni	Benjamini	FDR
SP_PIR_KEYWORDS	voltage-gated channel	4	0.20833333	0.18339195	190	150	19235	2.699649123	1	0.78188612	92.5929587
GOTERM_MF_FAT	GO:0022843~voltage-gated cation channel activity	4	0.20833333	0.1970188	136	147	12983	2.597639056	1	0.9433048	94.8731336
GOTERM_MF_FAT	GO:0022832~voltage-gated channel activity	4	0.20833333	0.33087499	136	195	12983	1.958220211	1	0.97399695	99.5658629
GOTERM_MF_FAT	GO:0005244~voltage-gated ion channel activity	4	0.20833333	0.33087499	136	195	12983	1.958220211	1	0.97399695	99.5658629
GOTERM_MF_FAT	GO:0022836~gated channel activity	4	0.20833333	0.62933904	136	310	12983	1.231783681	1	0.98897353	99.9998539

Annotation Cluster 37 Enrichment Score: 0.5128157922683798

Category	Term	Count	%	PValue	List Total	Pop Hits	Pop Total	Fold Enrichment	Bonferroni	Benjamini	FDR
GOTERM_BP_FAT	GO:0030308~negative regulation of cell growth	3	0.15625	0.274209	152	92	13528	2.902173913	1	0.92707313	99.4903918
GOTERM_BP_FAT	GO:0045792~negative regulation of cell size	3	0.15625	0.30315482	152	99	13528	2.696969697	1	0.93749111	99.7393351
GOTERM_BP_FAT	GO:0045926~negative regulation of growth	3	0.15625	0.34818211	152	110	13528	2.427272727	1	0.95192707	99.9132602

Annotation Cluster 38 Enrichment Score: 0.49486715387923813

Category	Term	Count	%	PValue	List Total	Pop Hits	Pop Total	Fold Enrichment	Bonferroni	Benjamini	FDR
GOTERM_BP_FAT	GO:0016481~negative regulation of transcription	8	0.41666667	0.25299411	152	459	13528	1.551198257	1	0.91664397	99.1808827
GOTERM_BP_FAT	GO:0010629~negative regulation of gene expression	8	0.41666667	0.33227683	152	504	13528	1.412698413	1	0.94625116	99.8709711
GOTERM_BP_FAT	GO:0045934~negative regulation of nucleobase, nucleoside, nucleotide and nucleic a	8	0.41666667	0.34681592	152	512	13528	1.390625	1	0.95195405	99.9102164
GOTERM_BP_FAT	GO:0051172~negative regulation of nitrogen compound metabolic process	8	0.41666667	0.35960183	152	519	13528	1.371868979	1	0.95622158	99.9351704

Annotation Cluster 39 Enrichment Score: 0.477279017156909

Category	Term	Count	%	PValue	List Total	Pop Hits	Pop Total	Fold Enrichment	Bonferroni	Benjamini	FDR
SP_PIR_KEYWORDS	potassium channel	3	0.15625	0.17859656	190	78	19235	3.893724696	1	0.79417136	92.0143052
GOTERM_MF_FAT	GO:0005249~voltage-gated potassium channel activity	3	0.15625	0.27900063	136	100	12983	2.863897059	1	0.96827204	98.8069769
SP_PIR_KEYWORDS	potassium transport	3	0.15625	0.31229233	190	115	19235	2.640961098	1	0.85608258	99.1850382

SP_PIR_KEYWORDS	potassium	3	0.15625	0.35177211	190	126	19235	2.410401003	1	0.87272413	99.6186908
GOTERM_MF_FAT	GO:0030955~potassium ion binding	3	0.15625	0.38527776	136	128	12983	2.237419577	1	0.97736844	99.8622709
GOTERM_MF_FAT	GO:0005267~potassium channel activity	3	0.15625	0.40363325	136	133	12983	2.153306059	1	0.97853867	99.908634
GOTERM_BP_FAT	GO:0006813~potassium ion transport	3	0.15625	0.53577817	152	160	13528	1.66875	1	0.9880849	99.9996763

Annotation Cluster 40 Enrichment Score: 0.4454808562721388

Category	Term	Count	%	PValue	List Total	Pop Hits	Pop Total	Fold Enrichment	Bonferroni	Benjamini	FDR
GOTERM_MF_FAT	GO:0005525~GTP binding	6	0.3125	0.34486143	136	372	12983	1.539729602	1	0.97372767	99.6738456
GOTERM_MF_FAT	GO:0019001~guanyl nucleotide binding	6	0.3125	0.36555809	136	382	12983	1.499422544	1	0.97796405	99.7888074
GOTERM_MF_FAT	GO:0032561~guanyl ribonucleotide binding	6	0.3125	0.36555809	136	382	12983	1.499422544	1	0.97796405	99.7888074

Annotation Cluster 41 Enrichment Score: 0.3371173016016064

Category	Term	Count	%	PValue	List Total	Pop Hits	Pop Total	Fold Enrichment	Bonferroni	Benjamini	FDR
GOTERM_BP_FAT	GO:0006886~intracellular protein transport	6	0.3125	0.40586083	152	374	13528	1.427807487	1	0.97071733	99.9811461
GOTERM_BP_FAT	GO:0034613~cellular protein localization	6	0.3125	0.48673612	152	411	13528	1.299270073	1	0.98320417	99.9983071
GOTERM_BP_FAT	GO:0070727~cellular macromolecule localization	6	0.3125	0.49314811	152	414	13528	1.289855072	1	0.98329194	99.9986238

Annotation Cluster 42 Enrichment Score: 0.3051807367031665

Category	Term	Count	%	PValue	List Total	Pop Hits	Pop Total	Fold Enrichment	Bonferroni	Benjamini	FDR
GOTERM_MF_FAT	GO:0005261~cation channel activity	5	0.26041667	0.32078777	136	275	12983	1.735695187	1	0.97305666	99.4683775
SP_PIR_KEYWORDS	ionic channel	5	0.26041667	0.38102921	190	318	19235	1.591774247	1	0.89304947	99.7893318
GOTERM_MF_FAT	GO:0046873~metal ion transmembrane transporter activity	5	0.26041667	0.44555033	136	328	12983	1.455232245	1	0.98200877	99.9659378
GOTERM_MF_FAT	GO:0005216~ion channel activity	5	0.26041667	0.57320295	136	386	12983	1.236570405	1	0.98620476	99.9990143
GOTERM_MF_FAT	GO:0022838~substrate specific channel activity	5	0.26041667	0.59755852	136	398	12983	1.199286876	1	0.98899774	99.9995551
GOTERM_MF_FAT	GO:0015267~channel activity	5	0.26041667	0.62491948	136	412	12983	1.158534409	1	0.99041217	99.9998285
GOTERM_MF_FAT	GO:0022803~passive transmembrane transporter activity	5	0.26041667	0.62682899	136	413	12983	1.155729241	1	0.98999543	99.9998399

Annotation Cluster 43 Enrichment Score: 0.3047676003036056

Category	Term	Count	%	PValue	List Total	Pop Hits	Pop Total	Fold Enrichment	Bonferroni	Benjamini	FDR
SP_PIR_KEYWORDS	Endocytosis	3	0.15625	0.24321811	190	96	19235	3.163651316	1	0.81890791	97.2129533
GOTERM_BP_FAT	GO:0010324~membrane invagination	3	0.15625	0.70770236	152	220	13528	1.213636364	1	0.99779029	99.9999998
GOTERM_BP_FAT	GO:0006897~endocytosis	3	0.15625	0.70770236	152	220	13528	1.213636364	1	0.99779029	99.9999998

Annotation Cluster 44 Enrichment Score: 0.30352990965517856

Category	Term	Count	%	PValue	List Total	Pop Hits	Pop Total	Fold Enrichment	Bonferroni	Benjamini	FDR
GOTERM_MF_FAT	GO:0046872~metal ion binding	45	2.34375	0.4622854	136	4140	12983	1.037643862	1	0.97824517	99.9775058
GOTERM_MF_FAT	GO:0043169~cation binding	45	2.34375	0.49230309	136	4179	12983	1.027960179	1	0.98090407	99.9896647
GOTERM_MF_FAT	GO:0043167~ion binding	45	2.34375	0.53984308	136	4241	12983	1.01293223	1	0.98220404	99.9972695

Annotation Cluster 45 Enrichment Score: 0.2755564378236244

Category	Term	Count	%	PValue	List Total	Pop Hits	Pop Total	Fold Enrichment	Bonferroni	Benjamini	FDR
GOTERM_BP_FAT	GO:0000278~mitotic cell cycle	6	0.3125	0.39698785	152	370	13528	1.443243243	1	0.96815784	99.9759315
GOTERM_BP_FAT	GO:0022403~cell cycle phase	6	0.3125	0.49314811	152	414	13528	1.289855072	1	0.98329194	99.9986238
GOTERM_BP_FAT	GO:0022402~cell cycle process	6	0.3125	0.76133553	152	565	13528	0.945132743	1	0.99899387	100

Annotation Cluster 46 Enrichment Score: 0.27381846036969415

Category	Term	Count	%	PValue	List Total	Pop Hits	Pop Total	Fold Enrichment	Bonferroni	Benjamini	FDR
GOTERM_BP_FAT	GO:0043065~positive regulation of apoptosis	6	0.3125	0.5268335	152	430	13528	1.241860465	1	0.98716752	99.9995567
GOTERM_BP_FAT	GO:0043068~positive regulation of programmed cell death	6	0.3125	0.53304442	152	433	13528	1.233256351	1	0.98789691	99.9996434
GOTERM_BP_FAT	GO:0010942~positive regulation of cell death	6	0.3125	0.53716516	152	435	13528	1.227586207	1	0.98785418	99.9996918

Annotation Cluster 47 Enrichment Score: 0.24832200137528637

Category	Term	Count	%	PValue	List Total	Pop Hits	Pop Total	Fold Enrichment	Bonferroni	Benjamini	FDR
GOTERM_MF_FAT	GO:0004842~ubiquitin-protein ligase activity	3	0.15625	0.45357884	136	147	12983	1.948229292	1	0.97924665	99.9720417
GOTERM_MF_FAT	GO:0019787~small conjugating protein ligase activity	3	0.15625	0.51740641	136	166	12983	1.725239192	1	0.97987251	99.9947981
GOTERM_MF_FAT	GO:0016881~acid-amino acid ligase activity	3	0.15625	0.6214777	136	201	12983	1.424824407	1	0.99127361	99.9998059
GOTERM_MF_FAT	GO:0016879~ligase activity, forming carbon-nitrogen bonds	3	0.15625	0.69630875	136	231	12983	1.239782277	1	0.99406943	99.9999902

Annotation Cluster 48 Enrichment Score: 0.24203232699900953

Category	Term	Count	%	PValue	List Total	Pop Hits	Pop Total	Fold Enrichment	Bonferroni	Benjamini	FDR
GOTERM_MF_FAT	GO:0032553~ribonucleotide binding	20	1.04166667	0.54693102	136	1836	12983	1.039904524	1	0.98249288	99.997787
GOTERM_MF_FAT	GO:0032555~purine ribonucleotide binding	20	1.04166667	0.54693102	136	1836	12983	1.039904524	1	0.98249288	99.997787
GOTERM_MF_FAT	GO:0017076~purine nucleotide binding	20	1.04166667	0.62811318	136	1918	12983	0.995445624	1	0.98949323	99.9998472

Annotation Cluster 49 Enrichment Score: 0.2319078563605851

Category	Term	Count	%	PValue	List Total	Pop Hits	Pop Total	Fold Enrichment	Bonferroni	Benjamini	FDR
INTERPRO	IPR019781:WD40 repeat, subgroup	4	0.20833333	0.4973715	183	242	16659	1.504674163	1	0.9969851	99.9933097
INTERPRO	IPR001680:WD40 repeat	4	0.20833333	0.55927586	183	266	16659	1.368914088	1	0.99850629	99.9989338
SMART	SM00320:WD40	4	0.20833333	0.72438501	128	266	9079	1.066611842	1	0.99992138	99.9999525

Annotation Cluster 50 Enrichment Score: 0.19027513117333955

Category	Term	Count	%	PValue	List Total	Pop Hits	Pop Total	Fold Enrichment	Bonferroni	Benjamini	FDR
GOTERM_BP_FAT	GO:0016477~cell migration	4	0.20833333	0.59838875	152	276	13528	1.289855072	1	0.99350888	99.9999702
GOTERM_BP_FAT	GO:0051674~localization of cell	4	0.20833333	0.67003207	152	307	13528	1.159609121	1	0.99656509	99.9999988
GOTERM_BP_FAT	GO:0048870~cell motility	4	0.20833333	0.67003207	152	307	13528	1.159609121	1	0.99656509	99.9999988

Annotation Cluster 51 Enrichment Score: 0.18549942298228525

Category	Term	Count	%	PValue	List Total	Pop Hits	Pop Total	Fold Enrichment	Bonferroni	Benjamini	FDR
SP_PIR_KEYWORDS	dna repair	3	0.15625	0.55726528	190	191	19235	1.590107468	1	0.9386914	99.9971554
SP_PIR_KEYWORDS	DNA damage	3	0.15625	0.60064908	190	205	19235	1.481514763	1	0.95082214	99.9992438
GOTERM_BP_FAT	GO:0006281~DNA repair	3	0.15625	0.82950486	152	284	13528	0.940140845	1	0.99972073	100

Annotation Cluster 52 Enrichment Score: 0.16840234542499347

Category	Term	Count	%	PValue	List Total	Pop Hits	Pop Total	Fold Enrichment	Bonferroni	Benjamini	FDR
SP_PIR_KEYWORDS	mrna processing	4	0.20833333	0.47191629	190	260	19235	1.557489879	1	0.91273994	99.9726108
GOTERM_BP_FAT	GO:0006397~mRNA processing	4	0.20833333	0.69919807	152	321	13528	1.109034268	1	0.99755846	99.9999997
GOTERM_BP_FAT	GO:0006396~RNA processing	4	0.20833333	0.94695158	152	547	13528	0.650822669	1	0.9999976	100

Annotation Cluster 53 Enrichment Score: 0.15680780480983728

Category	Term	Count	%	PValue	List Total	Pop Hits	Pop Total	Fold Enrichment	Bonferroni	Benjamini	FDR
GOTERM_BP_FAT	GO:0000122~negative regulation of transcription from RNA polymerase II promoter	4	0.20833333	0.57324955	152	266	13528	1.338345865	1	0.99190323	99.9999191
GOTERM_BP_FAT	GO:0045892~negative regulation of transcription, DNA-dependent	4	0.20833333	0.76354627	152	356	13528	1	1	0.99901474	100
GOTERM_BP_FAT	GO:0051253~negative regulation of RNA metabolic process	4	0.20833333	0.77338813	152	362	13528	0.983425414	1	0.99911513	100

Annotation Cluster 54 Enrichment Score: 0.1270249887035057

Category	Term	Count	%	PValue	List Total	Pop Hits	Pop Total	Fold Enrichment	Bonferroni	Benjamini	FDR
GOTERM_BP_FAT	GO:0000280~nuclear division	3	0.15625	0.70770236	152	220	13528	1.213636364	1	0.99779029	99.9999998
GOTERM_BP_FAT	GO:0007067~mitosis	3	0.15625	0.70770236	152	220	13528	1.213636364	1	0.99779029	99.9999998
GOTERM_BP_FAT	GO:0000087~M phase of mitotic cell cycle	3	0.15625	0.71704723	152	224	13528	1.191964286	1	0.99807759	99.9999999
GOTERM_BP_FAT	GO:0048285~organelle fission	3	0.15625	0.72837675	152	229	13528	1.165938865	1	0.99835686	100
GOTERM_BP_FAT	GO:0000279~M phase	3	0.15625	0.88566591	152	329	13528	0.811550152	1	0.99994821	100

Annotation Cluster 55 Enrichment Score: 0.12493530672112069

Category	Term	Count	%	PValue	List Total	Pop Hits	Pop Total	Fold Enrichment	Bonferroni	Benjamini	FDR
GOTERM_MF_FAT	GO:0005524~ATP binding	15	0.78125	0.6843641	136	1477	12983	0.969497989	1	0.99344681	99.9999834
GOTERM_MF_FAT	GO:0032559~adenyl ribonucleotide binding	15	0.78125	0.70387491	136	1497	12983	0.956545444	1	0.99430511	99.999993
GOTERM_MF_FAT	GO:0030554~adenyl nucleotide binding	15	0.78125	0.77457022	136	1577	12983	0.908020627	1	0.99806079	99.9999998
GOTERM_MF_FAT	GO:0001883~purine nucleoside binding	15	0.78125	0.79339866	136	1601	12983	0.894408825	1	0.99841289	99.9999999
GOTERM_MF_FAT	GO:0001882~nucleoside binding	15	0.78125	0.80165684	136	1612	12983	0.888305539	1	0.99842153	100

Annotation Cluster 56 Enrichment Score: 0.10108560465337402

Category	Term	Count	%	PValue	List Total	Pop Hits	Pop Total	Fold Enrichment	Bonferroni	Benjamini	FDR
SP_PIR_KEYWORDS	Protease	5	0.26041667	0.70377458	190	484	19235	1.045835146	1	0.97359774	99.9999837
GOTERM_MF_FAT	GO:0070011~peptidase activity, acting on L-amino acid peptides	5	0.26041667	0.82825734	136	549	12983	0.869428372	1	0.9990326	100
GOTERM_MF_FAT	GO:0008233~peptidase activity	5	0.26041667	0.85338365	136	574	12983	0.831561283	1	0.99943237	100

Annotation Cluster 57 Enrichment Score: 0.10064131605534558

Category	Term	Count	%	PValue	List Total	Pop Hits	Pop Total	Fold Enrichment	Bonferroni	Benjamini	FDR
UP_SEQ_FEATURE	domain:PH	3	0.15625	0.68245288	190	237	19113	1.273351099	1	0.99999997	99.999999
INTERPRO	IPR001849:Pleckstrin homology	3	0.15625	0.80884271	183	277	16659	0.985914659	1	0.99999216	100
SMART	SM00233:PH	3	0.15625	0.90393937	128	277	9079	0.768191561	1	0.99999939	100

Annotation Cluster 58 Enrichment Score: 0.08197210928374583

Category	Term	Count	%	PValue	List Total	Pop Hits	Pop Total	Fold Enrichment	Bonferroni	Benjamini	FDR
GOTERM_BP_FAT	GO:0006875~cellular metal ion homeostasis	3	0.15625	0.64618834	152	196	13528	1.362244898	1	0.99590501	99.9999963
GOTERM_BP_FAT	GO:0055065~metal ion homeostasis	3	0.15625	0.67037011	152	205	13528	1.302439024	1	0.9965115	99.9999988
GOTERM_BP_FAT	GO:0030003~cellular cation homeostasis	3	0.15625	0.77942974	152	254	13528	1.051181102	1	0.99920236	100
GOTERM_BP_FAT	GO:0055080~cation homeostasis	3	0.15625	0.832452	152	286	13528	0.933566434	1	0.99973524	100
GOTERM_BP_FAT	GO:0006873~cellular ion homeostasis	3	0.15625	0.92434887	152	374	13528	0.713903743	1	0.99999045	100
GOTERM_BP_FAT	GO:0055082~cellular chemical homeostasis	3	0.15625	0.92846575	152	380	13528	0.702631579	1	0.99999228	100
GOTERM_BP_FAT	GO:0050801~ion homeostasis	3	0.15625	0.94556305	152	409	13528	0.652811736	1	0.99999742	100
GOTERM_BP_FAT	GO:0019725~cellular homeostasis	3	0.15625	0.96854743	152	466	13528	0.572961373	1	0.99999971	100

Annotation Cluster 59 Enrichment Score: 0.03524890035741495

Category	Term	Count	%	PValue	List Total	Pop Hits	Pop Total	Fold Enrichment	Bonferroni	Benjamini	FDR
GOTERM_CC_FAT	GO:0031966~mitochondrial membrane	3	0.15625	0.88586923	120	394	12782	0.811040609	1	0.99940651	100
GOTERM_CC_FAT	GO:0005740~mitochondrial envelope	3	0.15625	0.90569812	120	419	12782	0.762649165	1	0.99965791	100
GOTERM_CC_FAT	GO:0044429~mitochondrial part	3	0.15625	0.97701271	120	595	12782	0.537058824	1	0.99999464	100

Supplementary Table 7. GO annotation of ncRNA target genes in SCA 1 frontal cortex

Annotation Cluster 1 Enrichment Score: 2.093823995273662

Category	Term	Count	%	PValue	List Total	Pop Hits	Pop Total	Fold Enrichme	Bonferroni	Benjamini	FDR
GOTERM_CC_FAT	GO:0031981~nuclear lumen	31	1.36263736	0.00135025	151	1450	12782	1.80973738	0.30002206	0.06885595	1.75474895
GOTERM_CC_FAT	GO:0043233~organelle lumen	35	1.53846154	0.00347823	151	1820	12782	1.62786551	0.60142247	0.12313971	4.46257555
GOTERM_CC_FAT	GO:0031974~membrane-enclosed lumen	35	1.53846154	0.00475695	151	1856	12782	1.59629053	0.71601416	0.14559834	6.05639517
GOTERM_CC_FAT	GO:0070013~intracellular organelle lumen	33	1.45054945	0.00818848	151	1779	12782	1.57021766	0.88589749	0.21430313	10.2129413
GOTERM_CC_FAT	GO:0044451~nucleoplasm part	14	0.61538462	0.01361378	151	555	12782	2.13529026	0.97318319	0.26033695	16.4392843
GOTERM_CC_FAT	GO:0016604~nuclear body	7	0.30769231	0.01427741	151	168	12782	3.52704194	0.97754862	0.25325462	17.1728885
GOTERM_CC_FAT	GO:0005654~nucleoplasm	19	0.83516484	0.01514609	151	882	12782	1.82350468	0.98221072	0.25008626	18.1241731
GOTERM_CC_FAT	GO:0016607~nuclear speck	5	0.21978022	0.0329743	151	103	12782	4.10917508	0.9998569	0.35763505	35.5526597

Annotation Cluster 2 Enrichment Score: 2.074197142812731

Category	Term	Count	%	PValue	List Total	Pop Hits	Pop Total	Fold Enrichme	Bonferroni	Benjamini	FDR
GOTERM_BP_FAT	GO:0045893~positive regulation of transcription, DNA-depende	17	0.74725275	0.00101967	189	477	13528	2.55095227	0.7624681	0.7624681	1.66818491
GOTERM_BP_FAT	GO:0051254~positive regulation of RNA metabolic process	17	0.74725275	0.00111274	189	481	13528	2.52973853	0.7916883	0.54358823	1.81912773
SP_PIR_KEYWORDS	activator	16	0.7032967	0.00118936	225	520	19235	2.63042735	0.28933237	0.06602918	1.56713846
GOTERM_BP_FAT	GO:0043193~positive regulation of gene-specific transcription	7	0.30769231	0.00132226	189	87	13528	5.7590464	0.84499391	0.46282443	2.15815625
GOTERM_MF_FAT	GO:0003700~transcription factor activity	25	1.0989011	0.00316294	177	975	12983	1.88077647	0.66157113	0.19481907	4.22596028
GOTERM_BP_FAT	GO:0045941~positive regulation of transcription	17	0.74725275	0.00540711	189	564	13528	2.15745431	0.99951883	0.72006999	8.55229243
GOTERM_BP_FAT	GO:0045944~positive regulation of transcription from RNA poly	13	0.57142857	0.00572553	189	371	13528	2.50807912	0.99969355	0.68518775	9.03385617
GOTERM_BP_FAT	GO:0045935~positive regulation of nucleobase, nucleoside, nuc	18	0.79120879	0.00621634	189	624	13528	2.06471306	0.99984717	0.66655242	9.77148229
GOTERM_BP_FAT	GO:0010628~positive regulation of gene expression	17	0.74725275	0.00712733	189	581	13528	2.09432742	0.99995802	0.67366099	11.1257218
GOTERM_BP_FAT	GO:0051173~positive regulation of nitrogen compound metabo	18	0.79120879	0.00840287	189	644	13528	2.00059154	0.99999314	0.57226967	12.9898309
GOTERM_BP_FAT	GO:0010557~positive regulation of macromolecule biosynthetic	18	0.79120879	0.00970798	189	654	13528	1.97000146	0.99999893	0.55449718	14.8590929
GOTERM_MF_FAT	GO:0016563~transcription activator activity	13	0.57142857	0.01005157	177	410	12983	2.32574066	0.96841364	0.2501778	12.8634482
GOTERM_BP_FAT	GO:0032583~regulation of gene-specific transcription	7	0.30769231	0.011027	189	134	13528	3.73908237	0.99999984	0.56057183	16.709892
GOTERM_BP_FAT	GO:0006355~regulation of transcription, DNA-dependent	37	1.62637363	0.0117548	189	1773	13528	1.49370481	0.99999994	0.56527048	17.7148699
GOTERM_BP_FAT	GO:0031328~positive regulation of cellular biosynthetic process	18	0.79120879	0.01481155	189	685	13528	1.88084811	1	0.55455241	21.8126905
GOTERM_BP_FAT	GO:0051252~regulation of RNA metabolic process	37	1.62637363	0.016271	189	1813	13528	1.46074938	1	0.57517968	23.7008601
GOTERM_BP_FAT	GO:0009891~positive regulation of biosynthetic process	18	0.79120879	0.01684416	189	695	13528	1.85378554	1	0.57464956	24.430597
GOTERM_BP_FAT	GO:0006357~regulation of transcription from RNA polymerase	18	0.79120879	0.02483191	189	727	13528	1.77218838	1	0.63661149	33.9420038
GOTERM_BP_FAT	GO:0010552~positive regulation of specific transcription from R	4	0.17582418	0.04473244	189	57	13528	5.02292769	1	0.72462576	52.9813327
GOTERM_BP_FAT	GO:0010604~positive regulation of macromolecule metabolic p	19	0.83516484	0.05226711	189	857	13528	1.58688176	1	0.72858779	58.7369433
GOTERM_BP_FAT	GO:0010551~regulation of specific transcription from RNA poly	4	0.17582418	0.1426441	189	94	13528	3.04581785	1	0.84081626	92.0958333

Annotation Cluster 3 Enrichment Score: 2.0526391790309004

Category	Term	Count	%	PValue	List Total	Pop Hits	Pop Total	Fold Enrichme	Bonferroni	Benjamini	FDR
SP_PIR_KEYWORDS	Transcription	40	1.75824176	0.00171054	225	2071	19235	1.65116154	0.38819723	0.06778536	2.2466732
GOTERM_MF_FAT	GO:0030528~transcription regulator activity	35	1.53846154	0.00197521	177	1512	12983	1.69792321	0.49144874	0.20180027	2.65883898
SP_PIR_KEYWORDS	transcription regulation	39	1.71428571	0.00211806	225	2026	19235	1.64564001	0.45584912	0.07324504	2.774996
SP_PIR_KEYWORDS	nucleus	69	3.03296703	0.00289581	225	4283	19235	1.37724337	0.56495384	0.08833085	3.77596615
GOTERM_MF_FAT	GO:0003700~transcription factor activity	25	1.0989011	0.00316294	177	975	12983	1.88077647	0.66157113	0.19481907	4.22596028
GOTERM_BP_FAT	GO:0006355~regulation of transcription, DNA-dependent	37	1.62637363	0.0117548	189	1773	13528	1.49370481	0.99999994	0.56527048	17.7148699
GOTERM_BP_FAT	GO:0045449~regulation of transcription	50	2.1978022	0.01272956	189	2601	13528	1.37594617	0.99999999	0.55979016	19.0430249
GOTERM_BP_FAT	GO:0051252~regulation of RNA metabolic process	37	1.62637363	0.016271	189	1813	13528	1.46074938	1	0.57517968	23.7008601
GOTERM_BP_FAT	GO:0006357~regulation of transcription from RNA polymerase	18	0.79120879	0.02483191	189	727	13528	1.77218838	1	0.63661149	33.9420038
SP_PIR_KEYWORDS	dna-binding	32	1.40659341	0.02792012	225	1868	19235	1.46447775	0.99970457	0.32091097	31.3292935
GOTERM_MF_FAT	GO:0003677~DNA binding	42	1.84615385	0.04246337	177	2331	12983	1.32162671	0.99999964	0.54207209	44.6455676
GOTERM_BP_FAT	GO:0006350~transcription	39	1.71428571	0.04970484	189	2101	13528	1.32864924	1	0.73559403	56.8583769

Annotation Cluster 4 Enrichment Score: 1.8185374872598186

Category	Term	Count	%	PValue	List Total	Pop Hits	Pop Total	Fold Enrichme	Bonferroni	Benjamini	FDR
GOTERM_MF_FAT	GO:0005083~small GTPase regulator activity	13	0.57142857	3.58E-04	177	274	12983	3.48012289	0.11529874	0.11529874	0.48703183
GOTERM_MF_FAT	GO:0030695~GTPase regulator activity	14	0.61538462	0.00343232	177	404	12983	2.54184147	0.69144993	0.17797011	4.57810609
GOTERM_MF_FAT	GO:0060589~nucleoside-triphosphatase regulator activity	14	0.61538462	0.00413267	177	413	12983	2.48645025	0.75739065	0.16225192	5.48806734
GOTERM_BP_FAT	GO:0051056~regulation of small GTPase mediated signal transc	9	0.3956044	0.02487143	189	252	13528	2.55631141	1	0.62684081	33.9861396
GOTERM_BP_FAT	GO:0046578~regulation of Ras protein signal transduction	8	0.35164835	0.02727586	189	210	13528	2.72673217	1	0.62248255	36.6195573

GOTERM_BP_FAT	GO:0035023~regulation of Rho protein signal transduction	4	0.17582418	0.15907107	189	99	13528	2.89198867	1	0.86034801	94.254693
GOTERM_MF_FAT	GO:0005085~guanyl-nucleotide exchange factor activity	4	0.17582418	0.33985848	177	152	12983	1.93027059	1	0.90994532	99.6519074

Annotation Cluster 5 Enrichment Score: 1.7992053233381258

Category	Term	Count	%	PValue	List Total	Pop Hits	Pop Total	Fold Enrichme	Bonferroni	Benjamini	FDR
SP_PIR_KEYWORDS	serine/threonine-protein kinase	14	0.61538462	5.55E-04	225	381	19235	3.141324	0.14736351	0.05175338	0.73455818
SP_PIR_KEYWORDS	kinase	19	0.83516484	0.00121049	225	688	19235	2.36088501	0.29363345	0.05629044	1.5947688
KEGG_PATHWAY	hsa04010:MAPK signaling pathway	12	0.52747253	0.00132186	74	267	5085	3.08836927	0.12389882	0.04313334	1.45742681
UP_SEQ_FEATURE	binding site:ATP	16	0.7032967	0.00189561	225	542	19113	2.50765068	0.90233669	0.53948496	3.02885275
INTERPRO	IPR008271:Serine/threonine protein kinase, active site	13	0.57142857	0.00214315	215	354	16659	2.84544738	0.63830153	0.22449131	3.01980137
INTERPRO	IPR017442:Serine/threonine protein kinase-related	13	0.57142857	0.00239843	215	359	16659	2.80581719	0.67961118	0.20359226	3.37378673
GOTERM_MF_FAT	GO:0004672~protein kinase activity	18	0.79120879	0.0035929	177	606	12983	2.17872126	0.707995	0.16126033	4.78746402
SP_PIR_KEYWORDS	nucleotide-binding	33	1.45054945	0.00408395	225	1686	19235	1.67327007	0.69102493	0.10126966	5.28673829
UP_SEQ_FEATURE	nucleotide phosphate-binding region:ATP	22	0.96703297	0.00463778	225	962	19113	1.94264726	0.99665116	0.55699	7.25833173
GOTERM_MF_FAT	GO:0004674~protein serine/threonine kinase activity	14	0.61538462	0.00577	177	430	12983	2.38814873	0.86180011	0.17955255	7.58413203
INTERPRO	IPR017441:Protein kinase, ATP binding site	14	0.61538462	0.00607536	215	455	16659	2.38411449	0.94434065	0.38209312	8.34104534
INTERPRO	IPR002290:Serine/threonine protein kinase	10	0.43956044	0.00640549	215	259	16659	2.99164946	0.95244986	0.35282507	8.77520763
INTERPRO	IPR000719:Protein kinase, core	14	0.61538462	0.00871322	215	476	16659	2.27893297	0.98420659	0.40459908	11.757128
UP_SEQ_FEATURE	domain:Protein kinase	13	0.57142857	0.00955308	225	469	19113	2.35459844	0.99999225	0.69174787	14.4096697
SP_PIR_KEYWORDS	transferase	27	1.18681319	0.01173645	225	1394	19235	1.65581062	0.96623341	0.19084737	14.5036011
SP_PIR_KEYWORDS	atp-binding	25	1.0989011	0.02110855	225	1326	19235	1.61178146	0.99780815	0.26372413	24.6606123
UP_SEQ_FEATURE	active site:Proton acceptor	15	0.65934066	0.02327499	225	658	19113	1.93647416	1	0.83544807	31.7329542
GOTERM_BP_FAT	GO:0006468~protein amino acid phosphorylation	17	0.74725275	0.02365009	189	667	13528	1.8242942	1	0.64009662	32.6094468
GOTERM_MF_FAT	GO:0000166~nucleotide binding	42	1.84615385	0.02475001	177	2245	12983	1.37225473	0.99981048	0.43526622	28.9354541
SMART	SM00220:S_TKc	10	0.43956044	0.03205485	155	259	9079	2.26155187	0.98884671	0.67502441	31.84868
GOTERM_MF_FAT	GO:0032555~purine ribonucleotide binding	35	1.53846154	0.03425978	177	1836	12983	1.3982897	0.99999336	0.52533272	37.820154
GOTERM_MF_FAT	GO:0032553~ribonucleotide binding	35	1.53846154	0.03425978	177	1836	12983	1.3982897	0.99999336	0.52533272	37.820154
GOTERM_BP_FAT	GO:0006796~phosphate metabolic process	21	0.92307692	0.050545	189	973	13528	1.54482129	1	0.72883133	57.4830216
GOTERM_BP_FAT	GO:0006793~phosphorus metabolic process	21	0.92307692	0.050545	189	973	13528	1.54482129	1	0.72883133	57.4830216
GOTERM_BP_FAT	GO:0016310~phosphorylation	18	0.79120879	0.05341382	189	800	13528	1.61047619	1	0.72447494	59.5525315
GOTERM_MF_FAT	GO:0017076~purine nucleotide binding	35	1.53846154	0.05807471	177	1918	12983	1.3385088	1	0.58919471	55.7564118
GOTERM_MF_FAT	GO:0005524~ATP binding	25	1.0989011	0.20018766	177	1477	12983	1.24154168	1	0.83079351	95.2384284
GOTERM_MF_FAT	GO:0032559~adenyl ribonucleotide binding	25	1.0989011	0.21928448	177	1497	12983	1.22495462	1	0.84761389	96.5746735
GOTERM_MF_FAT	GO:0030554~adenyl nucleotide binding	25	1.0989011	0.303492	177	1577	12983	1.16281361	1	0.89449186	99.2770317
GOTERM_MF_FAT	GO:0001883~purine nucleoside binding	25	1.0989011	0.33075271	177	1601	12983	1.1453823	1	0.9063382	99.5804478
GOTERM_MF_FAT	GO:0001882~nucleoside binding	25	1.0989011	0.34348321	177	1612	12983	1.13756642	1	0.90915495	99.6770739

Annotation Cluster 6 Enrichment Score: 1.6513122740936903

Category	Term	Count	%	PValue	List Total	Pop Hits	Pop Total	Fold Enrichme	Bonferroni	Benjamini	FDR
UP_SEQ_FEATURE	DNA-binding region:A.T hook 1	3	0.13186813	0.00835298	225	12	19113	21.2366667	0.99996582	0.72347972	12.7130216
UP_SEQ_FEATURE	DNA-binding region:A.T hook 2	3	0.13186813	0.00835298	225	12	19113	21.2366667	0.99996582	0.72347972	12.7130216
INTERPRO	IPR017956:AT hook, DNA-binding, conserved site	3	0.13186813	0.04671663	215	27	16659	8.60930233	1	0.71630843	49.5297321
SMART	SM00384:AT_hook	3	0.13186813	0.07613705	155	27	9079	6.50824373	0.99998206	0.7030734	60.6243636

Annotation Cluster 7 Enrichment Score: 1.612487047995833

Category	Term	Count	%	PValue	List Total	Pop Hits	Pop Total	Fold Enrichme	Bonferroni	Benjamini	FDR
SP_PIR_KEYWORDS	bromodomain	4	0.17582418	0.01046113	225	39	19235	8.76809117	0.95110889	0.18225985	13.0275721
INTERPRO	IPR001487:Bromodomain	4	0.17582418	0.01456485	215	40	16659	7.74837209	0.99904568	0.53824604	18.9168861
SMART	SM00297:BROMO	4	0.17582418	0.029919	155	40	9079	5.85741935	0.98488167	0.87704338	30.0576224
INTERPRO	IPR018359:Bromodomain, conserved site	3	0.13186813	0.07784308	215	36	16659	6.45697674	1	0.83945575	68.5962571

Annotation Cluster 8 Enrichment Score: 1.6050509398757566

Category	Term	Count	%	PValue	List Total	Pop Hits	Pop Total	Fold Enrichme	Bonferroni	Benjamini	FDR
GOTERM_CC_FAT	GO:0045202~synapse	13	0.57142857	9.71E-04	151	355	12782	3.09982278	0.22619307	0.08192632	1.26460719
GOTERM_CC_FAT	GO:0044456~synapse part	9	0.3956044	0.00849693	151	246	12782	3.09691488	0.89489287	0.20170535	10.5781199
SP_PIR_KEYWORDS	synapse	7	0.30769231	0.03889479	225	213	19235	2.809494	0.99998864	0.37774407	40.9359238
GOTERM_CC_FAT	GO:0030054~cell junction	10	0.43956044	0.15552972	151	518	12782	1.63415071	1	0.71051989	89.0833665
SP_PIR_KEYWORDS	cell junction	8	0.35164835	0.18906855	225	399	19235	1.71406294	1	0.68546971	93.8059861

Annotation Cluster 9 Enrichment Score: 1.5524897785534717

Category	Term	Count	%	PValue	List Total	Pop Hits	Pop Total	Fold Enrichme	Bonferroni	Benjamini	FDR
GOTERM_CC_FAT	GO:0030135~coated vesicle	7	0.30769231	0.01111267	151	159	12782	3.72668583	0.94766906	0.23524292	13.6201913
GOTERM_CC_FAT	GO:0031410~cytoplasmic vesicle	15	0.65934066	0.01871366	151	642	12782	1.97778053	0.99317537	0.28285779	21.9261175
GOTERM_CC_FAT	GO:0030136~clathrin-coated vesicle	6	0.26373626	0.01961494	151	132	12782	3.84768212	0.99464542	0.26481675	22.8604539
GOTERM_CC_FAT	GO:0031982~vesicle	15	0.65934066	0.02587435	151	670	12782	1.89512701	0.99901292	0.31919979	29.06993
GOTERM_CC_FAT	GO:0016023~cytoplasmic membrane-bounded vesicle	13	0.57142857	0.0284662	151	550	12782	2.0007947	0.99951147	0.33053016	31.5032094
GOTERM_CC_FAT	GO:0031988~membrane-bounded vesicle	13	0.57142857	0.03519225	151	568	12782	1.93738924	0.99992195	0.34943697	37.4627262
GOTERM_CC_FAT	GO:0008021~synaptic vesicle	4	0.17582418	0.05991574	151	76	12782	4.45521087	0.99999992	0.45344892	55.4934458
SP_PIR_KEYWORDS	cytoplasmic vesicle	7	0.30769231	0.06002482	225	238	19235	2.51437908	0.99999998	0.45806782	56.0274757

Annotation Cluster 10 Enrichment Score: 1.5508890805674307

Category	Term	Count	%	PValue	List Total	Pop Hits	Pop Total	Fold Enrichme	Bonferroni	Benjamini	FDR
INTERPRO	IPR019787:Zinc finger, PHD-finger	7	0.30769231	7.88E-04	215	85	16659	6.38101231	0.31178308	0.31178308	1.12032704
INTERPRO	IPR001965:Zinc finger, PHD-type	7	0.30769231	0.0010639	215	90	16659	6.02651163	0.39622709	0.22297174	1.50984916
INTERPRO	IPR019786:Zinc finger, PHD-type, conserved site	7	0.30769231	0.00126179	215	93	16659	5.83210803	0.45034596	0.18085055	1.78833835
GOTERM_CC_FAT	GO:0035097~histone methyltransferase complex	4	0.17582418	0.00180411	151	21	12782	16.1236203	0.37918127	0.07637828	2.33816412
GOTERM_CC_FAT	GO:0034708~methyltransferase complex	4	0.17582418	0.00180411	151	21	12782	16.1236203	0.37918127	0.07637828	2.33816412
GOTERM_MF_FAT	GO:0042054~histone methyltransferase activity	5	0.21978022	0.00185384	177	39	12983	9.40388237	0.46985352	0.27188842	2.49737346
SMART	SM00249:PHD	7	0.30769231	0.00417056	155	90	9079	4.55577061	0.43827369	0.43827369	4.79967879
GOTERM_MF_FAT	GO:0008276~protein methyltransferase activity	5	0.21978022	0.00462528	177	50	12983	7.33502825	0.79515806	0.16152332	6.12328341
GOTERM_MF_FAT	GO:0018024~histone-lysine N-methyltransferase activity	4	0.17582418	0.00910213	177	32	12983	9.16878531	0.95615878	0.24745099	11.7174677
GOTERM_MF_FAT	GO:0016278~lysine N-methyltransferase activity	4	0.17582418	0.00910213	177	32	12983	9.16878531	0.95615878	0.24745099	11.7174677
GOTERM_MF_FAT	GO:0016279~protein-lysine N-methyltransferase activity	4	0.17582418	0.00910213	177	32	12983	9.16878531	0.95615878	0.24745099	11.7174677
UP_SEQ_FEATURE	domain:Post-SET	3	0.13186813	0.01472722	225	16	19113	15.9275	0.99999999	0.75321045	21.376593
INTERPRO	IPR003616:Post-SET zinc-binding region	3	0.13186813	0.01967676	215	17	16659	13.6735978	0.9999189	0.5438715	24.7255271
UP_SEQ_FEATURE	domain:SET	4	0.17582418	0.01970347	225	49	19113	6.93442177	1	0.82495053	27.5720484
UP_SEQ_FEATURE	zinc finger region:PHD-type	4	0.17582418	0.02304129	225	52	19113	6.53435897	1	0.85121859	31.467698
INTERPRO	IPR001214:SET	4	0.17582418	0.024979	215	49	16659	6.32520171	0.9999938	0.55038428	30.3397873
SMART	SM00508:PostSET	3	0.13186813	0.03290644	155	17	9079	10.3366224	0.99012239	0.60287213	32.5510125
GOTERM_MF_FAT	GO:0008170~N-methyltransferase activity	4	0.17582418	0.03499856	177	53	12983	5.53587038	0.99999489	0.51164124	38.465356
UP_SEQ_FEATURE	zinc finger region:PHD-type 2	3	0.13186813	0.04798297	225	30	19113	8.49466667	1	0.93544521	54.935442
SMART	SM00317:SET	4	0.17582418	0.05001885	155	49	9079	4.78156682	0.99915925	0.69278337	45.333317
UP_SEQ_FEATURE	zinc finger region:PHD-type 1	3	0.13186813	0.05693582	225	33	19113	7.72242424	1	0.93017931	61.3349414
SP_PIR_KEYWORDS	chromatin regulator	6	0.26373626	0.10403943	225	213	19235	2.40813772	1	0.57350242	76.7328085
GOTERM_BP_FAT	GO:0016568~chromatin modification	7	0.30769231	0.18371368	189	274	13528	1.82860232	1	0.88357105	96.4817943
GOTERM_BP_FAT	GO:0016570~histone modification	4	0.17582418	0.24062065	189	122	13528	2.34677769	1	0.91410563	98.9313956
SP_PIR_KEYWORDS	methyltransferase	4	0.17582418	0.24205348	225	146	19235	2.34216134	1	0.75837179	97.4737484
GOTERM_BP_FAT	GO:0043414~biopolymer methylation	3	0.13186813	0.24896039	189	69	13528	3.11203129	1	0.91582013	99.1092947
GOTERM_BP_FAT	GO:0016569~covalent chromatin modification	4	0.17582418	0.25550334	189	126	13528	2.27227681	1	0.9182708	99.2289684
GOTERM_BP_FAT	GO:0032259~methylation	3	0.13186813	0.28508038	189	76	13528	2.82539683	1	0.92875007	99.6048478
SP_PIR_KEYWORDS	s-adenosyl-l-methionine	3	0.13186813	0.33778782	225	103	19235	2.48996764	1	0.84705241	99.579132
GOTERM_BP_FAT	GO:0051276~chromosome organization	9	0.3956044	0.36158019	189	485	13528	1.32822779	1	0.95561203	99.9388653
GOTERM_BP_FAT	GO:0006325~chromatin organization	7	0.30769231	0.42925444	189	378	13528	1.32549481	1	0.96969336	99.9903652
GOTERM_BP_FAT	GO:0006730~one-carbon metabolic process	3	0.13186813	0.46291938	189	112	13528	1.91723356	1	0.97555195	99.9964644

Annotation Cluster 11 Enrichment Score: 1.525802504437583

Category	Term	Count	%	PValue	List Total	Pop Hits	Pop Total	Fold Enrichme	Bonferroni	Benjamini	FDR
KEGG_PATHWAY	hsa04360:Axon guidance	9	0.3956044	4.64E-04	74	129	5085	4.79415462	0.04539743	0.04539743	0.51435038
SP_PIR_KEYWORDS	neurogenesis	5	0.21978022	0.09133598	225	146	19235	2.92770167	1	0.54406038	71.9521765
SP_PIR_KEYWORDS	differentiation	6	0.26373626	0.62369253	225	460	19235	1.11507246	1	0.96453734	99.9997676

Annotation Cluster 12 Enrichment Score: 1.4734425429396898

Category	Term	Count	%	PValue	List Total	Pop Hits	Pop Total	Fold Enrichme	Bonferroni	Benjamini	FDR
KEGG_PATHWAY	hsa04510:Focal adhesion	9	0.3956044	0.00766938	74	201	5085	3.0768455	0.53693768	0.17508375	8.19038586
KEGG_PATHWAY	hsa05200:Pathways in cancer	11	0.48351648	0.01779907	74	328	5085	2.30450725	0.83402822	0.25868001	18.0726522
KEGG_PATHWAY	hsa05211:Renal cell carcinoma	4	0.17582418	0.07857933	74	70	5085	3.92664093	0.99972088	0.46715658	59.6813067

KEGG_PATHWAY	hsa05210:Colorectal cancer	4	0.17582418	0.11905956	74	84	5085	3.27220077	0.99999688	0.52558746	75.5126023

Annotation Cluster 13 Enrichment Score: 1.435905378140428

Category	Term	Count	%	PValue	List Total	Pop Hits	Pop Total	Fold Enrichme	Bonferroni	Benjamini	FDR
SP_PIR_KEYWORDS	ubl conjugation pathway	16	0.7032967	9.61E-04	225	509	19235	2.68727352	0.24120656	0.06667931	1.26840661
UP_SEQ_FEATURE	active site:Glycyl thioester intermediate	5	0.21978022	0.00876437	225	69	19113	6.15555556	0.99997945	0.69855421	13.2981524
GOTERM_BP_FAT	GO:0043632~modification-dependent macromolecule catabolic	16	0.7032967	0.01413905	189	574	13528	1.99516988	1	0.56656094	20.9279498
GOTERM_BP_FAT	GO:0019941~modification-dependent protein catabolic process	16	0.7032967	0.01413905	189	574	13528	1.99516988	1	0.56656094	20.9279498
INTERPRO	IPR000608:Ubiquitin-conjugating enzyme, E2	4	0.17582418	0.01556882	215	41	16659	7.55938741	0.99941134	0.52467992	20.089581
GOTERM_BP_FAT	GO:0051603~proteolysis involved in cellular protein catabolic p	16	0.7032967	0.0202711	189	600	13528	1.90871252	1	0.61781411	28.6587949
GOTERM_BP_FAT	GO:0044257~cellular protein catabolic process	16	0.7032967	0.02109189	189	603	13528	1.89921644	1	0.62050543	29.6379725
INTERPRO	IPR016135:Ubiquitin-conjugating enzyme/RWD-like	4	0.17582418	0.02366822	215	48	16659	6.45697674	0.99998827	0.55557492	28.9893182
GOTERM_BP_FAT	GO:0030163~protein catabolic process	16	0.7032967	0.02689003	189	622	13528	1.84120179	1	0.62648199	36.2037379
SMART	SM00212:UBCc	4	0.17582418	0.0318902	155	41	9079	5.71455547	0.98858182	0.7748187	31.7121154
GOTERM_BP_FAT	GO:0044265~cellular macromolecule catabolic process	17	0.74725275	0.04541105	189	725	13528	1.67835067	1	0.72306451	53.5290902
GOTERM_MF_FAT	GO:0004842~ubiquitin-protein ligase activity	6	0.26373626	0.04975957	177	147	12983	2.99388908	0.99999997	0.56448534	50.1258169
SP_PIR_KEYWORDS	ligase	8	0.35164835	0.06663506	225	305	19235	2.24233151	1	0.48299762	59.9592865
GOTERM_MF_FAT	GO:0019787~small conjugating protein ligase activity	6	0.26373626	0.07542524	177	166	12983	2.65121503	1	0.59098507	65.6601989
GOTERM_BP_FAT	GO:0009057~macromolecule catabolic process	17	0.74725275	0.07736134	189	781	13528	1.55800798	1	0.75355312	73.4913904
GOTERM_MF_FAT	GO:0016881~acid-amino acid ligase activity	6	0.26373626	0.1371684	177	201	12983	2.18956067	1	0.75379469	86.6128908
GOTERM_MF_FAT	GO:0016879~ligase activity, forming carbon-nitrogen bonds	6	0.26373626	0.20465898	177	231	12983	1.90520214	1	0.83133361	95.5887099
GOTERM_BP_FAT	GO:0006508~proteolysis	18	0.79120879	0.29623141	189	1054	13528	1.22237282	1	0.93508975	99.6950809
KEGG_PATHWAY	hsa04120:Ubiquitin mediated proteolysis	4	0.17582418	0.31406358	74	137	5085	2.00631288	1	0.70359643	98.4764461

Annotation Cluster 14 Enrichment Score: 1.3756972520387298

Category	Term	Count	%	PValue	List Total	Pop Hits	Pop Total	Fold Enrichme	Bonferroni	Benjamini	FDR
GOTERM_BP_FAT	GO:0070647~protein modification by small protein conjugation	7	0.30769231	0.02435211	189	160	13528	3.13148148	1	0.64000713	33.4040199
GOTERM_BP_FAT	GO:0016579~protein deubiquitination	3	0.13186813	0.05019887	189	26	13528	8.25885226	1	0.73270553	57.2267174
GOTERM_BP_FAT	GO:0070646~protein modification by small protein removal	3	0.13186813	0.06104888	189	29	13528	7.40448823	1	0.74473925	64.6088559

Annotation Cluster 15 Enrichment Score: 1.3158489422989044

Category	Term	Count	%	PValue	List Total	Pop Hits	Pop Total	Fold Enrichme	Bonferroni	Benjamini	FDR
GOTERM_BP_FAT	GO:0048666~neuron development	11	0.48351648	0.0202067	189	339	13528	2.32254842	1	0.62909967	28.5814325
GOTERM_BP_FAT	GO:0007610~behavior	12	0.52747253	0.06303837	189	469	13528	1.83138728	1	0.74054673	65.8252956
GOTERM_BP_FAT	GO:0007626~locomotory behavior	8	0.35164835	0.08858347	189	274	13528	2.08983123	1	0.77736215	78.3354787

Annotation Cluster 16 Enrichment Score: 1.2428754181599904

Category	Term	Count	%	PValue	List Total	Pop Hits	Pop Total	Fold Enrichme	Bonferroni	Benjamini	FDR
GOTERM_BP_FAT	GO:0050808~synapse organization	5	0.21978022	0.01018069	189	61	13528	5.86694423	0.99999945	0.55112297	15.5267779
GOTERM_BP_FAT	GO:0050804~regulation of synaptic transmission	6	0.26373626	0.04124387	189	136	13528	3.15779645	1	0.7095589	50.068416
GOTERM_BP_FAT	GO:0051969~regulation of transmission of nerve impulse	6	0.26373626	0.05431522	189	147	13528	2.92149876	1	0.72471563	60.1829953
GOTERM_BP_FAT	GO:0031644~regulation of neurological system process	6	0.26373626	0.06246169	189	153	13528	2.80693018	1	0.74240741	65.4767948
GOTERM_BP_FAT	GO:0044057~regulation of system process	6	0.26373626	0.42850008	189	309	13528	1.38983922	1	0.96991283	99.9901531

Annotation Cluster 17 Enrichment Score: 1.2333332992842232

Category	Term	Count	%	PValue	List Total	Pop Hits	Pop Total	Fold Enrichme	Bonferroni	Benjamini	FDR
GOTERM_CC_FAT	GO:0031252~cell leading edge	8	0.35164835	0.0011989	151	138	12782	4.90718879	0.27145102	0.07612186	1.55949001
GOTERM_CC_FAT	GO:0001726~ruffle	4	0.17582418	0.04400844	151	67	12782	5.05367204	0.99999308	0.39047066	44.5497133
GOTERM_MF_FAT	GO:0003779~actin binding	7	0.30769231	0.28137352	177	326	12983	1.57500607	1	0.89092444	98.8929377
SP_PIR_KEYWORDS	actin-binding	3	0.13186813	0.78535014	225	247	19235	1.03832659	1	0.99133624	99.9999999

Annotation Cluster 18 Enrichment Score: 1.2280709736732258

Category	Term	Count	%	PValue	List Total	Pop Hits	Pop Total	Fold Enrichme	Bonferroni	Benjamini	FDR
KEGG_PATHWAY	hsa04520:Adherens junction	7	0.30769231	7.54E-04	74	77	5085	6.24692875	0.07262693	0.03699788	0.83339132
BIOCARTA	h_salmonellaPathway:How does salmonella hijack a cell	3	0.13186813	0.00859517	37	6	1437	19.4189189	0.54017472	0.54017472	8.96342056
KEGG_PATHWAY	hsa04810:Regulation of actin cytoskeleton	8	0.35164835	0.03355221	74	215	5085	2.55688246	0.96705118	0.31559126	31.5316424
KEGG_PATHWAY	hsa05211:Renal cell carcinoma	4	0.17582418	0.07857933	74	70	5085	3.92664093	0.99972088	0.46715658	59.6813067
KEGG_PATHWAY	hsa05212:Pancreatic cancer	4	0.17582418	0.08392571	74	72	5085	3.81756757	0.99984402	0.46534159	62.2031512

KEGG_PATHWAY	hsa04370:VEGF signaling pathway	4	0.17582418	0.09223082	74	75	5085	3.66486486	0.99993726	0.47539039	65.8370955
KEGG_PATHWAY	hsa04722:Neurotrophin signaling pathway	5	0.21978022	0.10170964	74	124	5085	2.77081517	0.99997804	0.48848942	69.5942138
KEGG_PATHWAY	hsa04666:Fc gamma R-mediated phagocytosis	4	0.17582418	0.15524705	74	95	5085	2.89331437	0.99999995	0.56982162	84.6273871
GOTERM_BP_FAT	GO:0033674~positive regulation of kinase activity	6	0.26373626	0.22006596	189	231	13528	1.85913557	1	0.90315802	98.3401034
GOTERM_BP_FAT	GO:0051347~positive regulation of transferase activity	6	0.26373626	0.24040841	189	240	13528	1.78941799	1	0.91522576	98.9264599
KEGG_PATHWAY	hsa04062:Chemokine signaling pathway	5	0.21978022	0.28059031	74	187	5085	1.83733198	1	0.70468703	97.4145858

Annotation Cluster 19 Enrichment Score: 1.2074500429744521

Category	Term	Count	%	PValue	List Total	Pop Hits	Pop Total	Fold Enrichme	Bonferroni	Benjamini	FDR
GOTERM_MF_FAT	GO:0003702~RNA polymerase II transcription factor activity	8	0.35164835	0.04860212	177	244	12983	2.40492729	0.99999996	0.57342707	49.2914099
GOTERM_BP_FAT	GO:0006351~transcription, DNA-dependent	9	0.3956044	0.05141299	189	292	13528	2.20613177	1	0.72875372	58.1194401
GOTERM_BP_FAT	GO:0032774~RNA biosynthetic process	9	0.3956044	0.05486582	189	296	13528	2.17631918	1	0.72262372	60.563543
GOTERM_BP_FAT	GO:0006366~transcription from RNA polymerase II promoter	7	0.30769231	0.10793689	189	234	13528	2.14118392	1	0.81622427	84.7929643

Annotation Cluster 20 Enrichment Score: 1.1791775214711335

Category	Term	Count	%	PValue	List Total	Pop Hits	Pop Total	Fold Enrichme	Bonferroni	Benjamini	FDR
INTERPRO	IPR000504:RNA recognition motif, RNP-1	8	0.35164835	0.0193745	215	211	16659	2.93777141	0.99990613	0.56960777	24.3931297
INTERPRO	IPR012677:Nucleotide-binding, alpha-beta plait	8	0.35164835	0.02019548	215	213	16659	2.9101867	0.99993689	0.52474111	25.2927905
GOTERM_MF_FAT	GO:0003723~RNA binding	16	0.7032967	0.06338278	177	718	12983	1.63454668	1	0.59170878	59.0363617
SMART	SM00360:RRM	8	0.35164835	0.06755873	155	211	9079	2.2208225	0.99993577	0.70079376	56.0995978
SP_PIR_KEYWORDS	rna-binding	11	0.48351648	0.1006587	225	540	19235	1.74144033	1	0.57078373	75.5401732
UP_SEQ_FEATURE	domain:RRM 1	4	0.17582418	0.11548762	225	101	19113	3.36422442	1	0.96726764	86.3201602
UP_SEQ_FEATURE	domain:RRM 2	4	0.17582418	0.11548762	225	101	19113	3.36422442	1	0.96726764	86.3201602
UP_SEQ_FEATURE	domain:RRM	4	0.17582418	0.16387781	225	119	19113	2.85535014	1	0.98646601	94.5044904

Annotation Cluster 21 Enrichment Score: 1.091896205973138

Category	Term	Count	%	PValue	List Total	Pop Hits	Pop Total	Fold Enrichme	Bonferroni	Benjamini	FDR
GOTERM_BP_FAT	GO:0007517~muscle organ development	8	0.35164835	0.02812509	189	211	13528	2.71380927	1	0.62483711	37.5258443
GOTERM_BP_FAT	GO:0014706~striated muscle tissue development	5	0.21978022	0.08416866	189	119	13528	3.00742519	1	0.77117659	76.5385919
GOTERM_BP_FAT	GO:0060537~muscle tissue development	5	0.21978022	0.0965487	189	125	13528	2.86306878	1	0.78881086	81.2548772
GOTERM_BP_FAT	GO:0048738~cardiac muscle tissue development	3	0.13186813	0.18768278	189	57	13528	3.76719577	1	0.88392699	96.7535023

Annotation Cluster 22 Enrichment Score: 1.08444945047262

Category	Term	Count	%	PValue	List Total	Pop Hits	Pop Total	Fold Enrichme	Bonferroni	Benjamini	FDR
GOTERM_BP_FAT	GO:0007267~cell-cell signaling	17	0.74725275	0.00954664	189	600	13528	2.02800705	0.99999865	0.59385933	14.6300668
GOTERM_BP_FAT	GO:0007268~synaptic transmission	9	0.3956044	0.05639195	189	298	13528	2.16171301	1	0.72696836	61.6005505
GOTERM_BP_FAT	GO:0001505~regulation of neurotransmitter levels	4	0.17582418	0.06633546	189	67	13528	4.27323699	1	0.74388467	67.7551614
GOTERM_BP_FAT	GO:0019226~transmission of nerve impulse	9	0.3956044	0.11511396	189	350	13528	1.84054422	1	0.82766635	86.6894727
GOTERM_BP_FAT	GO:0050877~neurological system process	13	0.57142857	0.92004972	189	1210	13528	0.76900608	1	0.99998672	100

Annotation Cluster 23 Enrichment Score: 1.0393345743145601

Category	Term	Count	%	PValue	List Total	Pop Hits	Pop Total	Fold Enrichme	Bonferroni	Benjamini	FDR
GOTERM_CC_FAT	GO:0030135~coated vesicle	7	0.30769231	0.01111267	151	159	12782	3.72668583	0.94766906	0.23524292	13.6201913
GOTERM_CC_FAT	GO:0048471~perinuclear region of cytoplasm	6	0.26373626	0.25002517	151	288	12782	1.76352097	1	0.80818762	97.6941654
GOTERM_BP_FAT	GO:0048193~Golgi vesicle transport	4	0.17582418	0.27428	189	131	13528	2.18554869	1	0.92654309	99.4940093

Annotation Cluster 24 Enrichment Score: 0.9870244697758198

Category	Term	Count	%	PValue	List Total	Pop Hits	Pop Total	Fold Enrichme	Bonferroni	Benjamini	FDR
GOTERM_BP_FAT	GO:0043551~regulation of phosphoinositide 3-kinase activity	3	0.13186813	0.00277769	189	6	13528	35.7883598	0.98014313	0.62461432	4.48306005
GOTERM_BP_FAT	GO:0043552~positive regulation of phosphoinositide 3-kinase a	3	0.13186813	0.00277769	189	6	13528	35.7883598	0.98014313	0.62461432	4.48306005
GOTERM_BP_FAT	GO:0043550~regulation of lipid kinase activity	3	0.13186813	0.00803347	189	10	13528	21.4730159	0.9999884	0.61212515	12.4537892
GOTERM_BP_FAT	GO:0043549~regulation of kinase activity	10	0.43956044	0.06172244	189	357	13528	2.00495013	1	0.74336505	65.0251733
KEGG_PATHWAY	hsa05120:Epithelial cell signaling in Helicobacter pylori infectior	4	0.17582418	0.07339027	74	68	5085	4.04213037	0.99951058	0.47016521	57.0882
GOTERM_BP_FAT	GO:0051338~regulation of transferase activity	10	0.43956044	0.07548437	189	372	13528	1.92410536	1	0.75774562	72.5879934
KEGG_PATHWAY	hsa05211:Renal cell carcinoma	4	0.17582418	0.07857933	74	70	5085	3.92664093	0.99972088	0.46715658	59.6813067
KEGG_PATHWAY	hsa04722:Neurotrophin signaling pathway	5	0.21978022	0.10170964	74	124	5085	2.77081517	0.99997804	0.48848942	69.5942138
GOTERM_BP_FAT	GO:0042325~regulation of phosphorylation	11	0.48351648	0.11527942	189	466	13528	1.68957922	1	0.82504449	86.7304549
GOTERM_BP_FAT	GO:0045834~positive regulation of lipid metabolic process	3	0.13186813	0.13391375	189	46	13528	4.66804693	1	0.8308478	90.6586371

GOTERM_BP_FAT	GO:0051174~regulation of phosphorus metabolic process	11	0.48351648	0.1387674	189	485	13528	1.62338952	1	0.83454767	91.485397
GOTERM_BP_FAT	GO:0019220~regulation of phosphate metabolic process	11	0.48351648	0.1387674	189	485	13528	1.62338952	1	0.83454767	91.485397
GOTERM_BP_FAT	GO:0019216~regulation of lipid metabolic process	4	0.17582418	0.20416605	189	112	13528	2.55631141	1	0.88962548	97.6847107
GOTERM_BP_FAT	GO:0033674~positive regulation of kinase activity	6	0.26373626	0.22006596	189	231	13528	1.85913557	1	0.90315802	98.3401034
GOTERM_BP_FAT	GO:0051347~positive regulation of transferase activity	6	0.26373626	0.24040841	189	240	13528	1.78941799	1	0.91522576	98.9264599
KEGG_PATHWAY	hsa04062:Chemokine signaling pathway	5	0.21978022	0.28059031	74	187	5085	1.83733198	1	0.70468703	97.4145858
GOTERM_BP_FAT	GO:0045859~regulation of protein kinase activity	7	0.30769231	0.34738895	189	345	13528	1.45228127	1	0.95348508	99.9121521
GOTERM_BP_FAT	GO:0043085~positive regulation of catalytic activity	8	0.35164835	0.58814929	189	520	13528	1.1011803	1	0.99137018	99.9999556
GOTERM_BP_FAT	GO:0044093~positive regulation of molecular function	8	0.35164835	0.71121234	189	586	13528	0.97715658	1	0.99793781	99.9999999
GOTERM_BP_FAT	GO:0045860~positive regulation of protein kinase activity	3	0.13186813	0.81963149	189	223	13528	0.96291551	1	0.99969592	100

Annotation Cluster 25 Enrichment Score: 0.9810227227714099

Category	Term	Count	%	PValue	List Total	Pop Hits	Pop Total	Fold Enrichme	Bonferroni	Benjamini	FDR
GOTERM_BP_FAT	GO:0031346~positive regulation of cell projection organization	5	0.21978022	0.00404261	189	47	13528	7.61454464	0.99667933	0.68066601	6.46140712
GOTERM_BP_FAT	GO:0031344~regulation of cell projection organization	6	0.26373626	0.00796158	189	89	13528	4.82539683	0.99998716	0.64080422	12.3491137
GOTERM_BP_FAT	GO:0030182~neuron differentiation	14	0.61538462	0.00824121	189	438	13528	2.28784035	0.99999137	0.59217652	12.7556147
GOTERM_BP_FAT	GO:0031175~neuron projection development	10	0.43956044	0.00960061	189	256	13528	2.79596561	0.99999875	0.57238771	14.7067404
GOTERM_BP_FAT	GO:0016358~dendrite development	4	0.17582418	0.01247273	189	35	13528	8.18019652	0.99999998	0.56920538	18.6950585
GOTERM_BP_FAT	GO:0030030~cell projection organization	12	0.52747253	0.01390404	189	368	13528	2.33402346	1	0.57588496	20.616562
GOTERM_BP_FAT	GO:0048666~neuron development	11	0.48351648	0.0202067	189	339	13528	2.32254842	1	0.62909967	28.5814325
GOTERM_CC_FAT	GO:0030424~axon	6	0.26373626	0.03934812	151	159	12782	3.19430214	0.99997503	0.36920472	40.9016239
GOTERM_BP_FAT	GO:0050770~regulation of axonogenesis	4	0.17582418	0.04473244	189	57	13528	5.02292769	1	0.72462576	52.9813327
GOTERM_BP_FAT	GO:0050772~positive regulation of axonogenesis	3	0.13186813	0.04675493	189	25	13528	8.58920635	1	0.72677292	54.5961908
GOTERM_BP_FAT	GO:0007409~axonogenesis	7	0.30769231	0.05271897	189	193	13528	2.59604682	1	0.72566396	59.0601479
GOTERM_BP_FAT	GO:0048667~cell morphogenesis involved in neuron differentia	7	0.30769231	0.07163089	189	209	13528	2.3973064	1	0.75712417	70.6418988
GOTERM_BP_FAT	GO:0010975~regulation of neuron projection development	4	0.17582418	0.07359354	189	70	13528	4.09009826	1	0.7576078	71.6487451
GOTERM_BP_FAT	GO:0048812~neuron projection morphogenesis	7	0.30769231	0.07688112	189	213	13528	2.35228656	1	0.75560063	73.262956
GOTERM_BP_FAT	GO:0032535~regulation of cellular component size	8	0.35164835	0.08421627	189	271	13528	2.11296589	1	0.76737224	76.5586923
GOTERM_BP_FAT	GO:0010769~regulation of cell morphogenesis involved in diffe	4	0.17582418	0.09181869	189	77	13528	3.71827115	1	0.782324	79.5692828
GOTERM_BP_FAT	GO:0051130~positive regulation of cellular component organiza	6	0.26373626	0.1083473	189	181	13528	2.37270894	1	0.81421162	84.9079212
GOTERM_BP_FAT	GO:0045664~regulation of neuron differentiation	5	0.21978022	0.11429703	189	133	13528	2.69085412	1	0.82847838	86.48539
GOTERM_BP_FAT	GO:0000904~cell morphogenesis involved in differentiation	7	0.30769231	0.12310108	189	244	13528	2.05343048	1	0.83131522	88.5379458
GOTERM_BP_FAT	GO:0048858~cell projection morphogenesis	7	0.30769231	0.12620144	189	245	13528	2.04504913	1	0.83357722	89.1881995
GOTERM_BP_FAT	GO:0051960~regulation of nervous system development	6	0.26373626	0.12989159	189	192	13528	2.23677249	1	0.83173656	89.9169733
GOTERM_BP_FAT	GO:0043254~regulation of protein complex assembly	4	0.17582418	0.12994278	189	90	13528	3.18118754	1	0.82914075	89.9267499
GOTERM_BP_FAT	GO:0051129~negative regulation of cellular component organiz	5	0.21978022	0.13583996	189	142	13528	2.52030703	1	0.83283672	90.9953785
GOTERM_BP_FAT	GO:0032990~cell part morphogenesis	7	0.30769231	0.14686968	189	256	13528	1.95717593	1	0.84511636	92.7142654
GOTERM_BP_FAT	GO:0033043~regulation of organelle organization	6	0.26373626	0.18363886	189	217	13528	1.9790798	1	0.88534036	96.476473
GOTERM_BP_FAT	GO:0007411~axon guidance	4	0.17582418	0.18646171	189	107	13528	2.67576522	1	0.88580914	96.6720876
GOTERM_BP_FAT	GO:0032989~cellular component morphogenesis	9	0.3956044	0.18854811	189	397	13528	1.62264604	1	0.88337344	96.8100608
GOTERM_BP_FAT	GO:0050769~positive regulation of neurogenesis	3	0.13186813	0.19777013	189	59	13528	3.63949422	1	0.88942243	97.3580228
GOTERM_BP_FAT	GO:0050767~regulation of neurogenesis	5	0.21978022	0.20060304	189	166	13528	2.15592529	1	0.89156802	97.5077276
GOTERM_BP_FAT	GO:0000902~cell morphogenesis	8	0.35164835	0.2272326	189	356	13528	1.60846561	1	0.90976621	98.5744812
GOTERM_BP_FAT	GO:0032271~regulation of protein polymerization	3	0.13186813	0.24380606	189	68	13528	3.15779645	1	0.91464803	99.0029572
GOTERM_BP_FAT	GO:0010720~positive regulation of cell development	3	0.13186813	0.24896039	189	69	13528	3.11203129	1	0.91582013	99.1092947
GOTERM_BP_FAT	GO:0031099~regeneration	3	0.13186813	0.24896039	189	69	13528	3.11203129	1	0.91582013	99.1092947
GOTERM_BP_FAT	GO:0022604~regulation of cell morphogenesis	4	0.17582418	0.27428	189	131	13528	2.18554869	1	0.92654309	99.4940093
GOTERM_BP_FAT	GO:0044087~regulation of cellular component biogenesis	4	0.17582418	0.31600514	189	142	13528	2.01624562	1	0.94370356	99.8094133
GOTERM_BP_FAT	GO:0060284~regulation of cell development	5	0.21978022	0.31838133	189	205	13528	1.74577365	1	0.94431027	99.8200421
GOTERM_BP_FAT	GO:0030031~cell projection assembly	3	0.13186813	0.32103411	189	83	13528	2.58711035	1	0.94507702	99.8312494
GOTERM_BP_FAT	GO:0010638~positive regulation of organelle organization	3	0.13186813	0.32103411	189	83	13528	2.58711035	1	0.94507702	99.8312494
GOTERM_BP_FAT	GO:0051493~regulation of cytoskeleton organization	3	0.13186813	0.56645568	189	136	13528	1.57889823	1	0.99012739	99.9998965
GOTERM_BP_FAT	GO:0009611~response to wounding	7	0.30769231	0.75079808	189	530	13528	0.9453529	1	0.99883031	100
GOTERM_BP_FAT	GO:0045597~positive regulation of cell differentiation	3	0.13186813	0.83094265	189	229	13528	0.93768628	1	0.99976323	100
GOTERM_BP_FAT	GO:0051094~positive regulation of developmental process	3	0.13186813	0.90187776	189	278	13528	0.77241064	1	0.99997295	100

Annotation Cluster 26 Enrichment Score: 0.9649886105924698

Category	Term	Count	%	PValue	List Total	Pop Hits	Pop Total	Fold Enrichme	Bonferroni	Benjamini	FDR

UP_SEQ_FEATURE	repeat:WD 6	7	0.30769231	0.02587944	225	192	19113	3.09701389	1	0.84907005	34.6246123
UP_SEQ_FEATURE	repeat:WD 8	4	0.17582418	0.03334603	225	60	19113	5.66311111	1	0.87494111	42.2906192
UP_SEQ_FEATURE	repeat:WD 5	7	0.30769231	0.05714775	225	233	19113	2.55204578	1	0.92396795	61.4755442
UP_SEQ_FEATURE	repeat:WD 4	7	0.30769231	0.07578308	225	251	19113	2.36903054	1	0.94167595	72.1258304
INTERPRO	IPR015943:WD40/YVTN repeat-like	8	0.35164835	0.09283179	215	300	16659	2.06623256	1	0.87743544	75.1536116
UP_SEQ_FEATURE	repeat:WD 3	7	0.30769231	0.09288325	225	265	19113	2.24387421	1	0.96711469	79.4065722
UP_SEQ_FEATURE	repeat:WD 9	3	0.13186813	0.0939287	225	44	19113	5.79181818	1	0.96523511	79.787942
UP_SEQ_FEATURE	repeat:WD 7	5	0.21978022	0.10013323	225	150	19113	2.83155556	1	0.96676126	81.9183566
UP_SEQ_FEATURE	repeat:WD 2	7	0.30769231	0.10352139	225	274	19113	2.17017032	1	0.96190848	82.9908856
UP_SEQ_FEATURE	repeat:WD 1	7	0.30769231	0.10352139	225	274	19113	2.17017032	1	0.96190848	82.9908856
SP_PIR_KEYWORDS	wd repeat	7	0.30769231	0.10448868	225	276	19235	2.16819646	1	0.55609267	76.8871778
INTERPRO	IPR019775:WD40 repeat, conserved site	7	0.30769231	0.14724424	215	277	16659	1.95807237	1	0.93255335	89.7357326
INTERPRO	IPR019782:WD40 repeat 2	6	0.26373626	0.1663767	215	226	16659	2.05708994	1	0.93811244	92.5786953
INTERPRO	IPR017986:WD40 repeat, region	6	0.26373626	0.19073708	215	238	16659	1.95337112	1	0.94307918	95.1427206
INTERPRO	IPR019781:WD40 repeat, subgroup	6	0.26373626	0.20210303	215	242	16659	1.92108399	1	0.94017048	96.0317577
INTERPRO	IPR001680:WD40 repeat	6	0.26373626	0.26003798	215	266	16659	1.7477531	1	0.9665855	98.6488397
SMART	SM00320:WD40	6	0.26373626	0.47239754	155	266	9079	1.32122241	1	0.99256949	99.9460779

Annotation Cluster 27 Enrichment Score: 0.9610895569287055

Category	Term	Count	%	PValue	List Total	Pop Hits	Pop Total	Fold Enrichme	Bonferroni	Benjamini	FDR
GOTERM_BP_FAT	GO:0007264~small GTPase mediated signal transduction	10	0.43956044	0.02683417	189	305	13528	2.34677769	1	0.63526209	36.1433267
SP_PIR_KEYWORDS	lipoprotein	14	0.61538462	0.03781863	225	642	19235	1.86424368	0.99998434	0.3818758	40.0520816
INTERPRO	IPR005225:Small GTP-binding protein	6	0.26373626	0.06430263	215	167	16659	2.78384626	1	0.80949586	61.322384
GOTERM_MF_FAT	GO:0005525~GTP binding	10	0.43956044	0.06672032	177	372	12983	1.97178179	1	0.58298701	60.9817395
GOTERM_MF_FAT	GO:0032561~guanyl ribonucleotide binding	10	0.43956044	0.07594077	177	382	12983	1.92016446	1	0.58160021	65.9202532
GOTERM_MF_FAT	GO:0019001~guanyl nucleotide binding	10	0.43956044	0.07594077	177	382	12983	1.92016446	1	0.58160021	65.9202532
INTERPRO	IPR013753:Ras	5	0.21978022	0.077624	215	125	16659	3.09934884	1	0.85266042	68.4894608
SP_PIR_KEYWORDS	gtp-binding	8	0.35164835	0.09086291	225	329	19235	2.07875718	1	0.55251008	71.7577454
INTERPRO	IPR001806:Ras GTPase	5	0.21978022	0.09631401	215	135	16659	2.86976744	1	0.87595506	76.482493
UP_SEQ_FEATURE	short sequence motif:Effector region	4	0.17582418	0.10076744	225	95	19113	3.57670175	1	0.96452474	82.1238255
SP_PIR_KEYWORDS	prenylation	5	0.21978022	0.10959529	225	156	19235	2.74002849	1	0.5651996	78.5765996
UP_SEQ_FEATURE	lipid moiety-binding region:S-geranylgeranyl cysteine	4	0.17582418	0.11801613	225	102	19113	3.33124183	1	0.96733328	86.9404618
UP_SEQ_FEATURE	propeptide:Removed in mature form	6	0.26373626	0.13283983	225	229	19113	2.22567686	1	0.97760184	90.0779312
UP_SEQ_FEATURE	nucleotide phosphate-binding region:GTP	7	0.30769231	0.13428783	225	295	19113	2.01568362	1	0.97675221	90.3431123
SP_PIR_KEYWORDS	methylation	6	0.26373626	0.15280286	225	242	19235	2.11955923	1	0.64462589	88.9297269
GOTERM_MF_FAT	GO:0003924~GTPase activity	6	0.26373626	0.15897009	177	211	12983	2.08579002	1	0.78089406	90.5551008
GOTERM_CC_FAT	GO:0031225~anchored to membrane	5	0.21978022	0.25867139	151	220	12782	1.92384106	1	0.80722124	98.019175
SP_PIR_KEYWORDS	gpi-anchor	3	0.13186813	0.43261471	225	126	19235	2.03544974	1	0.89226174	99.9458881
KEGG_PATHWAY	hsa04670:Leukocyte transendothelial migration	3	0.13186813	0.50895617	74	118	5085	1.74702245	1	0.87653889	99.9627062

Annotation Cluster 28 Enrichment Score: 0.9408233114597319

Category	Term	Count	%	PValue	List Total	Pop Hits	Pop Total	Fold Enrichme	Bonferroni	Benjamini	FDR
GOTERM_BP_FAT	GO:0007155~cell adhesion	16	0.7032967	0.0634726	189	700	13528	1.6360393	1	0.73791626	66.0855232
GOTERM_BP_FAT	GO:0022610~biological adhesion	16	0.7032967	0.06409221	189	701	13528	1.63370544	1	0.73638954	66.4536226
SP_PIR_KEYWORDS	cell adhesion	7	0.30769231	0.36994615	225	422	19235	1.41806214	1	0.86993056	99.7826316

Annotation Cluster 29 Enrichment Score: 0.9110650959546635

Category	Term	Count	%	PValue	List Total	Pop Hits	Pop Total	Fold Enrichme	Bonferroni	Benjamini	FDR
GOTERM_MF_FAT	GO:0017137~Rab GTPase binding	3	0.13186813	0.06205567	177	30	12983	7.33502825	1	0.59865011	58.2381422
GOTERM_MF_FAT	GO:0017016~Ras GTPase binding	4	0.17582418	0.12610555	177	91	12983	3.22418824	1	0.74228113	84.074266
GOTERM_MF_FAT	GO:0031267~small GTPase binding	4	0.17582418	0.157465	177	101	12983	2.90496168	1	0.78606021	90.3221023
GOTERM_MF_FAT	GO:0051020~GTPase binding	4	0.17582418	0.18409412	177	109	12983	2.69175349	1	0.82440079	93.7529564

Annotation Cluster 30 Enrichment Score: 0.8626056114740914

Category	Term	Count	%	PValue	List Total	Pop Hits	Pop Total	Fold Enrichme	Bonferroni	Benjamini	FDR
UP_SEQ_FEATURE	DNA-binding region:Nuclear receptor	3	0.13186813	0.09756725	225	45	19113	5.66311111	1	0.96668307	81.0642065
UP_SEQ_FEATURE	zinc finger region:NR C4-type	3	0.13186813	0.09756725	225	45	19113	5.66311111	1	0.96668307	81.0642065
INTERPRO	IPR001628:Zinc finger, nuclear hormone receptor-type	3	0.13186813	0.11785069	215	46	16659	5.05328615	1	0.91596573	83.3400613

INTERPRO	IPR000536:Nuclear hormone receptor, ligand-binding, core	3	0.13186813	0.12637247	215	48	16659	4.84273256	1	0.9148208	85.4982549
INTERPRO	IPR008946:Nuclear hormone receptor, ligand-binding	3	0.13186813	0.12637247	215	48	16659	4.84273256	1	0.9148208	85.4982549
INTERPRO	IPR013088:Zinc finger, NHR/GATA-type	3	0.13186813	0.13941576	215	51	16659	4.55786594	1	0.9283425	88.3036601
GOTERM_MF_FAT	GO:0003707~steroid hormone receptor activity	3	0.13186813	0.1424582	177	49	12983	4.49083362	1	0.75841915	87.6892301
SMART	SM00399:ZnF_C4	3	0.13186813	0.18322735	155	46	9079	3.8200561	1	0.93876547	90.7636744
GOTERM_MF_FAT	GO:0004879~ligand-dependent nuclear receptor activity	3	0.13186813	0.18556176	177	58	12983	3.79398013	1	0.81952104	93.9043878
SMART	SM00430:HOLI	3	0.13186813	0.19553324	155	48	9079	3.6608871	1	0.93475366	92.2749062
Annotation Cluster 31	Enrichment Score: 0.8428066321758332										
Category	Term	Count	%	PValue	List Total	Pop Hits	Pop Total	Fold Enrichme	Bonferroni	Benjamini	FDR
GOTERM_BP_FAT	GO:0034613~cellular protein localization	12	0.52747253	0.02866618	189	411	13528	2.08983123	1	0.62308486	38.0969302
GOTERM_BP_FAT	GO:0070727~cellular macromolecule localization	12	0.52747253	0.03000486	189	414	13528	2.07468752	1	0.63147047	39.4888032
GOTERM_BP_FAT	GO:0046907~intracellular transport	15	0.65934066	0.07407528	189	657	13528	1.63417168	1	0.75544398	71.8908696
GOTERM_BP_FAT	GO:0006886~intracellular protein transport	10	0.43956044	0.07745951	189	374	13528	1.91381603	1	0.74976542	73.5378587
GOTERM_BP_FAT	GO:0008104~protein localization	17	0.74725275	0.16607187	189	882	13528	1.37959664	1	0.86665939	94.9945277
SP_PIR_KEYWORDS	protein transport	8	0.35164835	0.33498865	225	485	19235	1.410126	1	0.84868918	99.5548975
GOTERM_BP_FAT	GO:0015031~protein transport	13	0.57142857	0.37000172	189	762	13528	1.22112514	1	0.95887892	99.9508874
GOTERM_BP_FAT	GO:0045184~establishment of protein localization	13	0.57142857	0.3819496	189	769	13528	1.21000956	1	0.96099134	99.9641842
SP_PIR_KEYWORDS	transport	19	0.83516484	0.66972252	225	1670	19235	0.97262808	1	0.97520304	99.9999589
Annotation Cluster 32	Enrichment Score: 0.8327247961703117										
Category	Term	Count	%	PValue	List Total	Pop Hits	Pop Total	Fold Enrichme	Bonferroni	Benjamini	FDR
GOTERM_BP_FAT	GO:0007507~heart development	8	0.35164835	0.03076294	189	215	13528	2.6633198	1	0.62406863	40.2639168
GOTERM_BP_FAT	GO:0048705~skeletal system morphogenesis	5	0.21978022	0.07079006	189	112	13528	3.19538927	1	0.7575883	70.2003534
GOTERM_BP_FAT	GO:0060021~palate development	3	0.13186813	0.07261335	189	32	13528	6.71031746	1	0.75737234	71.1500282
GOTERM_BP_FAT	GO:0051216~cartilage development	4	0.17582418	0.0837937	189	74	13528	3.86901187	1	0.77363906	76.3796943
GOTERM_BP_FAT	GO:0043009~chordate embryonic development	9	0.3956044	0.0917138	189	331	13528	1.94619479	1	0.78566715	79.530339
GOTERM_BP_FAT	GO:0048568~embryonic organ development	6	0.26373626	0.09219799	189	172	13528	2.49686231	1	0.78004799	79.7095341
GOTERM_BP_FAT	GO:0009792~embryonic development ending in birth or egg ha	9	0.3956044	0.09462403	189	334	13528	1.928714	1	0.78543416	80.5854072
GOTERM_BP_FAT	GO:0048704~embryonic skeletal system morphogenesis	3	0.13186813	0.18768278	189	57	13528	3.76719577	1	0.88392699	96.7535023
GOTERM_BP_FAT	GO:0001701~in utero embryonic development	5	0.21978022	0.22988235	189	176	13528	2.03342953	1	0.90978328	98.6529765
GOTERM_BP_FAT	GO:0001568~blood vessel development	6	0.26373626	0.25355441	189	245	13528	1.75289925	1	0.91892848	99.1950027
GOTERM_BP_FAT	GO:0001501~skeletal system development	7	0.30769231	0.27776325	189	319	13528	1.57064902	1	0.92828178	99.5326014
GOTERM_BP_FAT	GO:0048706~embryonic skeletal system development	3	0.13186813	0.29023403	189	77	13528	2.78870336	1	0.93167767	99.6492856
GOTERM_BP_FAT	GO:0048598~embryonic morphogenesis	6	0.26373626	0.42391781	189	307	13528	1.39889354	1	0.96933462	99.9887671
GOTERM_BP_FAT	GO:0048562~embryonic organ morphogenesis	3	0.13186813	0.55432096	189	133	13528	1.61451247	1	0.98909593	99.9998369
Annotation Cluster 33	Enrichment Score: 0.8250251080858421										
Category	Term	Count	%	PValue	List Total	Pop Hits	Pop Total	Fold Enrichme	Bonferroni	Benjamini	FDR
GOTERM_BP_FAT	GO:0070647~protein modification by small protein conjugation	7	0.30769231	0.02435211	189	160	13528	3.13148148	1	0.64000713	33.4040199
GOTERM_BP_FAT	GO:0032446~protein modification by small protein conjugation	4	0.17582418	0.27805386	189	132	13528	2.1689915	1	0.92742927	99.535693
GOTERM_BP_FAT	GO:0016567~protein ubiquitination	3	0.13186813	0.49460577	189	119	13528	1.80445512	1	0.98087755	99.9987028
Annotation Cluster 34	Enrichment Score: 0.8200526203237678										
Category	Term	Count	%	PValue	List Total	Pop Hits	Pop Total	Fold Enrichme	Bonferroni	Benjamini	FDR
GOTERM_CC_FAT	GO:0000792~heterochromatin	3	0.13186813	0.08336776	151	41	12782	6.19382975	1	0.54726371	68.0353149
GOTERM_CC_FAT	GO:0000785~chromatin	6	0.26373626	0.08658018	151	200	12782	2.5394702	1	0.54928729	69.4723444
GOTERM_CC_FAT	GO:0000228~nuclear chromosome	5	0.21978022	0.12314096	151	162	12782	2.61262366	1	0.66180224	82.1248075
GOTERM_CC_FAT	GO:0000790~nuclear chromatin	3	0.13186813	0.13609334	151	55	12782	4.61721854	1	0.6682749	85.2914019
UP_SEQ_FEATURE	compositionally biased region:Gly-rich	6	0.26373626	0.14827989	225	238	19113	2.14151261	1	0.98046201	92.5847377
GOTERM_CC_FAT	GO:0044454~nuclear chromosome part	4	0.17582418	0.17270022	151	122	12782	2.77537727	1	0.74146835	91.6593997
GOTERM_CC_FAT	GO:0005694~chromosome	8	0.35164835	0.29570383	151	460	12782	1.47215664	1	0.83710419	98.9878395
GOTERM_CC_FAT	GO:0044427~chromosomal part	7	0.30769231	0.30039379	151	386	12782	1.53508561	1	0.83126873	99.0726771
Annotation Cluster 35	Enrichment Score: 0.8146794627012561										
Category	Term	Count	%	PValue	List Total	Pop Hits	Pop Total	Fold Enrichme	Bonferroni	Benjamini	FDR
GOTERM_MF_FAT	GO:0016563~transcription activator activity	13	0.57142857	0.01005157	177	410	12983	2.32574066	0.96841364	0.2501778	12.8634482
GOTERM_MF_FAT	GO:0003712~transcription cofactor activity	12	0.52747253	0.01061011	177	363	12983	2.42480273	0.973958	0.24468381	13.5311523

GOTERM_MF_FAT	GO:0008134~transcription factor binding	14	0.61538462	0.02246344	177	513	12983	2.0017621	0.99957783	0.42593174	26.6305584
GOTERM_MF_FAT	GO:0016564~transcription repressor activity	9	0.3956044	0.06609658	177	316	12983	2.08909032	1	0.59322277	60.6248085
SP_PIR_KEYWORDS	repressor	10	0.43956044	0.0685043	225	435	19235	1.96526181	1	0.48158881	61.0106334
GOTERM_MF_FAT	GO:0003713~transcription coactivator activity	7	0.30769231	0.0715336	177	214	12983	2.39930831	1	0.58326175	63.6369202
GOTERM_MF_FAT	GO:0003714~transcription corepressor activity	5	0.21978022	0.13442591	177	145	12983	2.52932009	1	0.75600949	86.0211508
GOTERM_BP_FAT	GO:0010629~negative regulation of gene expression	10	0.43956044	0.26798204	189	504	13528	1.42017301	1	0.92349483	99.4165254
GOTERM_BP_FAT	GO:0016481~negative regulation of transcription	9	0.3956044	0.30734583	189	459	13528	1.40346509	1	0.94083514	99.7654771
GOTERM_BP_FAT	GO:0000122~negative regulation of transcription from RNA pol	6	0.26373626	0.31146016	189	266	13528	1.61451247	1	0.9417048	99.787421
GOTERM_BP_FAT	GO:0010558~negative regulation of macromolecule biosyntheti	10	0.43956044	0.35044123	189	547	13528	1.30853235	1	0.95359523	99.9186871
GOTERM_BP_FAT	GO:0045892~negative regulation of transcription, DNA-depend	7	0.30769231	0.3749896	189	356	13528	1.40740741	1	0.9604544	99.9569207
GOTERM_BP_FAT	GO:0031327~negative regulation of cellular biosynthetic proces	10	0.43956044	0.37816494	189	561	13528	1.27587735	1	0.96059095	99.9603911
GOTERM_BP_FAT	GO:0051253~negative regulation of RNA metabolic process	7	0.30769231	0.3899466	189	362	13528	1.38408021	1	0.96196326	99.9711059
GOTERM_BP_FAT	GO:0009890~negative regulation of biosynthetic process	10	0.43956044	0.40207733	189	573	13528	1.24915741	1	0.96508417	99.9792522
GOTERM_BP_FAT	GO:0045934~negative regulation of nucleobase, nucleoside, nu	9	0.3956044	0.41885974	189	512	13528	1.25818452	1	0.9685835	99.9870254
GOTERM_BP_FAT	GO:0051172~negative regulation of nitrogen compound metab	9	0.3956044	0.43371845	189	519	13528	1.24121479	1	0.970687	99.9915353
GOTERM_BP_FAT	GO:0010605~negative regulation of macromolecule metabolic p	11	0.48351648	0.57201506	189	734	13528	1.07267563	1	0.99046523	99.9999164

Supplementary Table 8. GO annotation of ncRNA target ges in the cerebellum of healthy aged individuals.

Annotation Cluster 1 Enrichment Score: 4.3885760506107445

Category	Term	Count	%	PValue	List Total	Pop Hits	Pop Total	Fold Enrichme	Bonferroni	Benjamini	FDR
GOTERM_CC_FAT	GO:0070013~intracellular organelle lumen	34	2.27424749	2.62E-05	116	1779	12782	2.10592933	0.00624935	0.00156601	0.03382817
GOTERM_CC_FAT	GO:0043233~organelle lumen	34	2.27424749	4.19E-05	116	1820	12782	2.05848806	0.00995519	0.00199901	0.05398333
GOTERM_CC_FAT	GO:0031974~membrane-enclosed lumen	34	2.27424749	6.22E-05	116	1856	12782	2.01856049	0.01475214	0.00247394	0.08017904

Annotation Cluster 2 Enrichment Score: 4.373597197910588

Category	Term	Count	%	PValue	List Total	Pop Hits	Pop Total	Fold Enrichme	Bonferroni	Benjamini	FDR
GOTERM_BP_FAT	GO:0010557~positive regulation of macromolecule biosynthetic process	20	1.33779264	2.60E-05	136	654	13528	3.04191401	0.02909184	0.00268035	0.04178446
GOTERM_BP_FAT	GO:0031328~positive regulation of cellular biosynthetic process	20	1.33779264	4.89E-05	136	685	13528	2.90425075	0.05397248	0.00461296	0.07851155
GOTERM_BP_FAT	GO:0009891~positive regulation of biosynthetic process	20	1.33779264	5.94E-05	136	695	13528	2.86246297	0.06519125	0.00517222	0.09538449

Annotation Cluster 3 Enrichment Score: 3.348862688902971

Category	Term	Count	%	PValue	List Total	Pop Hits	Pop Total	Fold Enrichme	Bonferroni	Benjamini	FDR
GOTERM_BP_FAT	GO:0016481~negative regulation of transcription	15	1.00334448	1.97E-04	136	459	13528	3.25067282	0.20028938	0.01306132	0.31589324
GOTERM_BP_FAT	GO:0010629~negative regulation of gene expression	15	1.00334448	5.08E-04	136	504	13528	2.96043417	0.43780437	0.02583786	0.81193514
GOTERM_BP_FAT	GO:0045934~negative regulation of nucleobase, nucleoside, nucleotide	15	1.00334448	5.93E-04	136	512	13528	2.91417739	0.48977053	0.02655679	0.94802461
GOTERM_BP_FAT	GO:0051172~negative regulation of nitrogen compound metabolic proce	15	1.00334448	6.78E-04	136	519	13528	2.87487249	0.53645647	0.02913839	1.08248643

Annotation Cluster 4 Enrichment Score: 3.329583232953311

Category	Term	Count	%	PValue	List Total	Pop Hits	Pop Total	Fold Enrichme	Bonferroni	Benjamini	FDR
GOTERM_BP_FAT	GO:0010558~negative regulation of macromolecule biosynthetic proces	16	1.07023411	3.65E-04	136	547	13528	2.90956017	0.33866006	0.02046212	0.58362118
GOTERM_BP_FAT	GO:0031327~negative regulation of cellular biosynthetic process	16	1.07023411	4.75E-04	136	561	13528	2.83695082	0.41677036	0.02534817	0.76034749
GOTERM_BP_FAT	GO:0009890~negative regulation of biosynthetic process	16	1.07023411	5.92E-04	136	573	13528	2.77753824	0.48918732	0.0276016	0.94642277

Annotation Cluster 5 Enrichment Score: 2.855552456509324

Category	Term	Count	%	PValue	List Total	Pop Hits	Pop Total	Fold Enrichme	Bonferroni	Benjamini	FDR
UP_SEQ_FEATURE	chain:Teashirt homolog 2	3	0.2006689	2.00E-04	158	3	19113	120.968354	0.20855207	0.045701	0.321887
UP_SEQ_FEATURE	DNA-binding region:Homeobox; atypical	3	0.2006689	0.00181737	158	8	19113	45.3631329	0.88073813	0.261974	2.88862982
UP_SEQ_FEATURE	zinc finger region:C2H2-type 3; atypical	3	0.2006689	0.0074601	158	16	19113	22.6815665	0.9998421	0.58328564	11.3667205

Annotation Cluster 6 Enrichment Score: 2.7910667363785766

Category	Term	Count	%	PValue	List Total	Pop Hits	Pop Total	Fold Enrichme	Bonferroni	Benjamini	FDR
GOTERM_BP_FAT	GO:0006366~transcription from RNA polymerase II promoter	10	0.66889632	5.62E-04	136	234	13528	4.25087984	0.47145962	0.02734254	0.89857346
GOTERM_BP_FAT	GO:0006351~transcription, DNA-dependent	10	0.66889632	0.00262274	136	292	13528	3.40652699	0.94911012	0.09159727	4.12814562
GOTERM_BP_FAT	GO:0032774~RNA biosynthetic process	10	0.66889632	0.00287215	136	296	13528	3.36049285	0.96167702	0.09690569	4.51228325

Annotation Cluster 7 Enrichment Score: 2.0167702825222906

Category	Term	Count	%	PValue	List Total	Pop Hits	Pop Total	Fold Enrichme	Bonferroni	Benjamini	FDR
GOTERM_BP_FAT	GO:0043966~histone H3 acetylation	4	0.26755853	0.00213614	136	26	13528	15.3031674	0.91151989	0.07765191	3.3745364
GOTERM_BP_FAT	GO:0016573~histone acetylation	4	0.26755853	0.01211902	136	48	13528	8.28921569	0.99999901	0.29225609	17.776849
GOTERM_BP_FAT	GO:0006473~protein amino acid acetylation	4	0.26755853	0.01504603	136	52	13528	7.65158371	0.99999997	0.32955303	21.6016446
GOTERM_BP_FAT	GO:0043543~protein amino acid acylation	4	0.26755853	0.02199881	136	60	13528	6.63137255	1	0.40875443	30.0287742

Annotation Cluster 8 Enrichment Score: 1.9474336718295424

Category	Term	Count	%	PValue	List Total	Pop Hits	Pop Total	Fold Enrichme	Bonferroni	Benjamini	FDR
INTERPRO	IPR019787:Zinc finger, PHD-finger	5	0.33444816	0.00710751	150	85	16659	6.53294118	0.91026693	0.55230301	9.24883803
INTERPRO	IPR001965:Zinc finger, PHD-type	5	0.33444816	0.00866799	150	90	16659	6.17	0.94726893	0.52080019	11.1703153
INTERPRO	IPR019786:Zinc finger, PHD-type, conserved site	5	0.33444816	0.00970382	150	93	16659	5.97096774	0.96296563	0.4827256	12.4248918
SMART	SM00249:PHD	5	0.33444816	0.02714482	116	90	9079	4.34818008	0.95019725	0.63208194	26.6849716

Annotation Cluster 9 Enrichment Score: 1.7260176638262983

Category	Term	Count	%	PValue	List Total	Pop Hits	Pop Total	Fold Enrichme	Bonferroni	Benjamini	FDR
GOTERM_BP_FAT	GO:0016578~histone deubiquitination	3	0.2006689	0.00717465	136	13	13528	22.9547511	0.99971567	0.19336052	10.9158534
GOTERM_BP_FAT	GO:0016579~protein deubiquitination	3	0.2006689	0.0274759	136	26	13528	11.4773756	1	0.44904814	36.0609137
GOTERM_BP_FAT	GO:0070646~protein modification by small protein removal	3	0.2006689	0.03366622	136	29	13528	10.2900609	1	0.470932	42.2903489

Annotation Cluster 10 Enrichment Score: 1.719260666521722

Category	Term	Count	%	PValue	List Total	Pop Hits	Pop Total	Fold Enrichme	Bonferroni	Benjamini	FDR
INTERPRO	IPR002290:Serine/threonine protein kinase	9	0.60200669	0.00231941	150	259	16659	3.8592278	0.54382167	0.32459025	3.1100302
INTERPRO	IPR017442:Serine/threonine protein kinase-related	9	0.60200669	0.01553655	150	359	16659	2.78423398	0.99497121	0.58608561	19.1881976
SMART	SM00220:S_TKc	9	0.60200669	0.01689013	116	259	9079	2.71971109	0.84382046	0.60480443	17.4804238
INTERPRO	IPR000719:Protein kinase, core	9	0.60200669	0.06426078	150	476	16659	2.09987395	1	0.71268926	59.4923231
GOTERM_MF_FAT	GO:0004674~protein serine/threonine kinase activity	9	0.60200669	0.0647717	130	430	12983	2.09028623	1	0.59382791	59.0581588

Annotation Cluster 11 Enrichment Score: 1.6819655396756796

Category	Term	Count	%	PValue	List Total	Pop Hits	Pop Total	Fold Enrichme	Bonferroni	Benjamini	FDR
GOTERM_BP_FAT	GO:0030218~erythrocyte differentiation	4	0.26755853	0.0089644	136	43	13528	9.25307798	0.99996325	0.23036008	13.4591057
GOTERM_BP_FAT	GO:0034101~erythrocyte homeostasis	4	0.26755853	0.01281682	136	49	13528	8.12004802	0.99999956	0.30007762	18.7042517
GOTERM_BP_FAT	GO:0048872~homeostasis of number of cells	4	0.26755853	0.07830723	136	100	13528	3.97882353	1	0.68069441	72.9910925

Annotation Cluster 12 Enrichment Score: 1.6218458376806009

Category	Term	Count	%	PValue	List Total	Pop Hits	Pop Total	Fold Enrichme	Bonferroni	Benjamini	FDR
UP_SEQ_FEATURE	domain:SH3 3	3	0.2006689	0.01046078	158	19	19113	19.1002665	0.99999542	0.64099604	15.5873671
UP_SEQ_FEATURE	domain:SH3 1	3	0.2006689	0.03519925	158	36	19113	10.0806962	1	0.86395115	43.8657862
UP_SEQ_FEATURE	domain:SH3 2	3	0.2006689	0.03701391	158	37	19113	9.80824495	1	0.85294855	45.5431526

Annotation Cluster 13 Enrichment Score: 1.6004092795727134

Category	Term	Count	%	PValue	List Total	Pop Hits	Pop Total	Fold Enrichme	Bonferroni	Benjamini	FDR
UP_SEQ_FEATURE	domain:Helix-loop-helix motif	5	0.33444816	0.01455692	158	114	19113	5.30562958	0.99999996	0.73249693	21.0452002
INTERPRO	IPR001092:Basic helix-loop-helix dimerisation region bHLH	5	0.33444816	0.01922506	150	114	16659	4.87105263	0.99858602	0.60832969	23.2118657
SMART	SM00353:HLH	5	0.33444816	0.05647224	116	114	9079	3.43277375	0.99822883	0.7183874	48.0898125

Annotation Cluster 14 Enrichment Score: 1.5934526334197026

Category	Term	Count	%	PValue	List Total	Pop Hits	Pop Total	Fold Enrichme	Bonferroni	Benjamini	FDR
UP_SEQ_FEATURE	DNA-binding region:A.T hook 3	3	0.2006689	0.00232403	158	9	19113	40.3227848	0.93412198	0.28822527	3.67988637
SP_PIR_KEYWORDS	bromodomain	3	0.2006689	0.04028318	158	39	19235	9.36465433	0.99987716	0.32396169	40.7266858
INTERPRO	IPR001487:Bromodomain	3	0.2006689	0.04967826	150	40	16659	8.3295	0.99999997	0.68278602	50.0066939
SMART	SM00297:BROMO	3	0.2006689	0.0909197	116	40	9079	5.8700431	0.99996926	0.82301322	65.8755412

Annotation Cluster 15 Enrichment Score: 1.519698850745465

Category	Term	Count	%	PValue	List Total	Pop Hits	Pop Total	Fold Enrichme	Bonferroni	Benjamini	FDR
UP_SEQ_FEATURE	domain:HECT	3	0.2006689	0.02203728	158	28	19113	12.9608951	1	0.76477297	30.1681844
INTERPRO	IPR000569:HECT	3	0.2006689	0.02580035	150	28	16659	11.8992857	0.99985445	0.6685815	29.9277395
SMART	SM00119:HECTc	3	0.2006689	0.04854226	116	28	9079	8.38577586	0.99558982	0.74230025	42.9506714

Annotation Cluster 16 Enrichment Score: 1.3599811386091247

Category	Term	Count	%	PValue	List Total	Pop Hits	Pop Total	Fold Enrichme	Bonferroni	Benjamini	FDR
GOTERM_BP_FAT	GO:0030097~hemopoiesis	7	0.46822743	0.03096747	136	236	13528	2.9503988	1	0.46518303	39.6480122
GOTERM_BP_FAT	GO:0048534~hemopoietic or lymphoid organ development	7	0.46822743	0.04606821	136	260	13528	2.6780543	1	0.5393494	53.0976612
GOTERM_BP_FAT	GO:0002520~immune system development	7	0.46822743	0.05831087	136	276	13528	2.52280477	1	0.60175442	61.8809765

Annotation Cluster 17 Enrichment Score: 1.3464995094502206

Category	Term	Count	%	PValue	List Total	Pop Hits	Pop Total	Fold Enrichme	Bonferroni	Benjamini	FDR
UP_SEQ_FEATURE	domain:LDL-receptor class A 1	3	0.2006689	0.02057261	158	27	19113	13.4409283	1	0.76055322	28.4636916
UP_SEQ_FEATURE	domain:LDL-receptor class A 2	3	0.2006689	0.02057261	158	27	19113	13.4409283	1	0.76055322	28.4636916
INTERPRO	IPR002172:Low density lipoprotein-receptor, class A, cysteine-rich	3	0.2006689	0.07368224	150	50	16659	6.6636	1	0.72568859	64.7026709
SMART	SM00192:LDLa	3	0.2006689	0.13184397	116	50	9079	4.69603448	0.9999998	0.78585108	79.703033

Annotation Cluster 18 Enrichment Score: 1.3423180133629153

Category	Term	Count	%	PValue	List Total	Pop Hits	Pop Total	Fold Enrichme	Bonferroni	Benjamini	FDR
GOTERM_BP_FAT	GO:0006305~DNA alkylation	3	0.2006689	0.02363683	136	24	13528	12.4338235	1	0.41250227	31.8865112
GOTERM_BP_FAT	GO:0006306~DNA methylation	3	0.2006689	0.02363683	136	24	13528	12.4338235	1	0.41250227	31.8865112
GOTERM_BP_FAT	GO:0006304~DNA modification	3	0.2006689	0.04267113	136	33	13528	9.04278075	1	0.52728846	50.3433495

GOTERM_BP_FAT	GO:0040029~regulation of gene expression, epigenetic	3	0.2006689	0.17923193	136	77	13528	3.87547746	1	0.87430595	95.8024762

Annotation Cluster 19 Enrichment Score: 1.2961565918216877

Category	Term	Count	%	PValue	List Total	Pop Hits	Pop Total	Fold Enrichme	Bonferroni	Benjamini	FDR
GOTERM_BP_FAT	GO:0043414~biopolymer methylation	4	0.26755853	0.03158608	136	69	13528	5.76641091	1	0.46037982	40.2635195
GOTERM_BP_FAT	GO:0032259~methylation	4	0.26755853	0.04032311	136	76	13528	5.23529412	1	0.51774359	48.3517294
GOTERM_BP_FAT	GO:0006730~one-carbon metabolic process	4	0.26755853	0.10150339	136	112	13528	3.55252101	1	0.74430258	82.0606751

Annotation Cluster 20 Enrichment Score: 1.2811125633374898

Category	Term	Count	%	PValue	List Total	Pop Hits	Pop Total	Fold Enrichme	Bonferroni	Benjamini	FDR
GOTERM_BP_FAT	GO:0060415~muscle tissue morphogenesis	3	0.2006689	0.02948311	136	27	13528	11.0522876	1	0.4665852	38.14674
GOTERM_BP_FAT	GO:0055008~cardiac muscle tissue morphogenesis	3	0.2006689	0.02948311	136	27	13528	11.0522876	1	0.4665852	38.14674
GOTERM_BP_FAT	GO:0003007~heart morphogenesis	3	0.2006689	0.16501208	136	73	13528	4.08783239	1	0.87062411	94.4697687

Annotation Cluster 21 Enrichment Score: 1.2143057258828365

Category	Term	Count	%	PValue	List Total	Pop Hits	Pop Total	Fold Enrichme	Bonferroni	Benjamini	FDR
INTERPRO	IPR000504:RNA recognition motif, RNP-1	6	0.40133779	0.04122128	150	211	16659	3.15810427	0.99999934	0.69446012	43.6020468
INTERPRO	IPR012677:Nucleotide-binding, alpha-beta plait	6	0.40133779	0.04263602	150	213	16659	3.1284507	0.9999996	0.67789002	44.7238388
SMART	SM00360:RRM	6	0.40133779	0.12947465	116	211	9079	2.22560876	0.99999973	0.81349686	79.0694016

Annotation Cluster 22 Enrichment Score: 1.1800489278727175

Category	Term	Count	%	PValue	List Total	Pop Hits	Pop Total	Fold Enrichme	Bonferroni	Benjamini	FDR
KEGG_PATHWAY	hsa03022:Basal transcription factors	3	0.2006689	0.03787717	46	35	5085	9.47515528	0.95079824	0.45248488	33.5315243
GOTERM_BP_FAT	GO:0006368~RNA elongation from RNA polymerase II promoter	3	0.2006689	0.08284389	136	48	13528	6.21691177	1	0.68883978	75.0479001
GOTERM_BP_FAT	GO:0006354~RNA elongation	3	0.2006689	0.09187878	136	51	13528	5.85121107	1	0.71940334	78.7142594

Annotation Cluster 23 Enrichment Score: 1.1786280202655084

Category	Term	Count	%	PValue	List Total	Pop Hits	Pop Total	Fold Enrichme	Bonferroni	Benjamini	FDR
UP_SEQ_FEATURE	domain:Fibronectin type-III 6	3	0.2006689	0.02667963	158	31	19113	11.7066149	1	0.79414938	35.3224038
UP_SEQ_FEATURE	domain:Fibronectin type-III 5	3	0.2006689	0.05274318	158	45	19113	8.06455696	1	0.90424965	58.2361401
UP_SEQ_FEATURE	domain:Fibronectin type-III 4	3	0.2006689	0.09216122	158	62	19113	5.85330747	1	0.96041856	78.9451717
UP_SEQ_FEATURE	domain:Fibronectin type-III 3	3	0.2006689	0.14879679	158	83	19113	4.37235016	1	0.98747203	92.543171

Annotation Cluster 24 Enrichment Score: 1.1446056932535258

Category	Term	Count	%	PValue	List Total	Pop Hits	Pop Total	Fold Enrichme	Bonferroni	Benjamini	FDR
INTERPRO	IPR012680:Laminin G, subdomain 2	3	0.2006689	0.05421142	150	42	16659	7.93285714	0.99999999	0.69192984	53.1555675
INTERPRO	IPR001791:Laminin G	3	0.2006689	0.06127182	150	45	16659	7.404	1	0.71553574	57.6959718
SMART	SM00282:LamG	3	0.2006689	0.11087442	116	45	9079	5.21781609	0.99999727	0.83957108	73.4329205

Annotation Cluster 25 Enrichment Score: 1.1324784553550749

Category	Term	Count	%	PValue	List Total	Pop Hits	Pop Total	Fold Enrichme	Bonferroni	Benjamini	FDR
GOTERM_BP_FAT	GO:0045639~positive regulation of myeloid cell differentiation	4	0.26755853	0.00426046	136	33	13528	12.057041	0.9921062	0.12583929	6.6242572
GOTERM_BP_FAT	GO:0045637~regulation of myeloid cell differentiation	4	0.26755853	0.03276621	136	70	13528	5.68403361	1	0.46722408	41.4214532
GOTERM_BP_FAT	GO:0045597~positive regulation of cell differentiation	4	0.26755853	0.40105235	136	229	13528	1.73747752	1	0.97705054	99.9733034
GOTERM_BP_FAT	GO:0051094~positive regulation of developmental process	4	0.26755853	0.52723335	136	278	13528	1.43123149	1	0.99176746	99.9994014

Annotation Cluster 26 Enrichment Score: 1.084771657160871

Category	Term	Count	%	PValue	List Total	Pop Hits	Pop Total	Fold Enrichme	Bonferroni	Benjamini	FDR
UP_SEQ_FEATURE	domain:SET	3	0.2006689	0.06134749	158	49	19113	7.40622578	1	0.92207786	63.9469135
INTERPRO	IPR001214:SET	3	0.2006689	0.07113937	150	49	16659	6.79959184	1	0.73093497	63.3612659
SMART	SM00317:SET	3	0.2006689	0.12757872	116	49	9079	4.79187192	0.99999965	0.84426179	78.5494441

Annotation Cluster 27 Enrichment Score: 1.0190784578882492

Category	Term	Count	%	PValue	List Total	Pop Hits	Pop Total	Fold Enrichme	Bonferroni	Benjamini	FDR
SP_PIR_KEYWORDS	serine/threonine-protein kinase	8	0.53511706	0.0370893	158	381	19235	2.5562311	0.99974571	0.31355424	38.1680282
INTERPRO	IPR008271:Serine/threonine protein kinase, active site	8	0.53511706	0.04007297	150	354	16659	2.50983051	0.99999901	0.71540386	42.6760475
INTERPRO	IPR017441:Protein kinase, ATP binding site	8	0.53511706	0.11420873	150	455	16659	1.9527033	1	0.83173238	80.7956533
UP_SEQ_FEATURE	binding site:ATP	8	0.53511706	0.15921659	158	542	19113	1.78551077	1	0.99002313	93.8855367

UP_SEQ_FEATURE	active site:Proton acceptor	8	0.53511706	0.29704346	158	658	19113	1.47073987	1	0.9996379	99.6584912

Annotation Cluster 28 Enrichment Score: 1.0151164234425414

Category	Term	Count	%	PValue	List Total	Pop Hits	Pop Total	Fold Enrichme	Bonferroni	Benjamini	FDR
GOTERM_MF_FAT	GO:0046872~metal ion binding	50	3.34448161	0.08241714	130	4140	12983	1.20615013	1	0.65382799	68.2422219
GOTERM_MF_FAT	GO:0043169~cation binding	50	3.34448161	0.09436982	130	4179	12983	1.19489388	1	0.66267062	73.3368534
GOTERM_MF_FAT	GO:0043167~ion binding	50	3.34448161	0.1158244	130	4241	12983	1.1774255	1	0.6685128	80.6333002

Annotation Cluster 29 Enrichment Score: 0.9514999188577671

Category	Term	Count	%	PValue	List Total	Pop Hits	Pop Total	Fold Enrichme	Bonferroni	Benjamini	FDR
GOTERM_MF_FAT	GO:0004468~lysine N-acetyltransferase activity	3	0.2006689	0.05459167	130	38	12983	7.88441296	0.99999994	0.62373729	52.6991646
GOTERM_MF_FAT	GO:0004402~histone acetyltransferase activity	3	0.2006689	0.05459167	130	38	12983	7.88441296	0.99999994	0.62373729	52.6991646
GOTERM_MF_FAT	GO:0008080~N-acetyltransferase activity	3	0.2006689	0.14996292	130	69	12983	4.34214047	1	0.7469269	88.5446858
GOTERM_MF_FAT	GO:0016407~acetyltransferase activity	3	0.2006689	0.19595838	130	82	12983	3.65375235	1	0.77717504	94.5446058
GOTERM_MF_FAT	GO:0016410~N-acyltransferase activity	3	0.2006689	0.19957217	130	83	12983	3.60973123	1	0.77632268	94.8626767

Annotation Cluster 30 Enrichment Score: 0.9471442010718853

Category	Term	Count	%	PValue	List Total	Pop Hits	Pop Total	Fold Enrichme	Bonferroni	Benjamini	FDR
GOTERM_BP_FAT	GO:0006606~protein import into nucleus	4	0.26755853	0.05468919	136	86	13528	4.62653899	1	0.59273039	59.4582108
GOTERM_BP_FAT	GO:0051170~nuclear import	4	0.26755853	0.05781975	136	88	13528	4.52139037	1	0.60354756	61.56059
GOTERM_BP_FAT	GO:0034504~protein localization in nucleus	4	0.26755853	0.06770598	136	94	13528	4.23279099	1	0.63444794	67.5483282
GOTERM_BP_FAT	GO:0017038~protein import	4	0.26755853	0.14296656	136	131	13528	3.03726987	1	0.84143589	91.5972255
GOTERM_BP_FAT	GO:0033365~protein localization in organelle	4	0.26755853	0.17900767	136	146	13528	2.7252216	1	0.87636126	95.784027
GOTERM_BP_FAT	GO:0006913~nucleocytoplasmic transport	4	0.26755853	0.20428205	136	156	13528	2.5505279	1	0.90517625	97.447881
GOTERM_BP_FAT	GO:0051169~nuclear transport	4	0.26755853	0.20943513	136	158	13528	2.51824274	1	0.90936257	97.7006478

Annotation Cluster 31 Enrichment Score: 0.9033296195469345

Category	Term	Count	%	PValue	List Total	Pop Hits	Pop Total	Fold Enrichme	Bonferroni	Benjamini	FDR
UP_SEQ_FEATURE	domain:Fibronectin type-III 2	4	0.26755853	0.09181203	158	130	19113	3.72210321	1	0.96352597	78.8142915
UP_SEQ_FEATURE	domain:Fibronectin type-III 1	4	0.26755853	0.09342506	158	131	19113	3.69369021	1	0.95489886	79.4125579
INTERPRO	IPR008957:Fibronectin, type III-like fold	4	0.26755853	0.22732552	150	184	16659	2.41434783	1	0.92874533	97.0070497

Annotation Cluster 32 Enrichment Score: 0.8554371753259123

Category	Term	Count	%	PValue	List Total	Pop Hits	Pop Total	Fold Enrichme	Bonferroni	Benjamini	FDR
GOTERM_MF_FAT	GO:0005083~small GTPase regulator activity	7	0.46822743	0.05564588	130	274	12983	2.55140371	0.99999996	0.60996201	53.3977311
GOTERM_MF_FAT	GO:0030695~GTPase regulator activity	7	0.46822743	0.21380624	130	404	12983	1.73040746	1	0.79449762	95.9559406
GOTERM_MF_FAT	GO:0060589~nucleoside-triphosphatase regulator activity	7	0.46822743	0.22815768	130	413	12983	1.69269883	1	0.81108305	96.8368569

Annotation Cluster 33 Enrichment Score: 0.7328439435832075

Category	Term	Count	%	PValue	List Total	Pop Hits	Pop Total	Fold Enrichme	Bonferroni	Benjamini	FDR
SP_PIR_KEYWORDS	Homeobox	5	0.33444816	0.1364947	158	242	19235	2.51529972	1	0.60079032	84.5372392
INTERPRO	IPR017970:Homeobox, conserved site	5	0.33444816	0.1546514	150	232	16659	2.39353448	1	0.8588795	89.8313939
INTERPRO	IPR001356:Homeobox	5	0.33444816	0.15971632	150	235	16659	2.36297872	1	0.85922286	90.6297437
SMART	SM00389:HOX	5	0.33444816	0.3473804	116	235	9079	1.66526046	1	0.96394242	99.1880837

Annotation Cluster 34 Enrichment Score: 0.6807409669585378

Category	Term	Count	%	PValue	List Total	Pop Hits	Pop Total	Fold Enrichme	Bonferroni	Benjamini	FDR
GOTERM_BP_FAT	GO:0006886~intracellular protein transport	7	0.46822743	0.17100978	136	374	13528	1.86174898	1	0.87316104	95.0741036
GOTERM_BP_FAT	GO:0034613~cellular protein localization	7	0.46822743	0.22792238	136	411	13528	1.69414627	1	0.92023928	98.4273303
GOTERM_BP_FAT	GO:0070727~cellular macromolecule localization	7	0.46822743	0.23279261	136	414	13528	1.68186985	1	0.92165842	98.5792381

Annotation Cluster 35 Enrichment Score: 0.6438636545960656

Category	Term	Count	%	PValue	List Total	Pop Hits	Pop Total	Fold Enrichme	Bonferroni	Benjamini	FDR
KEGG_PATHWAY	hsa05211:Renal cell carcinoma	3	0.2006689	0.12687249	46	70	5085	4.73757764	0.99997465	0.65293898	76.1915503
KEGG_PATHWAY	hsa04662:B cell receptor signaling pathway	3	0.2006689	0.14189044	46	75	5085	4.42173913	0.99999345	0.66212378	80.1834477
KEGG_PATHWAY	hsa05210:Colorectal cancer	3	0.2006689	0.16980488	46	84	5085	3.94798137	0.9999995	0.67259819	86.0329814
KEGG_PATHWAY	hsa04660:T cell receptor signaling pathway	3	0.2006689	0.24766768	46	108	5085	3.07065217	1	0.6890938	95.0718247
KEGG_PATHWAY	hsa05200:Pathways in cancer	3	0.2006689	0.79715294	46	328	5085	1.0110684	1	0.99310766	99.9999953

Annotation Cluster 36 Enrichment Score: 0.6143225045548678

Category	Term	Count	%	PValue	List Total	Pop Hits	Pop Total	Fold Enrichme	Bonferroni	Benjamini	FDR
UP_SEQ_FEATURE	domain:PH	5	0.33444816	0.13000044	158	237	19113	2.55207499	1	0.98113943	89.3974999
INTERPRO	IPR001849:Pleckstrin homology	5	0.33444816	0.23616654	150	277	16659	2.00469314	1	0.93131384	97.4408307
SMART	SM00233:PH	5	0.33444816	0.4675944	116	277	9079	1.41276609	1	0.98243161	99.9182983

Annotation Cluster 37 Enrichment Score: 0.6117000816626719

Category	Term	Count	%	PValue	List Total	Pop Hits	Pop Total	Fold Enrichme	Bonferroni	Benjamini	FDR
UP_SEQ_FEATURE	domain:EGF-like 3	3	0.2006689	0.1264333	158	75	19113	4.83873418	1	0.98260649	88.6748625
UP_SEQ_FEATURE	domain:EGF-like 2	3	0.2006689	0.16315135	158	88	19113	4.12392117	1	0.9902155	94.3306845
UP_SEQ_FEATURE	domain:EGF-like 1	3	0.2006689	0.25893545	158	120	19113	3.02420886	1	0.99896033	99.2004287
INTERPRO	IPR006209:EGF	3	0.2006689	0.31449087	150	127	16659	2.62346457	1	0.95885549	99.4127503
INTERPRO	IPR000742:EGF-like, type 3	3	0.2006689	0.52029288	150	194	16659	1.71742268	1	0.99598416	99.9954353

Annotation Cluster 38 Enrichment Score: 0.5939199968274068

Category	Term	Count	%	PValue	List Total	Pop Hits	Pop Total	Fold Enrichme	Bonferroni	Benjamini	FDR
SP_PIR_KEYWORDS	extracellular matrix	5	0.33444816	0.13502763	158	241	19235	2.52573665	1	0.60715587	84.1997276
GOTERM_CC_FAT	GO:0005578~proteinaceous extracellular matrix	5	0.33444816	0.325184	116	320	12782	1.72171336	1	0.92655188	99.3738627
GOTERM_CC_FAT	GO:0031012~extracellular matrix	5	0.33444816	0.37643344	116	345	12782	1.59695152	1	0.94051262	99.7739511

Annotation Cluster 39 Enrichment Score: 0.5787546567361608

Category	Term	Count	%	PValue	List Total	Pop Hits	Pop Total	Fold Enrichme	Bonferroni	Benjamini	FDR
GOTERM_BP_FAT	GO:0043065~positive regulation of apoptosis	7	0.46822743	0.25930087	136	430	13528	1.61928865	1	0.93144889	99.1920602
GOTERM_BP_FAT	GO:0043068~positive regulation of programmed cell death	7	0.46822743	0.26436257	136	433	13528	1.60806956	1	0.93272063	99.2762754
GOTERM_BP_FAT	GO:0010942~positive regulation of cell death	7	0.46822743	0.26775161	136	435	13528	1.60067613	1	0.93263765	99.3279817

Annotation Cluster 40 Enrichment Score: 0.5654050195072716

Category	Term	Count	%	PValue	List Total	Pop Hits	Pop Total	Fold Enrichme	Bonferroni	Benjamini	FDR
GOTERM_BP_FAT	GO:0032271~regulation of protein polymerization	3	0.2006689	0.14754881	136	68	13528	4.3884083	1	0.84828318	92.2901174
GOTERM_BP_FAT	GO:0043254~regulation of protein complex assembly	3	0.2006689	0.22650645	136	90	13528	3.31568628	1	0.92055143	98.3803867
GOTERM_BP_FAT	GO:0051493~regulation of cytoskeleton organization	3	0.2006689	0.39426692	136	136	13528	2.19420415	1	0.97853139	99.9680116
GOTERM_BP_FAT	GO:0044087~regulation of cellular component biogenesis	3	0.2006689	0.41550219	136	142	13528	2.1014913	1	0.97932292	99.9819616

Annotation Cluster 41 Enrichment Score: 0.5600900077223663

Category	Term	Count	%	PValue	List Total	Pop Hits	Pop Total	Fold Enrichme	Bonferroni	Benjamini	FDR
SP_PIR_KEYWORDS	ubl conjugation pathway	9	0.60200669	0.05757713	158	509	19235	2.15258263	0.99999771	0.41790604	52.966723
GOTERM_BP_FAT	GO:0043632~modification-dependent macromolecule catabolic process	9	0.60200669	0.21498267	136	574	13528	1.55964337	1	0.9137712	97.9464206
GOTERM_BP_FAT	GO:0019941~modification-dependent protein catabolic process	9	0.60200669	0.21498267	136	574	13528	1.55964337	1	0.9137712	97.9464206
GOTERM_BP_FAT	GO:0051603~proteolysis involved in cellular protein catabolic process	9	0.60200669	0.25035601	136	600	13528	1.49205882	1	0.93132992	99.0203606
GOTERM_BP_FAT	GO:0044257~cellular protein catabolic process	9	0.60200669	0.25455852	136	603	13528	1.48463565	1	0.93189149	99.104897
GOTERM_BP_FAT	GO:0030163~protein catabolic process	9	0.60200669	0.28167659	136	622	13528	1.43928504	1	0.93917511	99.5062287
GOTERM_BP_FAT	GO:0044265~cellular macromolecule catabolic process	9	0.60200669	0.43733128	136	725	13528	1.2348073	1	0.98345054	99.9902086
GOTERM_BP_FAT	GO:0009057~macromolecule catabolic process	9	0.60200669	0.52183397	136	781	13528	1.14626798	1	0.99138079	99.9992817
GOTERM_BP_FAT	GO:0006508~proteolysis	9	0.60200669	0.83501392	136	1054	13528	0.84936935	1	0.99986143	100

Annotation Cluster 42 Enrichment Score: 0.5544466270595619

Category	Term	Count	%	PValue	List Total	Pop Hits	Pop Total	Fold Enrichme	Bonferroni	Benjamini	FDR
GOTERM_BP_FAT	GO:0050864~regulation of B cell activation	3	0.2006689	0.09187878	136	51	13528	5.85121107	1	0.71940334	78.7142594
GOTERM_BP_FAT	GO:0051249~regulation of lymphocyte activation	3	0.2006689	0.4361029	136	148	13528	2.01629571	1	0.98362023	99.9898598
GOTERM_BP_FAT	GO:0001817~regulation of cytokine production	3	0.2006689	0.5418211	136	181	13528	1.64868378	1	0.99287818	99.9996381

Annotation Cluster 43 Enrichment Score: 0.5527787309744794

Category	Term	Count	%	PValue	List Total	Pop Hits	Pop Total	Fold Enrichme	Bonferroni	Benjamini	FDR
UP_SEQ_FEATURE	domain:Cadherin 2	3	0.2006689	0.21347207	158	105	19113	3.4562387	1	0.99776242	97.9129382
UP_SEQ_FEATURE	domain:Cadherin 1	3	0.2006689	0.21347207	158	105	19113	3.4562387	1	0.99776242	97.9129382
INTERPRO	IPR002126:Cadherin	3	0.2006689	0.25161064	150	108	16659	3.085	1	0.93420422	98.061791
GOTERM_BP_FAT	GO:0007156~homophilic cell adhesion	3	0.2006689	0.37681447	136	131	13528	2.2779524	1	0.97648736	99.9495313

SMART	SM00112:CA	3	0.2006689	0.39862785	116	108	9079	2.17409004	1	0.97516249	99.6772178

Annotation Cluster 44 Enrichment Score: 0.5338165988066164

Category	Term	Count	%	PValue	List Total	Pop Hits	Pop Total	Fold Enrichme	Bonferroni	Benjamini	FDR
GOTERM_BP_FAT	GO:0042594~response to starvation	3	0.2006689	0.06569176	136	42	13528	7.10504202	1	0.63718735	66.4043481
GOTERM_BP_FAT	GO:0031667~response to nutrient levels	3	0.2006689	0.58796538	136	197	13528	1.51478053	1	0.99496775	99.9999342
GOTERM_BP_FAT	GO:0009991~response to extracellular stimulus	3	0.2006689	0.64816796	136	220	13528	1.35641711	1	0.99740106	99.9999948

Annotation Cluster 45 Enrichment Score: 0.5077112360743746

Category	Term	Count	%	PValue	List Total	Pop Hits	Pop Total	Fold Enrichme	Bonferroni	Benjamini	FDR
GOTERM_BP_FAT	GO:0006469~negative regulation of protein kinase activity	3	0.2006689	0.21549031	136	87	13528	3.43002028	1	0.91245082	97.9676347
GOTERM_BP_FAT	GO:0033673~negative regulation of kinase activity	3	0.2006689	0.22650645	136	90	13528	3.31568628	1	0.92055143	98.3803867
GOTERM_BP_FAT	GO:0051348~negative regulation of transferase activity	3	0.2006689	0.24864875	136	96	13528	3.10845588	1	0.93138595	98.9839252
GOTERM_BP_FAT	GO:0043086~negative regulation of catalytic activity	3	0.2006689	0.76746835	136	277	13528	1.07729879	1	0.99945737	100

Annotation Cluster 46 Enrichment Score: 0.47896148659074833

Category	Term	Count	%	PValue	List Total	Pop Hits	Pop Total	Fold Enrichme	Bonferroni	Benjamini	FDR
INTERPRO	IPR006210:EGF-like	4	0.26755853	0.26818301	150	201	16659	2.21014925	1	0.9377849	98.5708354
SP_PIR_KEYWORDS	egf-like domain	4	0.26755853	0.28991767	158	230	19235	2.1172262	1	0.81104037	98.7157853
SMART	SM00181:EGF	4	0.26755853	0.47033749	116	201	9079	1.55755704	1	0.97868614	99.9229225

Annotation Cluster 47 Enrichment Score: 0.45127672853497

Category	Term	Count	%	PValue	List Total	Pop Hits	Pop Total	Fold Enrichme	Bonferroni	Benjamini	FDR
INTERPRO	IPR018029:C2 membrane targeting protein	3	0.2006689	0.24498662	150	106	16659	3.14320755	1	0.93371877	97.8149031
INTERPRO	IPR000008:C2 calcium-dependent membrane targeting	3	0.2006689	0.34704018	150	137	16659	2.4319708	1	0.97021881	99.6970471
SMART	SM00239:C2	3	0.2006689	0.52077206	116	137	9079	1.7138812	1	0.98184674	99.9750675

Annotation Cluster 48 Enrichment Score: 0.3605113422215786

Category	Term	Count	%	PValue	List Total	Pop Hits	Pop Total	Fold Enrichme	Bonferroni	Benjamini	FDR
INTERPRO	IPR013069:BTB/POZ	3	0.2006689	0.31449087	150	127	16659	2.62346457	1	0.95885549	99.4127503
UP_SEQ_FEATURE	domain:BTB	3	0.2006689	0.32569712	158	142	19113	2.55566946	1	0.99983207	99.8253512
INTERPRO	IPR000210:BTB/POZ-like	3	0.2006689	0.47721523	150	179	16659	1.86134078	1	0.99459034	99.9852922
INTERPRO	IPR011333:BTB/POZ fold	3	0.2006689	0.48370661	150	181	16659	1.84077348	1	0.99446352	99.9875916
SMART	SM00225:BTB	3	0.2006689	0.66638469	116	179	9079	1.31174148	1	0.99664711	99.9995806

Annotation Cluster 49 Enrichment Score: 0.3396322111449854

Category	Term	Count	%	PValue	List Total	Pop Hits	Pop Total	Fold Enrichme	Bonferroni	Benjamini	FDR
INTERPRO	IPR013098:Immunoglobulin I-set	4	0.26755853	0.12655863	150	138	16659	3.21913044	1	0.82779775	84.1351483
INTERPRO	IPR003598:Immunoglobulin subtype 2	4	0.26755853	0.27787616	150	205	16659	2.16702439	1	0.94048388	98.8079527
SMART	SM00408:IGc2	4	0.26755853	0.48337119	116	205	9079	1.52716569	1	0.97737696	99.9418058
SP_PIR_KEYWORDS	Immunoglobulin domain	4	0.26755853	0.7416933	158	470	19235	1.03608942	1	0.98879636	99.9999967
INTERPRO	IPR007110:Immunoglobulin-like	4	0.26755853	0.82980973	150	501	16659	0.88670659	1	0.99999367	100
INTERPRO	IPR013783:Immunoglobulin-like fold	4	0.26755853	0.87615315	150	553	16659	0.80332731	1	0.99999903	100

Annotation Cluster 50 Enrichment Score: 0.31071624601456915

Category	Term	Count	%	PValue	List Total	Pop Hits	Pop Total	Fold Enrichme	Bonferroni	Benjamini	FDR
GOTERM_BP_FAT	GO:0042113~B cell activation	3	0.2006689	0.17565867	136	76	13528	3.92647059	1	0.87337951	95.4993122
GOTERM_BP_FAT	GO:0046649~lymphocyte activation	3	0.2006689	0.59348785	136	199	13528	1.49955661	1	0.9950899	99.999947
GOTERM_BP_FAT	GO:0045321~leukocyte activation	3	0.2006689	0.69908368	136	242	13528	1.23310647	1	0.99847382	99.9999996
GOTERM_BP_FAT	GO:0001775~cell activation	3	0.2006689	0.78437625	136	287	13528	1.03976225	1	0.99961889	100

Annotation Cluster 51 Enrichment Score: 0.2892471245402736

Category	Term	Count	%	PValue	List Total	Pop Hits	Pop Total	Fold Enrichme	Bonferroni	Benjamini	FDR
SP_PIR_KEYWORDS	Endocytosis	3	0.2006689	0.1847915	158	96	19235	3.80439082	1	0.70159462	92.5641182
GOTERM_BP_FAT	GO:0010324~membrane invagination	3	0.2006689	0.64816796	136	220	13528	1.35641711	1	0.99740106	99.9999948
GOTERM_BP_FAT	GO:0006897~endocytosis	3	0.2006689	0.64816796	136	220	13528	1.35641711	1	0.99740106	99.9999948
GOTERM_BP_FAT	GO:0016044~membrane organization	3	0.2006689	0.89733459	136	381	13528	0.78323298	1	0.99998051	100

Annotation Cluster 52 Enrichment Score: 0.2097563699883077

Category	Term	Count	%	PValue	List Total	Pop Hits	Pop Total	Fold Enrichme	Bonferroni	Benjamini	FDR
GOTERM_BP_FAT	GO:0006915~apoptosis	7	0.46822743	0.56001208	136	602	13528	1.15663475	1	0.99399709	99.9998111
GOTERM_BP_FAT	GO:0012501~programmed cell death	7	0.46822743	0.57469756	136	611	13528	1.13959757	1	0.99455178	99.9998905
GOTERM_BP_FAT	GO:0008219~cell death	7	0.46822743	0.72961565	136	719	13528	0.96842019	1	0.99892441	99.9999999

Annotation Cluster 53 Enrichment Score: 0.20165294250584961

Category	Term	Count	%	PValue	List Total	Pop Hits	Pop Total	Fold Enrichme	Bonferroni	Benjamini	FDR
UP_SEQ_FEATURE	repeat:ANK 1	3	0.2006689	0.56821864	158	231	19113	1.57101759	1	0.99999994	99.9998674
UP_SEQ_FEATURE	repeat:ANK 2	3	0.2006689	0.57675887	158	232	19113	1.56424596	1	0.99999993	99.9999039
SP_PIR_KEYWORDS	ank repeat	3	0.2006689	0.58654338	158	240	19235	1.52175633	1	0.9623088	99.9986788
INTERPRO	IPR002110:Ankyrin	3	0.2006689	0.6310429	150	238	16659	1.39991597	1	0.99910713	99.9998717
SMART	SM00248:ANK	3	0.2006689	0.80884724	116	238	9079	0.98656187	1	0.99960702	99.9999992

Annotation Cluster 54 Enrichment Score: 0.1831340408458223

Category	Term	Count	%	PValue	List Total	Pop Hits	Pop Total	Fold Enrichme	Bonferroni	Benjamini	FDR
GOTERM_BP_FAT	GO:0007067~mitosis	3	0.2006689	0.64816796	136	220	13528	1.35641711	1	0.99740106	99.9999948
GOTERM_BP_FAT	GO:0000280~nuclear division	3	0.2006689	0.64816796	136	220	13528	1.35641711	1	0.99740106	99.9999948
GOTERM_BP_FAT	GO:0000087~M phase of mitotic cell cycle	3	0.2006689	0.65790349	136	224	13528	1.33219538	1	0.99750162	99.9999967
GOTERM_BP_FAT	GO:0048285~organelle fission	3	0.2006689	0.6697713	136	229	13528	1.30310814	1	0.99775537	99.9999981

Annotation Cluster 55 Enrichment Score: 0.16970133480193675

Category	Term	Count	%	PValue	List Total	Pop Hits	Pop Total	Fold Enrichme	Bonferroni	Benjamini	FDR
GOTERM_BP_FAT	GO:0008380~RNA splicing	4	0.26755853	0.54170325	136	284	13528	1.4009942	1	0.99306193	99.9996366
GOTERM_BP_FAT	GO:0006397~mRNA processing	4	0.26755853	0.62505639	136	321	13528	1.23950889	1	0.99666997	99.9999855
GOTERM_BP_FAT	GO:0006396~RNA processing	4	0.26755853	0.91456664	136	547	13528	0.72739004	1	0.99999147	100

Annotation Cluster 56 Enrichment Score: 0.15120609172708968

Category	Term	Count	%	PValue	List Total	Pop Hits	Pop Total	Fold Enrichme	Bonferroni	Benjamini	FDR
GOTERM_BP_FAT	GO:0042981~regulation of apoptosis	8	0.53511706	0.69823858	136	804	13528	0.9897571	1	0.99849776	99.9999996
GOTERM_BP_FAT	GO:0043067~regulation of programmed cell death	8	0.53511706	0.70807212	136	812	13528	0.9800058	1	0.99862048	99.9999997
GOTERM_BP_FAT	GO:0010941~regulation of cell death	8	0.53511706	0.71170561	136	815	13528	0.97639841	1	0.99866894	99.9999998

Annotation Cluster 57 Enrichment Score: 0.06744244072370521

Category	Term	Count	%	PValue	List Total	Pop Hits	Pop Total	Fold Enrichme	Bonferroni	Benjamini	FDR
GOTERM_MF_FAT	GO:0005524~ATP binding	13	0.86956522	0.81002902	130	1477	12983	0.87901151	1	0.99975945	100
GOTERM_MF_FAT	GO:0032559~adenyl ribonucleotide binding	13	0.86956522	0.82421257	130	1497	12983	0.86726787	1	0.99981152	100
GOTERM_MF_FAT	GO:0030554~adenyl nucleotide binding	13	0.86956522	0.87321724	130	1577	12983	0.82327204	1	0.99994778	100
GOTERM_MF_FAT	GO:0001883~purine nucleoside binding	13	0.86956522	0.88563128	130	1601	12983	0.81093067	1	0.99996237	100
GOTERM_MF_FAT	GO:0001882~nucleoside binding	13	0.86956522	0.89098981	130	1612	12983	0.80539702	1	0.99995862	100

Annotation Cluster 58 Enrichment Score: 0.04583113053512487

Category	Term	Count	%	PValue	List Total	Pop Hits	Pop Total	Fold Enrichme	Bonferroni	Benjamini	FDR
GOTERM_MF_FAT	GO:0032555~purine ribonucleotide binding	15	1.00334448	0.88899768	130	1836	12983	0.81592509	1	0.99996157	100
GOTERM_MF_FAT	GO:0032553~ribonucleotide binding	15	1.00334448	0.88899768	130	1836	12983	0.81592509	1	0.99996157	100
GOTERM_MF_FAT	GO:0017076~purine nucleotide binding	15	1.00334448	0.92194572	130	1918	12983	0.78104195	1	0.99998922	100

Supplementary Table 9. GO annotation of ncRNA target genes in the frontal cortex of healthy aged individuals

Annotation Cluster 1 Enrichment Score: 3.1014089792108255

Category	Term	Count	%	PValue	List Total	Pop Hits	Pop Total	Fold Enrichme	Bonferroni	Benjamini	FDR
GOTERM_CC_FAT	GO:0070013~intracellular organelle lumen	34	1.465517241	5.39E-04	134	1779	12782	1.8230433	0.13175985	0.06820595	0.7032352
GOTERM_CC_FAT	GO:0043233~organelle lumen	34	1.465517241	8.09E-04	134	1820	12782	1.78197474	0.19104909	0.06823286	1.05342436
GOTERM_CC_FAT	GO:0031974~membrane-enclosed lumen	34	1.465517241	0.00113814	134	1856	12782	1.74741058	0.2579677	0.07187664	1.47925241

Annotation Cluster 2 Enrichment Score: 2.840276393128198

Category	Term	Count	%	PValue	List Total	Pop Hits	Pop Total	Fold Enrichme	Bonferroni	Benjamini	FDR
GOTERM_BP_FAT	GO:0045893~positive regulation of transcription, DNA-depende	17	0.732758621	1.69E-04	161	477	13528	2.99459614	0.18303153	0.03313119	0.27257303
GOTERM_BP_FAT	GO:0051254~positive regulation of RNA metabolic process	17	0.732758621	1.86E-04	161	481	13528	2.96969306	0.19932944	0.03125897	0.29970254
GOTERM_BP_FAT	GO:0045941~positive regulation of transcription	17	0.732758621	0.00105697	161	564	13528	2.53266376	0.71800309	0.08645122	1.69461387
GOTERM_BP_FAT	GO:0010557~positive regulation of macromolecule biosynthetic	17	0.732758621	0.00465342	161	654	13528	2.18413205	0.99623927	0.20014572	7.26112516
GOTERM_BP_FAT	GO:0031328~positive regulation of cellular biosynthetic process	17	0.732758621	0.00718203	161	685	13528	2.08528812	0.99982096	0.25733803	10.9963058
GOTERM_BP_FAT	GO:0009891~positive regulation of biosynthetic process	17	0.732758621	0.00820146	161	695	13528	2.05528397	0.99994765	0.27238841	12.4618605

Annotation Cluster 3 Enrichment Score: 2.682249632124902

Category	Term	Count	%	PValue	List Total	Pop Hits	Pop Total	Fold Enrichme	Bonferroni	Benjamini	FDR
GOTERM_BP_FAT	GO:0006305~DNA alkylation	5	0.215517241	1.67E-04	161	24	13528	17.505176	0.18071632	0.03908082	0.2687625
GOTERM_BP_FAT	GO:0006306~DNA methylation	5	0.215517241	1.67E-04	161	24	13528	17.505176	0.18071632	0.03908082	0.2687625
GOTERM_BP_FAT	GO:0006304~DNA modification	5	0.215517241	5.90E-04	161	33	13528	12.7310371	0.50676451	0.05719648	0.94972557
GOTERM_BP_FAT	GO:0043414~biopolymer methylation	5	0.215517241	0.00898042	161	69	13528	6.08875686	0.99997956	0.27210023	13.5664301
GOTERM_BP_FAT	GO:0032259~methylation	5	0.215517241	0.01250989	161	76	13528	5.52795031	0.99999971	0.32048716	18.4093599
GOTERM_BP_FAT	GO:0006730~one-carbon metabolic process	5	0.215517241	0.04385658	161	112	13528	3.75110914	1	0.62994974	51.5582004

Annotation Cluster 4 Enrichment Score: 2.29708892754124

Category	Term	Count	%	PValue	List Total	Pop Hits	Pop Total	Fold Enrichme	Bonferroni	Benjamini	FDR
GOTERM_BP_FAT	GO:0006366~transcription from RNA polymerase II promoter	10	0.431034483	0.00186877	161	234	13528	3.59080533	0.89343611	0.12340176	2.97782797
GOTERM_BP_FAT	GO:0006351~transcription, DNA-dependent	10	0.431034483	0.00794761	161	292	13528	2.87756318	0.99992889	0.27267123	12.0990589
GOTERM_BP_FAT	GO:0032774~RNA biosynthetic process	10	0.431034483	0.00864849	161	296	13528	2.83867719	0.99996948	0.27026141	13.0973653

Annotation Cluster 5 Enrichment Score: 2.0573366445653756

Category	Term	Count	%	PValue	List Total	Pop Hits	Pop Total	Fold Enrichme	Bonferroni	Benjamini	FDR
GOTERM_BP_FAT	GO:0060351~cartilage development involved in endochondral b	3	0.129310345	0.00202121	161	6	13528	42.0124224	0.91124137	0.12588765	3.21702728
GOTERM_BP_FAT	GO:0001958~endochondral ossification	3	0.129310345	0.0084882	161	12	13528	21.0062112	0.99996297	0.27302782	12.8699943
GOTERM_BP_FAT	GO:0060350~endochondral bone morphogenesis	3	0.129310345	0.01496299	161	16	13528	15.7546584	0.99999999	0.34927489	21.6241309
GOTERM_BP_FAT	GO:0060349~bone morphogenesis	3	0.129310345	0.02297249	161	20	13528	12.6037267	1	0.44671955	31.312588

Annotation Cluster 6 Enrichment Score: 2.01938434207291

Category	Term	Count	%	PValue	List Total	Pop Hits	Pop Total	Fold Enrichme	Bonferroni	Benjamini	FDR
SP_PIR_KEYWORDS	trimer	4	0.172413793	0.00214948	193	26	19235	15.3328019	0.42971396	0.03449218	2.77507843
SP_PIR_KEYWORDS	triple helix	4	0.172413793	0.00358271	193	31	19235	12.8597693	0.60810742	0.04575839	4.58574629
SP_PIR_KEYWORDS	hydroxylysine	4	0.172413793	0.00358271	193	31	19235	12.8597693	0.60810742	0.04575839	4.58574629
GOTERM_BP_FAT	GO:0030199~collagen fibril organization	4	0.172413793	0.00473442	161	29	13528	11.5896338	0.99658836	0.19626303	7.38302922
GOTERM_CC_FAT	GO:0005581~collagen	4	0.172413793	0.00565242	134	35	12782	10.9014925	0.77352849	0.15211912	7.14969757
SP_PIR_KEYWORDS	hydroxyproline	4	0.172413793	0.00592792	193	37	19235	10.7744013	0.78813167	0.06810574	7.48149616
SP_PIR_KEYWORDS	hydroxylation	4	0.172413793	0.03411461	193	71	19235	5.61482887	0.9998837	0.28504163	36.4898826
INTERPRO	IPR008160:Collagen triple helix repeat	4	0.172413793	0.06433753	183	84	16659	4.33489461	1	0.83321999	60.501033
SP_PIR_KEYWORDS	collagen	4	0.172413793	0.06965865	193	95	19235	4.19634579	0.99999999	0.43507694	61.1066656

Annotation Cluster 7 Enrichment Score: 1.9308406068504038

Category	Term	Count	%	PValue	List Total	Pop Hits	Pop Total	Fold Enrichme	Bonferroni	Benjamini	FDR
UP_SEQ_FEATURE	region of interest:Nonhelical region (C-terminal)	3	0.129310345	5.94E-04	193	4	19113	74.2733161	0.49678276	0.10814842	0.9520956
UP_SEQ_FEATURE	propeptide:N-terminal propeptide	3	0.129310345	0.0014664	193	6	19113	49.515544	0.81638884	0.17165547	2.33348028
UP_SEQ_FEATURE	domain:Fibrillar collagen NC1	3	0.129310345	0.00520179	193	11	19113	27.0084786	0.99757943	0.33074046	8.04897887
INTERPRO	IPR000885:Fibrillar collagen, C-terminal	3	0.129310345	0.00611897	183	11	16659	24.8271237	0.91622824	0.46200975	8.21616064
GOTERM_CC_FAT	GO:0005583~fibrillar collagen	3	0.129310345	0.00662421	134	12	12782	23.8470149	0.82471077	0.15981384	8.33025207

SMART	SM00038:COLFI	3	0.129310345	0.00938922	125	11	9079	19.8087273	0.63898147	0.39915182	10.0775068
UP_SEQ_FEATURE	region of interest:Triple-helical region	3	0.129310345	0.02211359	193	23	19113	12.9170984	1	0.67468114	30.2181253
GOTERM_BP_FAT	GO:0007605~sensory perception of sound	3	0.129310345	0.31872331	161	97	13528	2.59870654	1	0.95883749	99.7975891
GOTERM_BP_FAT	GO:0050954~sensory perception of mechanical stimulus	3	0.129310345	0.34469633	161	103	13528	2.44732557	1	0.96647404	99.8920033

Annotation Cluster 8 Enrichment Score: 1.789204747326519

Category	Term	Count	%	PValue	List Total	Pop Hits	Pop Total	Fold Enrichme	Bonferroni	Benjamini	FDR
UP_SEQ_FEATURE	DNA-binding region:A.T hook 2	3	0.129310345	0.00620108	193	12	19113	24.757772	0.99924178	0.32910401	9.52391091
UP_SEQ_FEATURE	DNA-binding region:A.T hook 1	3	0.129310345	0.00620108	193	12	19113	24.757772	0.99924178	0.32910401	9.52391091
INTERPRO	IPR017956:AT hook, DNA-binding, conserved site	3	0.129310345	0.03484253	183	27	16659	10.1147541	0.9999994	0.79646957	39.0654747
SMART	SM00384:AT_hook	3	0.129310345	0.05201605	125	27	9079	8.07022222	0.99687747	0.76361138	45.200196

Annotation Cluster 9 Enrichment Score: 1.6108641927962983

Category	Term	Count	%	PValue	List Total	Pop Hits	Pop Total	Fold Enrichme	Bonferroni	Benjamini	FDR
GOTERM_BP_FAT	GO:0048738~cardiac muscle tissue development	5	0.215517241	0.00457565	161	57	13528	7.37060041	0.99587059	0.20446007	7.14394935
GOTERM_BP_FAT	GO:0014706~striated muscle tissue development	5	0.215517241	0.05269277	161	119	13528	3.53045566	1	0.67279505	58.3079252
GOTERM_BP_FAT	GO:0060537~muscle tissue development	5	0.215517241	0.06098228	161	125	13528	3.36099379	1	0.70322465	63.8287544

Annotation Cluster 10 Enrichment Score: 1.5888584571331572

Category	Term	Count	%	PValue	List Total	Pop Hits	Pop Total	Fold Enrichme	Bonferroni	Benjamini	FDR
GOTERM_BP_FAT	GO:0016578~histone deubiquitination	3	0.129310345	0.00995412	161	13	13528	19.3903488	0.9999937	0.28974886	14.9287488
GOTERM_BP_FAT	GO:0016579~protein deubiquitination	3	0.129310345	0.03752852	161	26	13528	9.69517439	1	0.5854265	46.1084543
GOTERM_BP_FAT	GO:0070646~protein modification by small protein removal	3	0.129310345	0.04582054	161	29	13528	8.69222532	1	0.6330621	53.1415117

Annotation Cluster 11 Enrichment Score: 1.1895869455621284

Category	Term	Count	%	PValue	List Total	Pop Hits	Pop Total	Fold Enrichme	Bonferroni	Benjamini	FDR
INTERPRO	IPR008271:Serine/threonine protein kinase, active site	9	0.387931034	0.04090905	183	354	16659	2.31439289	0.99999995	0.81501535	44.2027255
INTERPRO	IPR017442:Serine/threonine protein kinase-related	9	0.387931034	0.04368925	183	359	16659	2.282159	0.99999999	0.80615455	46.4200346
INTERPRO	IPR000719:Protein kinase, core	9	0.387931034	0.15102375	183	476	16659	1.72120816	1	0.95713669	89.8423129

Annotation Cluster 12 Enrichment Score: 1.1478057534477824

Category	Term	Count	%	PValue	List Total	Pop Hits	Pop Total	Fold Enrichme	Bonferroni	Benjamini	FDR
GOTERM_BP_FAT	GO:0016481~negative regulation of transcription	11	0.474137931	0.04611307	161	459	13528	2.0136673	1	0.62894792	53.3731434
GOTERM_BP_FAT	GO:0010629~negative regulation of gene expression	11	0.474137931	0.07616365	161	504	13528	1.83387558	1	0.73699649	72.2054704
GOTERM_BP_FAT	GO:0045934~negative regulation of nucleobase, nucleoside, nu	11	0.474137931	0.0825518	161	512	13528	1.80522127	1	0.75184026	75.1540104
GOTERM_BP_FAT	GO:0051172~negative regulation of nitrogen compound metabo	11	0.474137931	0.08840537	161	519	13528	1.78087339	1	0.76280798	77.5957574

Annotation Cluster 13 Enrichment Score: 1.125207457116569

Category	Term	Count	%	PValue	List Total	Pop Hits	Pop Total	Fold Enrichme	Bonferroni	Benjamini	FDR
SP_PIR_KEYWORDS	bromodomain	3	0.129310345	0.05772374	193	39	19235	7.66640096	0.99999982	0.38426855	54.0506773
INTERPRO	IPR001487:Bromodomain	3	0.129310345	0.07066421	183	40	16659	6.82745902	1	0.82476021	64.0724448
SMART	SM00297:BROMO	3	0.129310345	0.10323421	125	40	9079	5.4474	0.99999225	0.85932318	70.6798808

Annotation Cluster 14 Enrichment Score: 1.1146450588826677

Category	Term	Count	%	PValue	List Total	Pop Hits	Pop Total	Fold Enrichme	Bonferroni	Benjamini	FDR
KEGG_PATHWAY	hsa04512:ECM-receptor interaction	4	0.172413793	0.04889504	51	84	5085	4.74789916	0.97996363	0.85845012	41.1556486
GOTERM_BP_FAT	GO:0048706~embryonic skeletal system development	4	0.172413793	0.06302494	161	77	13528	4.364927	1	0.70404488	65.0796586
GOTERM_BP_FAT	GO:0048705~skeletal system morphogenesis	4	0.172413793	0.14698995	161	112	13528	3.00088731	1	0.87646743	92.3421754

Annotation Cluster 15 Enrichment Score: 1.0899566754835917

Category	Term	Count	%	PValue	List Total	Pop Hits	Pop Total	Fold Enrichme	Bonferroni	Benjamini	FDR
GOTERM_MF_FAT	GO:0018024~histone-lysine N-methyltransferase activity	3	0.129310345	0.05061956	148	32	12983	8.22402872	0.99999991	0.63802755	50.2738892
GOTERM_MF_FAT	GO:0016279~protein-lysine N-methyltransferase activity	3	0.129310345	0.05061956	148	32	12983	8.22402872	0.99999991	0.63802755	50.2738892
GOTERM_MF_FAT	GO:0016278~lysine N-methyltransferase activity	3	0.129310345	0.05061956	148	32	12983	8.22402872	0.99999991	0.63802755	50.2738892
GOTERM_MF_FAT	GO:0042054~histone methyltransferase activity	3	0.129310345	0.07188088	148	39	12983	6.747921	1	0.74676292	63.331912
UP_SEQ_FEATURE	domain:SET	3	0.129310345	0.08687912	193	49	19113	6.06312784	1	0.97321224	76.8309316
INTERPRO	IPR001214:SET	3	0.129310345	0.10007235	183	49	16659	5.57343593	1	0.90619738	77.0723238
GOTERM_MF_FAT	GO:0008276~protein methyltransferase activity	3	0.129310345	0.10979878	148	50	12983	5.26337838	1	0.80885864	79.0760073

GOTERM_MF_FAT	GO:0008170~N-methyltransferase activity	3	0.129310345	0.12089596	148	53	12983	4.9654513	1	0.81370899	82.3244398
SMART	SM00317:SET	3	0.129310345	0.14407305	125	49	9079	4.44685714	0.99999995	0.84538899	82.6524936
Annotation Cluster 16	Enrichment Score: 1.0769088105963525										
Category	Term	Count	%	PValue	List Total	Pop Hits	Pop Total	Fold Enrichme	Bonferroni	Benjamini	FDR
GOTERM_CC_FAT	GO:0044420~extracellular matrix part	6	0.25862069	0.00748699	134	117	12782	4.89169537	0.8603995	0.15132662	9.36673447
SP_PIR_KEYWORDS	extracellular matrix	6	0.25862069	0.09425023	193	241	19235	2.4812418	1	0.50256795	72.6023253
GOTERM_CC_FAT	GO:0005578~proteinaceous extracellular matrix	6	0.25862069	0.24058083	134	320	12782	1.78852612	1	0.87255609	97.2716928
GOTERM_CC_FAT	GO:0031012~extracellular matrix	6	0.25862069	0.29007767	134	345	12782	1.65892278	1	0.90578113	98.8706333
Annotation Cluster 17	Enrichment Score: 1.0223831719831173										
Category	Term	Count	%	PValue	List Total	Pop Hits	Pop Total	Fold Enrichme	Bonferroni	Benjamini	FDR
GOTERM_BP_FAT	GO:0010558~negative regulation of macromolecule biosyntheti	12	0.517241379	0.06005001	161	547	13528	1.84332383	1	0.70335583	63.2439791
GOTERM_BP_FAT	GO:0031327~negative regulation of cellular biosynthetic proces	12	0.517241379	0.06921881	161	561	13528	1.79732288	1	0.72238597	68.6291769
GOTERM_BP_FAT	GO:0009890~negative regulation of biosynthetic process	12	0.517241379	0.07776437	161	573	13528	1.75968261	1	0.73484279	72.9736624
GOTERM_BP_FAT	GO:0010605~negative regulation of macromolecule metabolic p	12	0.517241379	0.2517384	161	734	13528	1.37370318	1	0.94983948	99.0784972
Annotation Cluster 18	Enrichment Score: 0.9806145345725535										
Category	Term	Count	%	PValue	List Total	Pop Hits	Pop Total	Fold Enrichme	Bonferroni	Benjamini	FDR
GOTERM_MF_FAT	GO:0046872~metal ion binding	56	2.413793103	0.0886066	148	4140	12983	1.18659094	1	0.76590236	71.287779
GOTERM_MF_FAT	GO:0043169~cation binding	56	2.413793103	0.10209154	148	4179	12983	1.17551723	1	0.79912076	76.5038016
GOTERM_MF_FAT	GO:0043167~ion binding	56	2.413793103	0.12638647	148	4241	12983	1.15833211	1	0.81578786	83.7528086
Annotation Cluster 19	Enrichment Score: 0.9637991768681051										
Category	Term	Count	%	PValue	List Total	Pop Hits	Pop Total	Fold Enrichme	Bonferroni	Benjamini	FDR
GOTERM_BP_FAT	GO:0002062~chondrocyte differentiation	3	0.129310345	0.01878585	161	18	13528	14.0041408	1	0.3895082	26.3980187
GOTERM_BP_FAT	GO:0048704~embryonic skeletal system morphogenesis	3	0.129310345	0.14595309	161	57	13528	4.42236025	1	0.880197	92.1903435
GOTERM_BP_FAT	GO:0048562~embryonic organ morphogenesis	3	0.129310345	0.46833688	161	133	13528	1.89529725	1	0.98431326	99.9963201
Annotation Cluster 20	Enrichment Score: 0.9362469040850514										
Category	Term	Count	%	PValue	List Total	Pop Hits	Pop Total	Fold Enrichme	Bonferroni	Benjamini	FDR
GOTERM_BP_FAT	GO:0070271~protein complex biogenesis	11	0.474137931	0.07694461	161	505	13528	1.83024414	1	0.73581292	72.5827782
GOTERM_BP_FAT	GO:0006461~protein complex assembly	11	0.474137931	0.07694461	161	505	13528	1.83024414	1	0.73581292	72.5827782
GOTERM_BP_FAT	GO:0065003~macromolecular complex assembly	11	0.474137931	0.2623632	161	665	13528	1.38988465	1	0.95315623	99.2686613
Annotation Cluster 21	Enrichment Score: 0.8794686484431335										
Category	Term	Count	%	PValue	List Total	Pop Hits	Pop Total	Fold Enrichme	Bonferroni	Benjamini	FDR
INTERPRO	IPR011598:Helix-loop-helix DNA-binding	4	0.172413793	0.06433753	183	84	16659	4.33489461	1	0.83321999	60.501033
UP_SEQ_FEATURE	domain:Helix-loop-helix motif	4	0.172413793	0.10740448	193	114	19113	3.47477502	1	0.98125374	83.9289219
INTERPRO	IPR001092:Basic helix-loop-helix dimerisation region bHLH	4	0.172413793	0.12899683	183	114	16659	3.19413287	1	0.93856743	85.4728508
SMART	SM00353:HLH	4	0.172413793	0.20405873	125	114	9079	2.54849123	1	0.91498118	92.345444
UP_SEQ_FEATURE	DNA-binding region:Basic motif	4	0.172413793	0.22020935	193	161	19113	2.4603997	1	0.99957542	98.1721098
Annotation Cluster 22	Enrichment Score: 0.8710801964877731										
Category	Term	Count	%	PValue	List Total	Pop Hits	Pop Total	Fold Enrichme	Bonferroni	Benjamini	FDR
INTERPRO	IPR001007:von Willebrand factor, type C	3	0.129310345	0.07066421	183	40	16659	6.82745902	1	0.82476021	64.0724448
SMART	SM00214:VWC	3	0.129310345	0.10323421	125	40	9079	5.4474	0.99999225	0.85932318	70.6798808
GOTERM_MF_FAT	GO:0019838~growth factor binding	3	0.129310345	0.33399216	148	105	12983	2.50637066	1	0.95163838	99.577432
Annotation Cluster 23	Enrichment Score: 0.8624354053718727										
Category	Term	Count	%	PValue	List Total	Pop Hits	Pop Total	Fold Enrichme	Bonferroni	Benjamini	FDR
SP_PIR_KEYWORDS	Sodium	4	0.172413793	0.11007418	193	116	19235	3.4366625	1	0.53276691	78.2428503
SP_PIR_KEYWORDS	Sodium transport	4	0.172413793	0.11007418	193	116	19235	3.4366625	1	0.53276691	78.2428503
GOTERM_MF_FAT	GO:0031402~sodium ion binding	4	0.172413793	0.14698842	148	117	12983	2.999076	1	0.84166074	88.2135595
GOTERM_BP_FAT	GO:0006814~sodium ion transport	4	0.172413793	0.19934454	161	130	13528	2.58537984	1	0.93013742	97.2487941
Annotation Cluster 24	Enrichment Score: 0.8102144135667795										
Category	Term	Count	%	PValue	List Total	Pop Hits	Pop Total	Fold Enrichme	Bonferroni	Benjamini	FDR

GOTERM_BP_FAT	GO:0021987~cerebral cortex development	3	0.129310345	0.05780375	161	33	13528	7.63862225	1	0.69512601	61.7983587
GOTERM_BP_FAT	GO:0021543~pallium development	3	0.129310345	0.10272894	161	46	13528	5.47988118	1	0.79450547	82.6552166
GOTERM_BP_FAT	GO:0021537~telencephalon development	3	0.129310345	0.17932281	161	65	13528	3.87806976	1	0.91709671	95.8990529
GOTERM_BP_FAT	GO:0030900~forebrain development	3	0.129310345	0.53933426	161	152	13528	1.65838509	1	0.99162362	99.9996371
Annotation Cluster 25	Enrichment Score: 0.7550615131408012										
Category	Term	Count	%	PValue	List Total	Pop Hits	Pop Total	Fold Enrichme	Bonferroni	Benjamini	FDR
GOTERM_MF_FAT	GO:0016251~general RNA polymerase II transcription factor ac	3	0.129310345	0.10979878	148	50	12983	5.26337838	1	0.80885864	79.0760073
GOTERM_BP_FAT	GO:0006367~transcription initiation from RNA polymerase II pr	3	0.129310345	0.19212346	161	68	13528	3.70697844	1	0.92811833	96.8191537
GOTERM_BP_FAT	GO:0006352~transcription initiation	3	0.129310345	0.2574172	161	83	13528	3.03704258	1	0.95115419	99.1852495
Annotation Cluster 26	Enrichment Score: 0.7451508514943561										
Category	Term	Count	%	PValue	List Total	Pop Hits	Pop Total	Fold Enrichme	Bonferroni	Benjamini	FDR
GOTERM_BP_FAT	GO:0030218~erythrocyte differentiation	3	0.129310345	0.09166708	161	43	13528	5.86219847	1	0.77132315	78.8567675
GOTERM_BP_FAT	GO:0034101~erythrocyte homeostasis	3	0.129310345	0.11413457	161	49	13528	5.14437825	1	0.82217595	85.8948962
GOTERM_BP_FAT	GO:0030099~myeloid cell differentiation	3	0.129310345	0.30127435	161	93	13528	2.71047886	1	0.95933534	99.6953931
GOTERM_BP_FAT	GO:0048872~homeostasis of number of cells	3	0.129310345	0.33174458	161	100	13528	2.52074534	1	0.96329137	99.8518224
Annotation Cluster 27	Enrichment Score: 0.673093223172352										
Category	Term	Count	%	PValue	List Total	Pop Hits	Pop Total	Fold Enrichme	Bonferroni	Benjamini	FDR
GOTERM_CC_FAT	GO:0030176~integral to endoplasmic reticulum membrane	3	0.129310345	0.08580412	134	47	12782	6.08859956	1	0.65643179	69.0877294
GOTERM_CC_FAT	GO:0031227~intrinsic to endoplasmic reticulum membrane	3	0.129310345	0.14315315	134	64	12782	4.4713153	1	0.77668775	86.7589727
GOTERM_CC_FAT	GO:0031301~integral to organelle membrane	3	0.129310345	0.36700519	134	123	12782	2.32653804	1	0.93432075	99.7482465
GOTERM_CC_FAT	GO:0031300~intrinsic to organelle membrane	3	0.129310345	0.45044919	134	146	12782	1.96002862	1	0.95927718	99.9604132
Annotation Cluster 28	Enrichment Score: 0.6032911144651439										
Category	Term	Count	%	PValue	List Total	Pop Hits	Pop Total	Fold Enrichme	Bonferroni	Benjamini	FDR
GOTERM_BP_FAT	GO:0045859~regulation of protein kinase activity	7	0.301724138	0.22474223	161	345	13528	1.70485192	1	0.93891481	98.365942
GOTERM_BP_FAT	GO:0043549~regulation of kinase activity	7	0.301724138	0.24799225	161	357	13528	1.64754597	1	0.94852033	99.0010382
GOTERM_BP_FAT	GO:0051338~regulation of transferase activity	7	0.301724138	0.27797379	161	372	13528	1.58111267	1	0.95579177	99.4824133
Annotation Cluster 29	Enrichment Score: 0.562751093680959										
Category	Term	Count	%	PValue	List Total	Pop Hits	Pop Total	Fold Enrichme	Bonferroni	Benjamini	FDR
SP_PIR_KEYWORDS	Homeobox	5	0.215517241	0.22307536	193	242	19235	2.05915728	1	0.7322202	96.3163936
INTERPRO	IPR017970:Homeobox, conserved site	5	0.215517241	0.24567666	183	232	16659	1.96191351	1	0.9894966	98.0515351
INTERPRO	IPR001356:Homeobox	5	0.215517241	0.25560734	183	235	16659	1.93686781	1	0.98981405	98.3808062
SMART	SM00389:HOX	5	0.215517241	0.40050394	125	235	9079	1.5453617	1	0.98999823	99.6853169
Annotation Cluster 30	Enrichment Score: 0.5336485851628571										
Category	Term	Count	%	PValue	List Total	Pop Hits	Pop Total	Fold Enrichme	Bonferroni	Benjamini	FDR
GOTERM_CC_FAT	GO:0030665~clathrin coated vesicle membrane	3	0.129310345	0.10514132	134	53	12782	5.39932413	1	0.7178888	76.6319142
GOTERM_CC_FAT	GO:0030662~coated vesicle membrane	3	0.129310345	0.17604916	134	73	12782	3.92005725	1	0.81569461	92.0672778
GOTERM_CC_FAT	GO:0030659~cytoplasmic vesicle membrane	3	0.129310345	0.42566426	134	139	12782	2.0587351	1	0.95455588	99.9294875
GOTERM_CC_FAT	GO:0012506~vesicle membrane	3	0.129310345	0.46778317	134	151	12782	1.89512701	1	0.96329677	99.9739749
GOTERM_CC_FAT	GO:0044433~cytoplasmic vesicle part	3	0.129310345	0.58242222	134	187	12782	1.53028973	1	0.97503626	99.9989118
Annotation Cluster 31	Enrichment Score: 0.5329403095521158										
Category	Term	Count	%	PValue	List Total	Pop Hits	Pop Total	Fold Enrichme	Bonferroni	Benjamini	FDR
GOTERM_BP_FAT	GO:0051603~proteolysis involved in cellular protein catabolic p	10	0.431034483	0.28003707	161	600	13528	1.40041408	1	0.95369511	99.5058065
GOTERM_BP_FAT	GO:0044257~cellular protein catabolic process	10	0.431034483	0.28484052	161	603	13528	1.39344684	1	0.95543496	99.556482
GOTERM_BP_FAT	GO:0030163~protein catabolic process	10	0.431034483	0.31576339	161	622	13528	1.35088175	1	0.95918133	99.7828985
Annotation Cluster 32	Enrichment Score: 0.4868669743965641										
Category	Term	Count	%	PValue	List Total	Pop Hits	Pop Total	Fold Enrichme	Bonferroni	Benjamini	FDR
GOTERM_BP_FAT	GO:0051327~M phase of meiotic cell cycle	3	0.129310345	0.32307074	161	98	13528	2.57218912	1	0.9600878	99.8174841
GOTERM_BP_FAT	GO:0007126~meiosis	3	0.129310345	0.32307074	161	98	13528	2.57218912	1	0.9600878	99.8174841
GOTERM_BP_FAT	GO:0051321~meiotic cell cycle	3	0.129310345	0.33174458	161	100	13528	2.52074534	1	0.96329137	99.8518224

Annotation Cluster 33 Enrichment Score: 0.4856004309212771

Category	Term	Count	%	PValue	List Total	Pop Hits	Pop Total	Fold Enrichme	Bonferroni	Benjamini	FDR
GOTERM_BP_FAT	GO:0045664~regulation of neuron differentiation	4	0.172413793	0.20847703	161	133	13528	2.527063	1	0.93392942	97.714383
GOTERM_BP_FAT	GO:0050767~regulation of neurogenesis	4	0.172413793	0.31323726	161	166	13528	2.02469505	1	0.96067585	99.7695758
GOTERM_BP_FAT	GO:0051960~regulation of nervous system development	4	0.172413793	0.39936037	161	192	13528	1.7505176	1	0.97353788	99.9735736
GOTERM_BP_FAT	GO:0060284~regulation of cell development	4	0.172413793	0.43782614	161	205	13528	1.63950917	1	0.98010078	99.9909323

Annotation Cluster 34 Enrichment Score: 0.47141765202592645

Category	Term	Count	%	PValue	List Total	Pop Hits	Pop Total	Fold Enrichme	Bonferroni	Benjamini	FDR
GOTERM_BP_FAT	GO:0042325~regulation of phosphorylation	8	0.344827586	0.3139009	161	466	13528	1.4424866	1	0.96008763	99.7731482
GOTERM_BP_FAT	GO:0051174~regulation of phosphorus metabolic process	8	0.344827586	0.35032993	161	485	13528	1.38597682	1	0.96650683	99.9060691
GOTERM_BP_FAT	GO:0019220~regulation of phosphate metabolic process	8	0.344827586	0.35032993	161	485	13528	1.38597682	1	0.96650683	99.9060691

Annotation Cluster 35 Enrichment Score: 0.4518060033034458

Category	Term	Count	%	PValue	List Total	Pop Hits	Pop Total	Fold Enrichme	Bonferroni	Benjamini	FDR
GOTERM_BP_FAT	GO:0006606~protein import into nucleus	3	0.129310345	0.27058826	161	86	13528	2.93109923	1	0.95475605	99.3898816
GOTERM_BP_FAT	GO:0051170~nuclear import	3	0.129310345	0.2793672	161	88	13528	2.86448334	1	0.95550787	99.4983227
GOTERM_BP_FAT	GO:0034504~protein localization in nucleus	3	0.129310345	0.30564452	161	94	13528	2.68164398	1	0.9596645	99.7247659
GOTERM_BP_FAT	GO:0017038~protein import	3	0.129310345	0.46050486	161	131	13528	1.92423309	1	0.98349114	99.995339
GOTERM_BP_FAT	GO:0033365~protein localization in organelle	3	0.129310345	0.51764828	161	146	13528	1.72653791	1	0.98987973	99.9992368

Annotation Cluster 36 Enrichment Score: 0.4438401259214148

Category	Term	Count	%	PValue	List Total	Pop Hits	Pop Total	Fold Enrichme	Bonferroni	Benjamini	FDR
GOTERM_BP_FAT	GO:0030097~hemopoiesis	5	0.215517241	0.30539431	161	236	13528	1.78018739	1	0.9604864	99.7231586
GOTERM_BP_FAT	GO:0048534~hemopoietic or lymphoid organ development	5	0.215517241	0.36981915	161	260	13528	1.6158624	1	0.96561726	99.9425847
GOTERM_BP_FAT	GO:0002520~immune system development	5	0.215517241	0.41269507	161	276	13528	1.52218922	1	0.97589842	99.9816153

Annotation Cluster 37 Enrichment Score: 0.4346474424225064

Category	Term	Count	%	PValue	List Total	Pop Hits	Pop Total	Fold Enrichme	Bonferroni	Benjamini	FDR
SP_PIR_KEYWORDS	gtp-binding	6	0.25862069	0.23270345	193	329	19235	1.81756618	1	0.74220025	96.870735
GOTERM_MF_FAT	GO:0005525~GTP binding	6	0.25862069	0.41329397	148	372	12983	1.41488666	1	0.97343848	99.9231955
GOTERM_MF_FAT	GO:0032561~guanyl ribonucleotide binding	6	0.25862069	0.43568657	148	382	12983	1.37784774	1	0.97216972	99.9544919
GOTERM_MF_FAT	GO:0019001~guanyl nucleotide binding	6	0.25862069	0.43568657	148	382	12983	1.37784774	1	0.97216972	99.9544919

Annotation Cluster 38 Enrichment Score: 0.41611165209971124

Category	Term	Count	%	PValue	List Total	Pop Hits	Pop Total	Fold Enrichme	Bonferroni	Benjamini	FDR
GOTERM_MF_FAT	GO:0019787~small conjugating protein ligase activity	4	0.172413793	0.29045722	148	166	12983	2.11380658	1	0.94937774	99.0097477
GOTERM_MF_FAT	GO:0016881~acid-amino acid ligase activity	4	0.172413793	0.39850563	148	201	12983	1.74573081	1	0.97311354	99.8926532
GOTERM_MF_FAT	GO:0016879~ligase activity, forming carbon-nitrogen bonds	4	0.172413793	0.48769511	148	231	12983	1.51901252	1	0.98215219	99.9876029

Annotation Cluster 39 Enrichment Score: 0.37779431081434

Category	Term	Count	%	PValue	List Total	Pop Hits	Pop Total	Fold Enrichme	Bonferroni	Benjamini	FDR
UP_SEQ_FEATURE	repeat:3	4	0.172413793	0.36994915	193	214	19113	1.85104838	1	0.99999083	99.9408442
UP_SEQ_FEATURE	repeat:1	4	0.172413793	0.44166577	193	243	19113	1.63014137	1	0.99999815	99.9915358
UP_SEQ_FEATURE	repeat:2	4	0.172413793	0.45017492	193	246	19113	1.61026159	1	0.99999782	99.993389

Annotation Cluster 40 Enrichment Score: 0.352073143166678

Category	Term	Count	%	PValue	List Total	Pop Hits	Pop Total	Fold Enrichme	Bonferroni	Benjamini	FDR
UP_SEQ_FEATURE	repeat:TPR 3	3	0.129310345	0.4045719	193	138	19113	2.15284974	1	0.99999371	99.9761739
UP_SEQ_FEATURE	repeat:TPR 2	3	0.129310345	0.44950228	193	151	19113	1.96750506	1	0.99999826	99.9932576
UP_SEQ_FEATURE	repeat:TPR 1	3	0.129310345	0.44950228	193	151	19113	1.96750506	1	0.99999826	99.9932576
SP_PIR_KEYWORDS	tpr repeat	3	0.129310345	0.47780194	193	160	19235	1.86868523	1	0.92041689	99.979602

Annotation Cluster 41 Enrichment Score: 0.31467056871158267

Category	Term	Count	%	PValue	List Total	Pop Hits	Pop Total	Fold Enrichme	Bonferroni	Benjamini	FDR
UP_SEQ_FEATURE	domain:Ig-like C2-type 3	3	0.129310345	0.34724542	193	122	19113	2.43519069	1	0.99998241	99.8954397
UP_SEQ_FEATURE	domain:Ig-like C2-type 1	3	0.129310345	0.57102641	193	190	19113	1.56364876	1	0.99999986	99.9998781
UP_SEQ_FEATURE	domain:Ig-like C2-type 2	3	0.129310345	0.57371416	193	191	19113	1.55546212	1	0.99999979	99.9998899

Annotation Cluster 42 Enrichment Score: 0.31065741348313824

Category	Term	Count	%	PValue	List Total	Pop Hits	Pop Total	Fold Enrichme	Bonferroni	Benjamini	FDR
GOTERM_MF_FAT	GO:0004725~protein tyrosine phosphatase activity	3	0.129310345	0.32984728	148	104	12983	2.53047037	1	0.95290387	99.5406589
SP_PIR_KEYWORDS	protein phosphatase	3	0.129310345	0.37321388	193	130	19235	2.29992029	1	0.86894027	99.7779052
GOTERM_BP_FAT	GO:0006470~protein amino acid dephosphorylation	3	0.129310345	0.46833688	161	133	13528	1.89529725	1	0.98431326	99.9963201
GOTERM_BP_FAT	GO:0016311~dephosphorylation	3	0.129310345	0.54661456	161	154	13528	1.63684762	1	0.99202008	99.9997195
GOTERM_MF_FAT	GO:0004721~phosphoprotein phosphatase activity	3	0.129310345	0.55872233	148	165	12983	1.59496314	1	0.99049203	99.9983344
GOTERM_MF_FAT	GO:0016791~phosphatase activity	3	0.129310345	0.77687158	148	249	12983	1.05690329	1	0.9991861	99.9999998

Annotation Cluster 43 Enrichment Score: 0.3004044434005013

Category	Term	Count	%	PValue	List Total	Pop Hits	Pop Total	Fold Enrichme	Bonferroni	Benjamini	FDR
GOTERM_BP_FAT	GO:0050953~sensory perception of light stimulus	5	0.215517241	0.2527938	161	216	13528	1.94501955	1	0.94927774	99.0992803
GOTERM_BP_FAT	GO:0007601~visual perception	5	0.215517241	0.2527938	161	216	13528	1.94501955	1	0.94927774	99.0992803
GOTERM_BP_FAT	GO:0007600~sensory perception	5	0.215517241	0.98826879	161	810	13528	0.51867188	1	1	100
GOTERM_BP_FAT	GO:0050890~cognition	5	0.215517241	0.99534654	161	909	13528	0.46218286	1	1	100

Annotation Cluster 44 Enrichment Score: 0.2818397489810651

Category	Term	Count	%	PValue	List Total	Pop Hits	Pop Total	Fold Enrichme	Bonferroni	Benjamini	FDR
UP_SEQ_FEATURE	domain:Fibronectin type-III 2	3	0.129310345	0.376176	193	130	19113	2.2853328	1	0.9999908	99.949581
UP_SEQ_FEATURE	domain:Fibronectin type-III 1	3	0.129310345	0.37975678	193	131	19113	2.26788751	1	0.9999898	99.9540412
INTERPRO	IPR008957:Fibronectin, type III-like fold	3	0.129310345	0.59974593	183	184	16659	1.48423022	1	0.99998117	99.9997212
INTERPRO	IPR003961:Fibronectin, type III	3	0.129310345	0.61724804	183	190	16659	1.43735979	1	0.99997914	99.9998507
SMART	SM00060:FN3	3	0.129310345	0.73701718	125	190	9079	1.14682105	1	0.99987852	99.9999706

Annotation Cluster 45 Enrichment Score: 0.2793163141531406

Category	Term	Count	%	PValue	List Total	Pop Hits	Pop Total	Fold Enrichme	Bonferroni	Benjamini	FDR
INTERPRO	IPR013098:Immunoglobulin I-set	4	0.172413793	0.19185998	183	138	16659	2.6386315	1	0.97628723	94.8978947
INTERPRO	IPR013151:Immunoglobulin	4	0.172413793	0.37897485	183	202	16659	1.80262944	1	0.99868841	99.8711464
INTERPRO	IPR003598:Immunoglobulin subtype 2	4	0.172413793	0.38857452	183	205	16659	1.7762495	1	0.99867334	99.8963446
SMART	SM00408:IGc2	4	0.172413793	0.53398317	125	205	9079	1.41720976	1	0.99824134	99.9815413
SP_PIR_KEYWORDS	Immunoglobulin domain	4	0.172413793	0.85152948	193	470	19235	0.84819755	1	0.99693786	100
INTERPRO	IPR007110:Immunoglobulin-like	4	0.172413793	0.91443715	183	501	16659	0.72680868	1	1	100
INTERPRO	IPR013783:Immunoglobulin-like fold	4	0.172413793	0.94370352	183	553	16659	0.658465	1	1	100

Annotation Cluster 46 Enrichment Score: 0.2774631076819198

Category	Term	Count	%	PValue	List Total	Pop Hits	Pop Total	Fold Enrichme	Bonferroni	Benjamini	FDR
GOTERM_BP_FAT	GO:0008380~RNA splicing	5	0.215517241	0.43392838	161	284	13528	1.47931065	1	0.9800508	99.989861
GOTERM_BP_FAT	GO:0006397~mRNA processing	5	0.215517241	0.52872611	161	321	13528	1.3087982	1	0.99058939	99.9994757
GOTERM_BP_FAT	GO:0016071~mRNA metabolic process	5	0.215517241	0.64115327	161	370	13528	1.13547087	1	0.99717615	99.9999936

Annotation Cluster 47 Enrichment Score: 0.2640775207278486

Category	Term	Count	%	PValue	List Total	Pop Hits	Pop Total	Fold Enrichme	Bonferroni	Benjamini	FDR
INTERPRO	IPR005821:Ion transport	3	0.129310345	0.33863363	183	110	16659	2.48271237	1	0.99794277	99.689623
SP_PIR_KEYWORDS	voltage-gated channel	3	0.129310345	0.44276746	193	150	19235	1.99326425	1	0.90444823	99.9523098
GOTERM_MF_FAT	GO:0022843~voltage-gated cation channel activity	3	0.129310345	0.49809486	148	147	12983	1.79026475	1	0.98294023	99.9905913
GOTERM_MF_FAT	GO:0005244~voltage-gated ion channel activity	3	0.129310345	0.65122513	148	195	12983	1.3495842	1	0.99550498	99.9999296
GOTERM_MF_FAT	GO:0022832~voltage-gated channel activity	3	0.129310345	0.65122513	148	195	12983	1.3495842	1	0.99550498	99.9999296
GOTERM_MF_FAT	GO:0005261~cation channel activity	3	0.129310345	0.82196764	148	275	12983	0.95697789	1	0.99950357	100

Annotation Cluster 48 Enrichment Score: 0.26244957525226714

Category	Term	Count	%	PValue	List Total	Pop Hits	Pop Total	Fold Enrichme	Bonferroni	Benjamini	FDR
GOTERM_BP_FAT	GO:0000280~nuclear division	4	0.172413793	0.48483727	161	220	13528	1.52772445	1	0.98631633	99.9977892
GOTERM_BP_FAT	GO:0007067~mitosis	4	0.172413793	0.48483727	161	220	13528	1.52772445	1	0.98631633	99.9977892
GOTERM_BP_FAT	GO:0000087~M phase of mitotic cell cycle	4	0.172413793	0.49617214	161	224	13528	1.50044366	1	0.98757525	99.9984569
GOTERM_BP_FAT	GO:0048285~organelle fission	4	0.172413793	0.51093812	161	229	13528	1.46768288	1	0.98921954	99.9990459
GOTERM_BP_FAT	GO:0000278~mitotic cell cycle	4	0.172413793	0.81763494	161	370	13528	0.9083767	1	0.99978658	100

Annotation Cluster 49 Enrichment Score: 0.1963851065900636

Category	Term	Count	%	PValue	List Total	Pop Hits	Pop Total	Fold Enrichme	Bonferroni	Benjamini	FDR
GOTERM_MF_FAT	GO:0032555~purine ribonucleotide binding	21	0.905172414	0.61060471	148	1836	12983	1.00336734	1	0.99270198	99.9996903
GOTERM_MF_FAT	GO:0032553~ribonucleotide binding	21	0.905172414	0.61060471	148	1836	12983	1.00336734	1	0.99270198	99.9996903
GOTERM_MF_FAT	GO:0017076~purine nucleotide binding	21	0.905172414	0.69075555	148	1918	12983	0.96047051	1	0.99706444	99.999986

Annotation Cluster 50 Enrichment Score: 0.19164962245570655

Category	Term	Count	%	PValue	List Total	Pop Hits	Pop Total	Fold Enrichme	Bonferroni	Benjamini	FDR
GOTERM_BP_FAT	GO:0030334~regulation of cell migration	3	0.129310345	0.59697341	161	169	13528	1.49156529	1	0.99516677	99.9999582
GOTERM_BP_FAT	GO:0040012~regulation of locomotion	3	0.129310345	0.66625793	161	192	13528	1.3128882	1	0.99771491	99.999998
GOTERM_BP_FAT	GO:0051270~regulation of cell motion	3	0.129310345	0.66904257	161	193	13528	1.30608567	1	0.99775611	99.9999983

Annotation Cluster 51 Enrichment Score: 0.15929412198874976

Category	Term	Count	%	PValue	List Total	Pop Hits	Pop Total	Fold Enrichme	Bonferroni	Benjamini	FDR
INTERPRO	IPR006210:EGF-like	3	0.129310345	0.64780643	183	201	16659	1.35869831	1	0.99998481	99.9999533
SP_PIR_KEYWORDS	egf-like domain	3	0.129310345	0.67147577	193	230	19235	1.29995494	1	0.97588095	99.9999524
SMART	SM00181:EGF	3	0.129310345	0.76496582	125	201	9079	1.0840597	1	0.99989888	99.9999917

Annotation Cluster 52 Enrichment Score: 0.15700902721701027

Category	Term	Count	%	PValue	List Total	Pop Hits	Pop Total	Fold Enrichme	Bonferroni	Benjamini	FDR
UP_SEQ_FEATURE	repeat:ANK 3	3	0.129310345	0.57769046	193	194	19113	1.53140858	1	0.99999972	99.9999053
UP_SEQ_FEATURE	repeat:ANK 1	3	0.129310345	0.67719048	193	231	19113	1.28611803	1	1	99.9999987
UP_SEQ_FEATURE	repeat:ANK 2	3	0.129310345	0.67948773	193	232	19113	1.28057441	1	1	99.9999989
SP_PIR_KEYWORDS	ank repeat	3	0.129310345	0.69403908	193	240	19235	1.24579016	1	0.97901132	99.9999812
INTERPRO	IPR002110:Ankyrin	3	0.129310345	0.73655341	183	238	16659	1.1474721	1	0.99999859	99.9999992
SMART	SM00248:ANK	3	0.129310345	0.84094969	125	238	9079	0.91552941	1	0.99998381	99.9999999

Annotation Cluster 53 Enrichment Score: 0.1330444582655304

Category	Term	Count	%	PValue	List Total	Pop Hits	Pop Total	Fold Enrichme	Bonferroni	Benjamini	FDR
GOTERM_BP_FAT	GO:0006605~protein targeting	4	0.172413793	0.47093949	161	215	13528	1.56325293	1	0.98445993	99.9966007
GOTERM_BP_FAT	GO:0006886~intracellular protein transport	4	0.172413793	0.82326517	161	374	13528	0.89866144	1	0.99979697	100
GOTERM_BP_FAT	GO:0034613~cellular protein localization	4	0.172413793	0.86868469	161	411	13528	0.81776005	1	0.99993514	100
GOTERM_BP_FAT	GO:0070727~cellular macromolecule localization	4	0.172413793	0.87187686	161	414	13528	0.81183425	1	0.99994002	100

Annotation Cluster 54 Enrichment Score: 0.11337456940736151

Category	Term	Count	%	PValue	List Total	Pop Hits	Pop Total	Fold Enrichme	Bonferroni	Benjamini	FDR
SP_PIR_KEYWORDS	ionic channel	4	0.172413793	0.61798516	193	318	19235	1.25362531	1	0.96329075	99.999658
GOTERM_MF_FAT	GO:0022836~gated channel activity	4	0.172413793	0.68592592	148	310	12983	1.13190933	1	0.9971105	99.9999828
GOTERM_MF_FAT	GO:0005216~ion channel activity	4	0.172413793	0.81723502	148	386	12983	0.90904635	1	0.99955149	100
GOTERM_MF_FAT	GO:0022838~substrate specific channel activity	4	0.172413793	0.83306507	148	398	12983	0.88163792	1	0.99953598	100
GOTERM_MF_FAT	GO:0015267~channel activity	4	0.172413793	0.85004355	148	412	12983	0.85167935	1	0.99967299	100
GOTERM_MF_FAT	GO:0022803~passive transmembrane transporter activity	4	0.172413793	0.85119733	148	413	12983	0.84961717	1	0.99964761	100

Annotation Cluster 55 Enrichment Score: 0.10008748287441796

Category	Term	Count	%	PValue	List Total	Pop Hits	Pop Total	Fold Enrichme	Bonferroni	Benjamini	FDR
UP_SEQ_FEATURE	domain:PH	3	0.129310345	0.69077556	193	237	19113	1.25355808	1	1	99.9999994
INTERPRO	IPR001849:Pleckstrin homology	3	0.129310345	0.80884271	183	277	16659	0.98591466	1	0.99999992	100
SMART	SM00233:PH	3	0.129310345	0.89647155	125	277	9079	0.78662816	1	0.99999748	100

Annotation Cluster 56 Enrichment Score: 0.09730435111222278

Category	Term	Count	%	PValue	List Total	Pop Hits	Pop Total	Fold Enrichme	Bonferroni	Benjamini	FDR
GOTERM_BP_FAT	GO:0043066~negative regulation of apoptosis	4	0.172413793	0.7935868	161	354	13528	0.94943327	1	0.99970866	100
GOTERM_BP_FAT	GO:0043069~negative regulation of programmed cell death	4	0.172413793	0.80136964	161	359	13528	0.93620997	1	0.99973423	100
GOTERM_BP_FAT	GO:0060548~negative regulation of cell death	4	0.172413793	0.80289661	161	360	13528	0.93360939	1	0.99973535	100

Annotation Cluster 57 Enrichment Score: 0.08321091797687243

Category	Term	Count	%	PValue	List Total	Pop Hits	Pop Total	Fold Enrichme	Bonferroni	Benjamini	FDR
GOTERM_MF_FAT	GO:0005524~ATP binding	15	0.646551724	0.79752797	148	1477	12983	0.89089004	1	0.99942511	100

GOTERM_MF_FAT	GO:0032559~adenyl ribonucleotide binding	15	0.646551724	0.81332215	148	1497	12983	0.87898771	1	0.99955855	100
GOTERM_MF_FAT	GO:0030554~adenyl nucleotide binding	15	0.646551724	0.86767828	148	1577	12983	0.83439733	1	0.99975873	100

Annotation Cluster 58 Enrichment Score: 0.06142510204547453

Category	Term	Count	%	PValue	List Total	Pop Hits	Pop Total	Fold Enrichme	Bonferroni	Benjamini	FDR
GOTERM_BP_FAT	GO:0016477~cell migration	3	0.129310345	0.84156931	161	276	13528	0.91331353	1	0.9998722	100
GOTERM_BP_FAT	GO:0048870~cell motility	3	0.129310345	0.88169302	161	307	13528	0.82108969	1	0.99995548	100
GOTERM_BP_FAT	GO:0051674~localization of cell	3	0.129310345	0.88169302	161	307	13528	0.82108969	1	0.99995548	100

Annotation Cluster 59 Enrichment Score: 0.050496228310429915

Category	Term	Count	%	PValue	List Total	Pop Hits	Pop Total	Fold Enrichme	Bonferroni	Benjamini	FDR
GOTERM_BP_FAT	GO:0043065~positive regulation of apoptosis	4	0.172413793	0.88777655	161	430	13528	0.78162646	1	0.99996383	100
GOTERM_BP_FAT	GO:0043068~positive regulation of programmed cell death	4	0.172413793	0.89055529	161	433	13528	0.77621104	1	0.99996651	100
GOTERM_BP_FAT	GO:0010942~positive regulation of cell death	4	0.172413793	0.89237369	161	435	13528	0.77264225	1	0.99996775	100

Annotation Cluster 60 Enrichment Score: 0.034777103782248894

Category	Term	Count	%	PValue	List Total	Pop Hits	Pop Total	Fold Enrichme	Bonferroni	Benjamini	FDR
GOTERM_BP_FAT	GO:0042981~regulation of apoptosis	7	0.301724138	0.91965055	161	804	13528	0.73155959	1	0.99998916	100
GOTERM_BP_FAT	GO:0043067~regulation of programmed cell death	7	0.301724138	0.92396471	161	812	13528	0.72435211	1	0.99999078	100
GOTERM_BP_FAT	GO:0010941~regulation of cell death	7	0.301724138	0.92552993	161	815	13528	0.72168578	1	0.99999123	100
